# Survival analysis of adjuvant endocrine therapy in HER2 positive early breast cancer patients with low ER positivity

**DOI:** 10.1186/s13058-025-02157-9

**Published:** 2025-12-29

**Authors:** Hsuan-Hsuan Yu, Deanna Gracia, Ta-Chung Chao, Chan-Heng Ho, Hao-Yang Chen, Ling-Ming Tseng, Chi-Cheng Huang

**Affiliations:** 1 School of Medicine, College of Medicine, National Yang Ming Chiao Tung University, Taipei City, Taiwan; 2 Division of Breast Surgery, Department of Surgery, Taipei Veterans General Hospital, Taipei City, Taiwan; 3 Department of Public Health, College of Public Health, National Taiwan University, Taipei City, Taiwan; 4 Comprehensive Breast Health Center, Taipei Veterans General Hospital, Taipei City, Taiwan; 5 Division of Cancer Prevention, Department of Oncology, Taipei Veterans General Hospital, Taipei City, Taiwan; 6 Institute of Epidemiology and Preventive Medicine, College of Public Health, National Taiwan University, Taipei City, Taiwan

**Keywords:** Low ER positivity, HER2 positivity, Survival, Early breast cancer, Endocrine therapy

## Abstract

**Background:**

Optimal treatment strategies for early-stage human epidermal growth factor receptor 2 (HER2) positive breast cancer with low estrogen receptor (ER) expression (1–9%) remain unclear. While endocrine therapy (ET) is standard for ER-positive disease, its benefit in ER-low tumors, particularly with concurrent HER2 overexpression, is less established.

**Methods:**

We conducted a retrospective cohort study using Taiwan’s national cancer registry (TCR), identifying 10,408 patients with HER2-positive early breast cancer diagnosed between 2011 and 2019. Of these, 1436 (15.5%) had low ER positivity. Patients were stratified by ER level (1–9% vs. ≥ 10%) and ET use. Overall survival (OS), breast cancer-specific survival (BCSS), and recurrence-free survival (RFS) were evaluated via Kaplan–Meier and Cox regression analyses. Progesterone receptor (PR) status was also assessed.

**Results:**

Adjuvant ET significantly improved OS, BCSS, and RFS in both ER subgroups (all p < 0.05). In ER-low patients, ET was associated with improved OS (8.2 ± 0.1 years vs. 7.9 ± 0.1 years, 90.2% vs. 85.6%, p = 0.008), BCSS (8.4 ± 0.1 years vs. 8.2 ± 0.1 years, 93.9% vs. 89.7%, p = 0.005), and RFS (8.8 ± 0.1 years vs. 8.8 ± 0.1 years, 91.2% vs. 88.2%, p = 0.032) up to 10 years of follow-up. On multivariate analysis, PR positivity—not ER level—was an independent predictor of improved outcomes. Notably, PR-positive, ER-low patients had better OS with ET (92.1% vs. 86.7%, p = 0.022). Further subgroup analysis also showed improved OS, BCSS and RFS outcomes for those receiving longer ET duration (> 60 months) or for non-pathological complete response (pCR) patients.

**Conclusion:**

Adjuvant ET provides significant survival benefits in HER2-positive early breast cancer with low ER expression, particularly in PR-positive tumors. Despite this, 41.9% of eligible ER-low patients did not receive ET, highlighting a treatment gap. PR status may guide ET decisions, supporting individualized treatment approaches. Adjuvant ET also showed survival benefits in non-pCR patients with longer duration (> 60 months) associated with better survival outcomes. Although the survival benefit of adjuvant ET appeared greater in patients without pCR, the benefit in the pCR group could not be quantified due to the limited sample size. Importantly, our findings do not suggest withholding ET in patients achieving pCR.

**Supplementary Information:**

The online version contains supplementary material available at 10.1186/s13058-025-02157-9.

## Introduction

Breast cancer remains one of the most prevalent malignancies globally [1], encompassing a variety of immunohistochemical subtypes with distinct biological behaviors and therapeutic responses [2–5]. Among these, human epidermal growth factor receptor 2 (HER2)-positive breast cancer is recognized for its aggressive clinical course, and the advent of HER2-targeted therapies has significantly improved outcomes [6–8]. Endocrine therapy (ET) is standard for hormone receptor–positive early breast cancer, typically prescribed for tumors expressing estrogen receptor (ER) or progesterone receptor (PR). However, the crosstalk between ER and HER2 signaling pathways can induce endocrine resistance, highlighting the need for combined treatment strategies, as supported by the ASCO guidelines and reinforced by trials like ADAPT showing improved responses when HER2 blockade is paired with ET [9, 10]. However, when HER2 positivity is coupled with low ER expression (1–9%), treatment strategies become more complex and require careful consideration[11, 12].

Recent evidence indicates that breast cancers with low ER expression exhibit clinical behavior and prognosis more akin to ER‑negative disease than to ER‑positive tumors. A meta-analysis encompassing multiple retrospective cohorts found that ER‑low cases had significantly worse disease-free and overall survival compared to ER‑positive (> 10%) tumors, yet demonstrated no significant difference in these outcomes when compared with ER‑negative cases [13, 14].

Similarly, single institute cohort studies from Korea and China reported that ER‑low tumors had poorer survival than ER‑positive tumors, while outcomes were largely equivalent to ER‑negative tumors, and ET did not confer substantial benefit in ER‑low patients [15, 16]. A Swedish population‑based study treating ER‑low/HER2‑negative tumors like triple‑negative disease confirmed near‑identical overall survival and pathological complete response (pCR) rates between ER‑low and ER‑zero groups [17]. Collectively, these findings suggest that ER‑low breast cancers align more closely with ER‑negative biology and outcomes, which may attenuate the benefits of ET in this subgroup.

Adjuvant ET in patients with early-stage, HER2-positive breast cancer exhibiting low ER expression represents a clinically nuanced and evolving area of oncologic management. While a low ER positive status used to raise questions regarding the efficacy of ET, emerging evidence suggests a potential benefit, particularly when integrated with HER2-targeted therapies [18, 19]. A prospective cohort study by Xie et al. demonstrated improved breast cancer-specific survival (BCCS) with adjuvant ET, most notably with aromatase inhibitors (AIs) or sequential tamoxifen followed by AIs, even among patients with low ER-positive, HER2-positive disease [20]. The findings support the judicious use of ET in this subset of patients, with careful consideration of regimen selection to optimize clinical outcomes.

Despite the central role of endocrine treatment in ER-positive breast cancers, its benefit in ER-low tumors, especially those that also overexpress HER2, still remains contentious. For example, a large cohort study conducted in China reported that although patients with low ER expression received ET, no significant improvement in relapse-free survival (RFS) was observed [11]. This finding suggested that the biological behavior of HER2-positive, low ER-expressing tumors may necessitate a departure from conventional endocrine therapy and prompt the use of more targeted approaches.

Further prospective cohort studies and meta-analyses have underscored the need for individualized treatment regimens that take into account both HER2 and ER status. Although some evidence suggests limited ET benefits in this setting, definitive conclusions remain elusive, underscoring the need for further investigation.

This study critically evaluates adjuvant ET’s role in HER2-positive, low ER early breast cancer, aiming to clarify its clinical impact and guide optimized treatment strategies.

## Materials and methods

### Ethical approval and informed consent

The data for this study was derived from the Taiwan Cancer Registry—Long Form system [21], which includes records of women diagnosed with breast cancer in Taiwan. The Taiwan Cancer Registry (TCR) is a population-based registry with mandatory reporting of cancer diagnoses from all hospitals with 50 or more beds, ensuring near-complete national coverage. It collects detailed information on breast cancer through a long-form dataset, including tumor characteristics, stage, treatment modalities, and patient lifestyle factors. Outcomes such as recurrence are either reported directly or inferred through treatment patterns, while survival data is accurately captured via linkage with the National Death Registry. Additional integration with the National Health Insurance database enhances follow-up and treatment tracking. The registry is highly complete (> 98% case coverage), with high morphological verification rates (> 90%) and minimal reliance on death certificate-only cases (< 0.8%). Rigorous data quality assurance measures and nationwide hospital participation make the TCR a reliable and representative source for epidemiological and outcome research on breast cancer in Taiwan [22, 23].

The research protocol was approval from the institutional review board (IRB-TPEVGH protocol no. 2024–06-007B). Given the retrospective and anonymized nature of the study, informed consent was waived.

### Patients’ selection

Early breast cancer patients with HER2-positive status (defined as HER2 3 + or HER2 2 + with Fluorescence in situ hybridization [FISH] positivity) and ER-positive status (defined as ER > 1%) were enrolled in this study, from 2011 to 2019. We retrospectively followed up these patients and the censored date was December 31, 2022. According to the ASCO guidelines, ER positivity of 1–9% was classified as low ER status. In this study, patients were grouped into either ER ≥ 10% and ER 1–9%. Adjuvant endocrine therapy was defined as the administration of tamoxifen or aromatase inhibitors (AIs), including letrozole, anastrozole, and exemestane, following curative surgery. The detailed regimen and duration were presented in Supplemental Materials (Tables A4 and A4a).

In addition to ER grouping, various clinical features, including PR status, tumor grade, pathological stage, surgical procedure, radiotherapy, chemotherapy, targeted therapy, and whether patients received adjuvant endocrine therapy or not, were recorded and extracted. We compared the characteristics of patients with and without adjuvant ET among HER2-positive early breast cancer patients with low ER expression.

### Treatment regimens and duration

Data regarding systemic therapy were extracted from the TCR, which recorded detailed treatment regimens including chemotherapy,, and targeted therapy. Endocrine therapy agents included tamoxifen and aromatase inhibitors (letrozole, anastrozole, and exemestane), either as monotherapy or in sequential/combined regimens. Chemotherapy regimens were classified into taxanes, anthracyclines, alkylating agents, as well as their combinations. Targeted therapies primarily referred to trastuzumab, as pertuzumab was not reimbursed by the National Health Insurance and therefore not routinely documented in the registry. Treatment duration was categorized as ≤ 24 months, 25–60 months, and > 60 months, based on prescription data. Rigorous quality assurance measures and mandatory nationwide hospital participation ensure high validity and representativeness of these treatment data. The detailed systemic regimen and duration were presented in Supplemental Materials (Tables A6 andA6a).

### Survival analysis

Overall survival (OS) was defined as the duration from diagnosis to death from any cause, while breast cancer-specific survival (BCSS) referred to the period from diagnosis to death specifically attributable to breast cancer. RFS was the interval from diagnosis to the occurrence of the first recurrence or death, whichever occurred first. Censored date of the study was set to December 31, 2022, and the follow-up time was 10 years.

We conducted a series of survival analyses, with a mean follow-up period extending up to 10 years, to compare the outcomes of adjuvant ET in HER2-positive early breast cancer patients with ER positivity of ≥ 10% and ER positivity of 1–9%. Additionally, we evaluated the impact of PR on survival outcomes in HER2-positive early breast cancer patients with low ER status. Among PR-positive and PR-negative patients, survival outcomes were further compared, stratified by the conduct of adjuvant ET. Further subgroup analyses, such as the impact of neoadjuvant/adjuvant therapy, ER ultra-low (≤ 5%), and pCR status upon survival outcomes were reported in Supplement Materials.

### Primary and secondary endpoints

The primary endpoint was to assess the survival outcomes, including OS, BCSS, and RFS, in HER2-positive early breast cancer patients with low ER positivity, with or without adjuvant ET. The secondary endpoint was the prognostic role of PR in HER2-positive early breast cancer patients. Additional analysis, including subgroup (neoadjuvant/adjuvant treatment, ET duration and pCR/non-pCR status) and stratified analyses (PR status and ER ultra-low status) were also evaluated.

### Statistical analyses

Statistical analyses were conducted using Statistical Product and Service Solutions (SPSS) version 26.0 software (SPSS Inc., IBM, Armonk, NY, USA). Survival durations were estimated employing the Kaplan–Meier method, with comparisons made using the log-rank test. For multivariable analysis, Cox’s proportional hazards model was utilized. Statistical significance was defined as a p-value of less than 0.050.

## Results

This study enrolled 10,408 early HER2-positive and ER-positive breast cancer patients. Among these, 1,436 (15.5%) had low ER positivity (1–9%), and 7,838 (84.5%) had ER positivity ≥ 10% (Table 1).

**Table 1:** The characteristics of early breast cancer patients with HER2-positivity

	ER 1–9%		ER ≥ 10%	Total	*p*-value
ER positivity	1436 (15.5%)		7838 (84.5%)	9274	
PR	Negative	719 (50.1%)	1443 (18.4%)	2162	< 0.001
	Positive	717 (49.9%)	6395 (81.6%)	7112	
Grading	1	50 (3.5%)	778 (9.9%)	828	< 0.001
	2	581 (40.5%)	4280 (54.6%)	4861	
	3	805 (56.1%)	2780 (35.5%)	3585	
Staging	0	229 (16.0%)	922 (11.8%)	1151	< 0.001
	1	472 (32.9%)	2765 (35.3%)	3237	
	2	498 (34.7%)	2966 (37.8%)	3464	
	3	237 (16.5%)	1185 (15.1%)	1422	
Operation	Negative	8 (0.6%)	51 (0.7%)	59	0.682
	Positive	1428 (99.4%)	7787 (99.4%)	9215	
Radiotherapy	Negative	704 (49.0%)	3353 (42.8%)	4057	< 0.001
	Positive	732 (51.0%)	4485 (57.2%)	5217	
Chemotherapy	Negative	391 (27.2%)	2251 (28.7%)	2642	0.250
	Positive	1045 (72.8%)	5587 (71.3%)	6632	
Target therapy	Negative	668 (46.5%)	3712 (47.5%)	4380	0.557
	Positive	768 (53.5%)	4126 (52.6%)	4894	
Endocrine therapy	Negative	602 (41.9%)	694 (8.9%)	1296	< 0.001
	Positive	834 (58.1%)	7144 (91.2%)	7978	

In the ER ≥ 10% group, 81.6% were progesterone receptor (PR) positive, compared to 49.9% in the ER 1–9% group. Tumor grade III predominated in the ER 1–9% group (56.1%), whereas grade II was most common in the ER ≥ 10% group (54.6%). Most patients were diagnosed at stage II in both groups (34.7% in ER 1–9%, 37.8% in ER ≥ 10%). Nearly all patients underwent curative surgery (99.4% ER 1–9%, 99.4% ER ≥ 10%). Radiotherapy was administered to 51.0% of ER 1–9% and 57.2% of ER ≥ 10% patients. Chemotherapy was given to 72.8% of ER 1–9% and 71.3% of ER ≥ 10%. Targeted therapy rates were comparable (53.5% ER 1–9%, 52.6% ER ≥ 10%). Notably, 91.2% of ER ≥ 10% patients received adjuvant ET, while only 58.1% of ER 1–9% patients received ET.

Among ER 1–9% patients, those receiving adjuvant ET were more often PR positive (54.6%) than those who did not receive ET (43.9%). Grade III tumors were prevalent regardless of ET status (53.8% with ET, 59.1% without). Most patients receiving ET were diagnosed at stage I (36.1%), whereas stage II predominated in those without ET (34.9%). Mean ages were similar (54.3 ± 10.2 years with ET; 55.3 ± 10.7 years without). Radiotherapy was more frequent among ET recipients (56.2% vs. 43.7%). Chemotherapy rates were nearly identical (72.8% vs. 72.8%), and targeted therapy was slightly lower in ET recipients (51.3%) compared to non-recipients (56.5%) (Table 2).

**Table 2:** The characteristics of HER2-positivity in early breast cancer patients with low ER positivity

ER 1–9%				
		Endocrine therapy ( +)	Endocrine therapy (-)	*p*-value
Age		54.3 ± 10.2	55.3 ± 10.7	0.275
PR	Negative	379 (45.4%)	338 (56.2%)	< 0.001
	Positive	455 (54.6%)	264 (43.9%)	
Grade	1	40 (4.8%)	10 (1.7%)	0.002
	2	345 (41.4%)	236 (39.2%)	
	3	449 (53.8%)	356 (59.1%)	
Staging	0	121 (14.5%)	108 (17.9%)	0.007
	1	301 (36.1%)	171 (28.4%)	
	2	288 (34.5%)	210 (34.9%)	
	3	124 (14.9%)	113 (18.8%)	
Radiotherapy	Negative	365 (43.8%)	339 (56.3%)	< 0.001
	Positive	469 (56.2%)	263 (43.7%)	
Chemotherapy	Negative	227 (27.2%)	164 (27.2%)	0.992
	Positive	607 (72.8%)	438 (72.8%)	
Target therapy	Negative	406 (48.7%)	262 (43.5%)	0.053
	Positive	428 (51.3%)	340 (56.5%)	

**Fig. 1:**
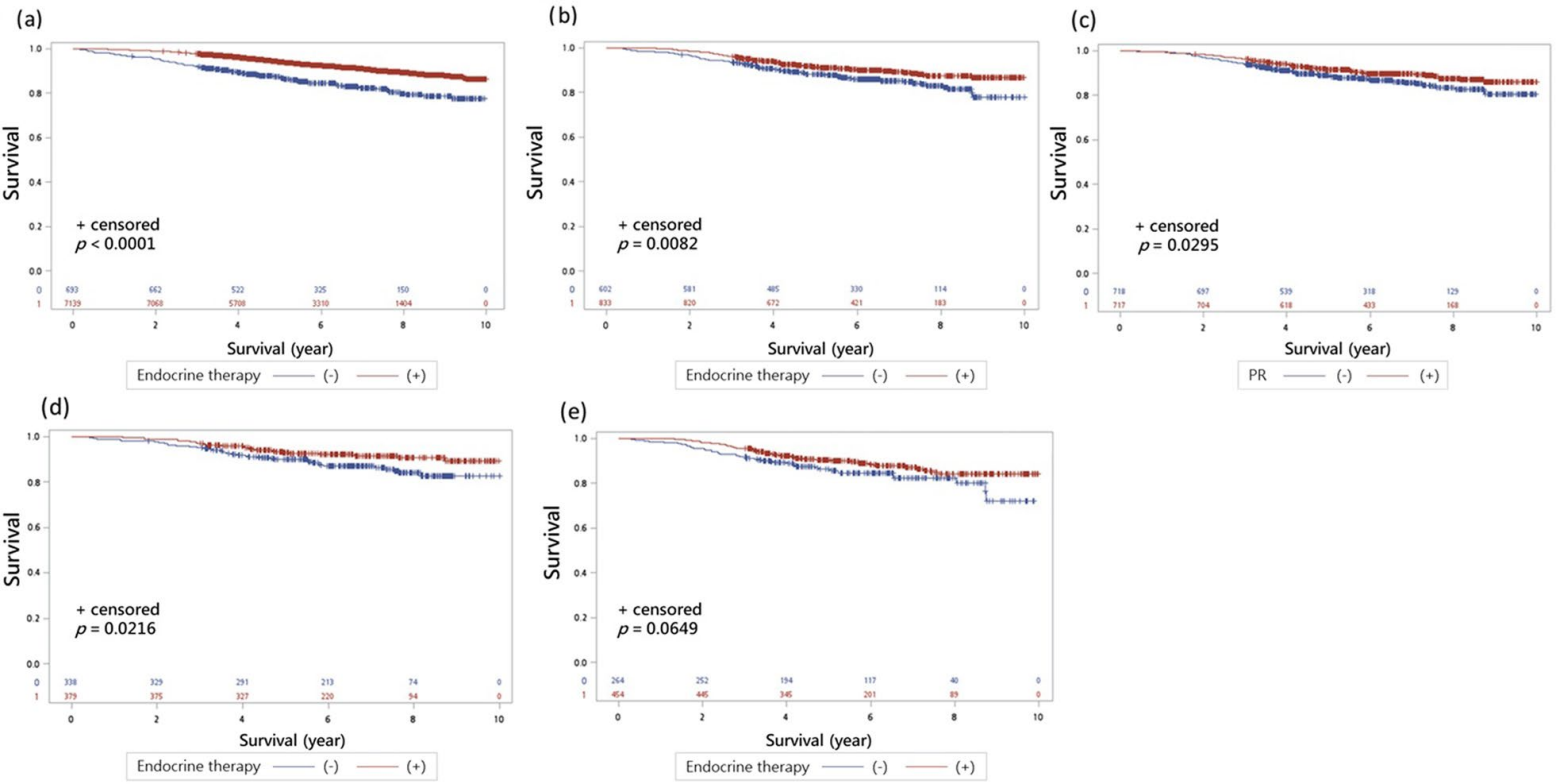
The overall survivals of early breast cancer with HER2-positive. **a** The OS of HER2-positive early breast cacner with ER ≥ 10% group. The OS rate was significantly higher in those with adjuvant ET compared to those without (92.3% vs. 84.0%, *p* < 0.001). **b** The OS of HER2-positive early breast cacner with ER 1–9% group. Similarly, the adjuvant ET group was associated with a statistically better outcome in OS (90.2% vs. 85.6%, *p* = 0.008). **c** The OS of HER2-positive early breast cacner with ER 1–9% group. PR positivity was associated with a significantly better OS (89.5% vs. 86.9%, *p* = 0.030). **d** The OS of HER2-positive early breast cacner with ER 1–9% and PR positivity. Those with PR positivity receiving adjuvant ET had a significantly better OS than those without (92.1% vs. 86.7%, *p* = 0.022). **e** The OS of HER2-positive early breast cacner with ER 1–9% and PR-negative. Among PR-negative and ER 1–9% patients, adjuvant ET showed an insignificant better OS (88.6% vs. 84.1%, *p* = 0.065)

Survival analyses revealed significantly higher overall survival (OS) in patients receiving adjuvant ET across ER groups. In the ER ≥ 10% cohort, OS was 92.3% with ET versus 84.0% without (*p* < 0.001, Fig. 1a). Similarly, ER 1–9% patients showed improved OS with ET (90.2% vs. 85.6%, *p* = 0.008, Fig. 1b).

Within the ER 1–9% group, PR positivity correlated with better OS irrespective of ET (89.5% vs. 86.9%, *p* = 0.030, Fig. 1c). Among PR-positive patients, ET further improved OS (92.1% vs. 86.7%, *p* = 0.022, Fig. 1d). Conversely, in PR-negative ER 1–9% patients, ET showed a non-significant trend toward improved OS (88.6% vs. 84.1%, *p* = 0.065, Fig. 1e). The data of median follow-up time and median survival overall, and the groups stratified by PR analysis were listed in Supplemental Materials (Tables A3, A8, A8a-o).

**Fig. 2:**
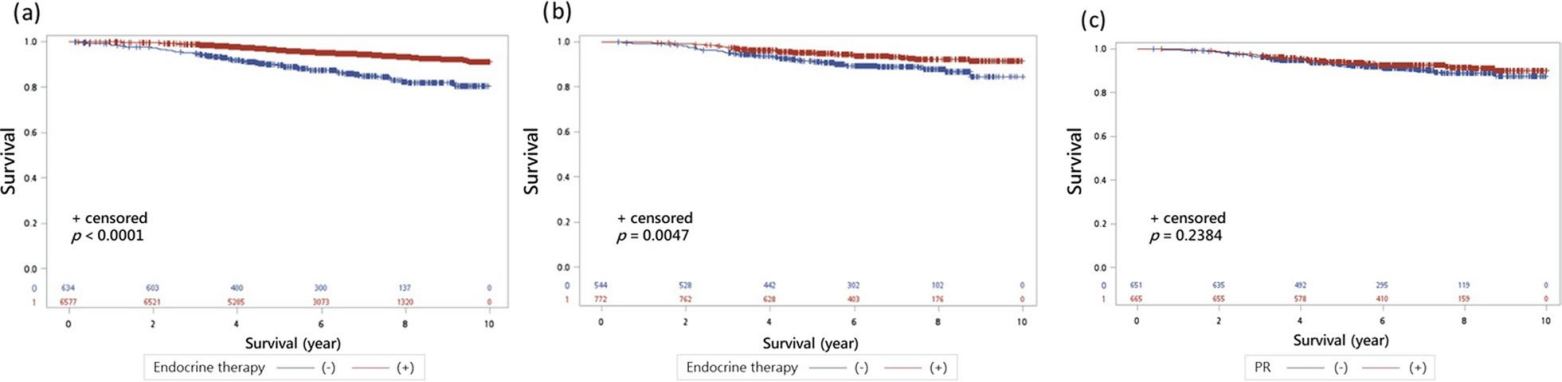
The breast cancer-specific survivals of early breast cancer with HER2-positive. **a** The BCSS of HER2-positive early breast cacner with ER ≥ 10% group. Patients receiving adjuvant ET demonstrated significantly better outcomes compared to those who did not (95.3% vs. 87.1%, *p* < 0.001). **b** The BCSS of HER2-positive early breast cacner with ER 1–9% group. Patients receiving adjuvant ET also demonstrated significantly better outcomes (93.9% vs. 89.7%, *p* = 0.005). **c** The BCSS of HER2-positive early breast cacner with ER 1–9% group comparing PR status. No significant difference in BCSS was observed between dichotomous PR status (92.6% vs. 91.7%, *p* = 0.238)

Breast cancer-specific survival also favored ET recipients in both ER ≥ 10% (95.3% vs. 87.1%, *p* < 0.001, Fig. 2a) and ER 1–9% groups (93.9% vs. 89.7%, *p* = 0.005, Fig. 2b). However, BCSS did not differ significantly by PR status within the ER 1–9% subgroup regardless of ET (92.6% vs. 91.7%, *p* = 0.238, Fig. 2c).

**Fig. 3:**
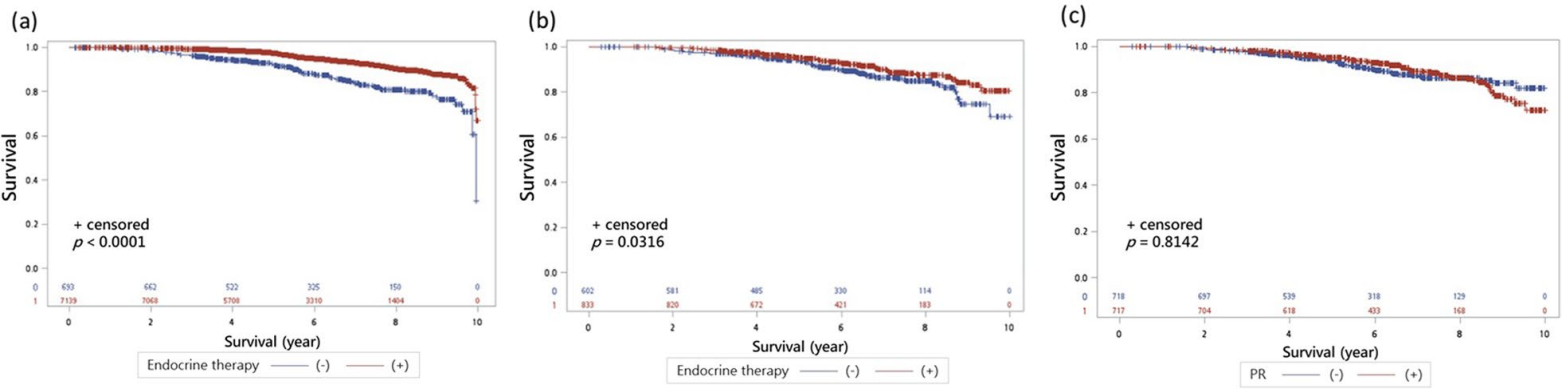
The recurrence-free survivals of early breast cancer with HER2-positive. **a** The RFS of HER2-positive early breast cacner with ER > 10% group. Patients who received adjuvant ET also demonstrated significantly better outcomes than those without (94.2% vs. 86.4%, *p* < 0.001). **b** The RFS of HER2-positive early breast cacner with ER 1–9% group. Patients who received adjuvant ET also demonstrated significantly better outcomes than those without (91.2% vs. 88.2%, *p* = 0.032). **c** The RFS of HER2-positive early breast cacner with ER 1–9% group. No difference in RFS was observed between PR positive and PR negative status (89.3% vs. 90.7%, *p* = 0.814)

Recurrence-free survival was significantly better with ET in both ER ≥ 10% (94.2% vs. 86.4%, *p* < 0.001, Fig. 3a) and ER 1–9% patients (91.2% vs. 88.2%, *p* = 0.032, Fig. 3b). No significant RFS differences were observed between PR-positive and PR-negative ER 1–9% patients irrespective of ET (89.3% vs. 90.7%, *p* = 0.814, Fig. 3c).

Median survival was significantly longer in patients who received adjuvant ET compared to those who did not, across both ER 1–9% and ER ≥ 10% groups. In ER 1–9% patients, ET improved OS, BCSS, and RFS (all *p*-value < 0.050). Importantly, survival benefit increased with longer ET duration (> 60 months), with stepwise improvements in OS, BCSS, and RFS compared to ≤ 24 months or 25–60 months (all *p*-value < 0.001). Subgroup data analysis of ET duration with survival outcomes, were listed in Supplemental Materials (Tables A4, A4a-j).

PR positivity emerged as a significant modifier of ET benefit. In ER 1–9% patients, PR-positive cases demonstrated markedly better OS with ET (92.1% vs. 86.7%, p = 0.022), while PR-negative patients showed only a non-significant trend toward improved OS (88.6% vs. 84.1%, p = 0.065). Further subgroup analysis of PR status and survival outcomes were listed in Supplemental Materials (Tables A8, A8a-o). Multivariate Cox regression confirmed that PR positivity, cancer stage, and receipt of endocrine, chemotherapy, radiotherapy, and targeted therapies were significantly associated with improved OS, BCSS, and RFS. Tumor grade was prognostic for BCSS and RFS. Importantly, low ER positivity was not an independent prognostic factor, whereas PR positivity remained consistently prognostic across outcomes (Table 3).

**Table 3:** Multivariate analysis for OS, BCSS, and RFS by using Cox’s proportional hazards model

		OS Hazard ratio [ 95% CI]	BCSS Hazard ratio [95% CI]	RFS Hazard ratio [95% CI]
Low ER positivity		1.1 [0.9–1.3]	1.0 [0.8–1.3]	1.1 [0.9–1.4]
PR positivity		0.7* [0.6–0.9]	0.8* [0.6–0.9]	0.8* [0.7–0.9]
Grade	Grade 2	1.0 [0.8–1.3]	1.3 [0.9–2.1]	1.9 [1.3–2.9]
	Grade 3	1.1 [0.9–1.5]	1.7 [1.1–2.6]	2.2 [1.5–3.4]
Staging	Stage1	2.6 [1.8–3.9]	3.1 [1.7–5.7]	2.5 [1.6–3.7]
	Stage2	9.0 [6.3–13.0]	11.2 [6.2–20.3]	5.0 [3.3–7.7]
	Stage3*	22.8 [15.7–33.2]	31.2 [17.2–56.7]	12.5 [8.1–19.4]
Operation		2.2 [0.8–5.9]	1.6 [0.5–4.9]	0.7 [0.4–1.5]
Radiotherapy		0.7* [0.6–0.8]	0.8* [0.6–0.9]	0.7* [0.6–0.8]
Chemotherapy		0.4* [0.3–0.4]	0.5* [0.4–0.6]	0.6* [0.5–0.8]
Endocrine therapy		0.6* [0.5–0.7]	0.5* [0.4–0.6]	0.6* [0.5–0.7]
Target therapy		0.8 [0.7–1.0]	0.9 [0.7–1.1]	1.0 [0.9–1.3]

*CI* confidence interval

All multivariable models were adjusted for all covariates listed

*p-value < 0.05

Among the study population, 15.7% received neoadjuvant chemotherapy, while 84.3% underwent adjuvant chemotherapy. Similarly, 11.4% received neoadjuvant endocrine therapy and 88.6% adjuvant endocrine therapy. Targeted therapy was administered in 19.0% of patients in the neoadjuvant setting and 81.0% in the adjuvant setting. The majority of neoadjuvant regimens combined chemotherapy and anti-HER2 therapy (85.9%), while anthracycline- and taxane-based regimens predominated in the adjuvant setting. The subgroup data analyses regarding neoadjuvant/adjuvant treatment fractions with survival outcomes, were listed in Supplemental Materials (Tables A1, A1a-b).

Among 6322 patients receiving chemotherapy, 523 received taxane-only regimens, 2305 anthracycline-only, 17 alkylating-only, 3166 taxane with anthracycline, 70 taxane with alkylating agent, 15 anthracycline with alkylating agent, and 226 triple-combination regimens. These treatment distributions reflected nationwide practice patterns during the study period. The detailed regimen of chemotherapy and duration were listed in Supplemental Materials (Tables A6, A6a).

The overall pCR rate among patients following neoadjuvant chemotherapy was 12.9%. Addition of targeted therapy compared to those without target therapy significantly increased the pCR rate (14.0% vs. 2.5%, *p* < 0.001), whereas the use of neoadjuvant endocrine therapy did not significantly influence pCR rate (12.4% vs. 14.0%, *p* = 0.590) (Supplement Materials Table A2). ER 1–9% tumors were associated with lower pCR rates compared to higher ER expression groups (3.0% vs. 9.7%, *p* = 0.006). Among patients achieving pCR, there was no significant differences in patients with adjuvant ET and without adjuvant ET in OS (*p* = 0.836), BCSS (*p* = 0.138) and RFS (*p* = 0.231). However, for patients without pCR, adjuvant ET resulted in significant better OS (*p* < 0.001), BCSS (*p* < 0.001), and RFS (*p* < 0.001) compared to those without adjuvant ET. The subgroup analysis of pCR rate with survival outcomes, were listed in Supplemental Materials (Tables A2, A2a-j).

In subgroup analysis of ER 1–5% and ER 6–10%, there was no significant difference of OS, BCSS and RFS between subgroup of ER 1–5% (ultra-low) and ER 6–10%. The subgroup analysis of ultra-low ER positivity with survival outcomes, were listed in Supplemental Materials (Tables A9, A9a-b).

In summary, among early HER2-positive breast cancer patients with low ER expression, adjuvant ET significantly improved OS, BCSS, and RFS. Nonetheless, 41.9% of ER 1–9% patients did not receive ET, representing a missed opportunity for survival benefit. Additionally, PR-positive, ER 1–9% patients experienced the greatest OS improvement with ET, highlighting the importance of PR status in guiding therapy decisions. Further subgroup analyses also showed improved OS, BCSS and RFS outcomes of receiving longer ET duration (> 60 months) or for patients without pCR following neoadjuvant therapy.

## Discussion

Recent evidence elucidates the intricate crosstalk between ER and HER2 signaling pathways, supporting dual blockade strategies in breast cancer co-expressing hormone receptors and HER2 [24, 25]. The ExteNET trial further underscores this interaction, demonstrating the clinical benefit of targeting both pathways in HER2- and ER-positive disease [26]. Nonetheless, endocrine therapy carries a substantial burden of adverse effects—such as depression, vasomotor symptoms, vaginal dryness, and arthralgia—which compromise quality of life and contribute to high discontinuation rates (31–73%) before the recommended five-year duration [27, 28]. This raises a critical clinical question: in HER2-positive breast cancer patients with low ER expression (1–9%), can ET be omitted without undermining treatment efficacy?

Our study highlights this clinical uncertainty, showing that 41.9% of Taiwanese patients with HER2-positive early breast cancer and low ER positivity did not receive adjuvant ET. This variability reflects ongoing debate and lack of consensus on ET’s role in this subgroup.

A prospective cohort study by Xie et al. (2022) found improved BCSS with ET in 407 women with low ER-positive disease, particularly among HER2-positive patients [20]. Similarly, Choong et al. reported worse OS with omission of ET in low-ER early breast cancer, independent of PR, HER2, or Ki-67 status [19]. Conversely, Wang, Li et al. observed no significant difference in five-year RFS between ET-treated and untreated patients in a Chinese cohort [11].

Our analysis demonstrates that adjuvant ET significantly improves OS, BCSS, and RFS in HER2-positive early breast cancer with low ER expression. This aligns with ASCO guidelines defining ER positivity at ≥ 1%, and supports using the 1% ER cutoff to guide ET decisions [29]. These findings reinforced the critical role of ER expression in guiding ET decisions and were consistent with existing literature. Importantly, 41.9% of patients with low ER expression in our cohort did not receive ET—indicating a missed opportunity to optimize survival.

Clinicopathologic differences between ER ≥ 10% and ER 1–9% groups were significant (*p* < 0.001), including tumor grade, stage, radiotherapy, and PR positivity (Table 1). The ER-low group had a predominance of grade III tumors (56.1%), while grade II predominated in ER ≥ 10% (54.6%). Stage distribution showed higher proportions of stage 0 and stage III disease in the low ER group. Notably, fewer ER-low patients received adjuvant radiotherapy (51.0% vs. 57.2%). Within the ER-low subgroup, those receiving ET had less advanced disease and higher PR positivity compared to those without ET (Table 2). These differences might result from inherent variations in tumor biology, while selection bias due to sample size disparity could not be totally excluded.

PR status was markedly different between groups: 49.9% PR-positive in ER-low versus 81.6% in ER ≥ 10% tumors. Within the ER-low cohort, PR positivity was significantly higher in ET recipients (*p* < 0.001), highlighting PR’s potential predictive role beyond ER levels.

Previous studies have demonstrated that PR signaling exerts a critical modulatory effect on estrogen receptor alpha (ERα) activity within the breast cancer microenvironment, influencing both tumor biology and therapeutic responsiveness [30–33]. Prior research suggests PR expression enhances response to adjuvant ET, while low PR may contribute to tamoxifen resistance [34–37]. In our cohort, PR-positive, ER-low patients receiving ET had significantly better OS (92.1% vs. 86.7%, *p* = 0.022), whereas PR-negative patients showed a non-significant trend favoring ET (88.6% vs. 84.1%, *p* = 0.065). No significant differences were observed in BCSS or RFS by PR status within this subgroup.

These findings echo those of Bardou et al., who identified PR positivity as an independent predictor of ET benefit, with concordant ER/PR-positive tumors deriving superior survival improvements compared to discordant cases [38]. Lashen et al. further proposed a 10% PR positivity cutoff to refine prognostication [39].

Multivariate analysis in our study revealed that low ER expression was not an independent prognostic factor for OS, BCSS, or RFS, whereas PR positivity consistently predicted improved outcomes. Other significant prognostic factors included tumor stage, radiotherapy, chemotherapy, and ET receipt. These results parallel the meta-analysis by Boland et al., encompassing 13,667 patients, which linked PR negativity with poorer OS and disease-free survival [40].

Our expanded analyses clarify several important aspects of treatment response in HER2-positive, ER-low breast cancer. First, the relatively modest pCR rates with chemotherapy alone underscore the aggressive biology of HER2-positive tumor, while the significant benefit from combined chemotherapy and targeted therapy highlights the importance of anti-HER2 treatment. Second, our findings reinforce that ET confers measurable survival advantages in ER-low patients, particularly when administered for a longer duration or non-pCR patients. The dose–responsive relationship between ET duration and survival suggests adherence and persistence are critical to maximizing benefit, even in tumors with low ER expression.

Importantly, PR positivity emerged as a key determinant of ET benefit, consistent with prior reports linking PR expression to improved endocrine responsiveness. This observation suggests that PR may serve as a more reliable biomarker than ER percentage alone in guiding ET use among HER2-positive, ER-low patients. Conversely, the limited effect of ET in PR-negative patients implies the need for alternative therapeutic strategies in this subgroup.

Dissection of chemotherapy regimen demonstrated that most patients received taxane and anthracycline combinational regiments, reflecting guideline-concordant practice. The lack of chronological survival improvement in ER-low patients over the study period indicates that advances in HER2-targeted therapy and chemotherapy may not fully compensate for endocrine resistance, underscoring the critical impact of optimizing ET strategies.

Collectively, these findings further support the importance of PR status as a potential biomarker in evaluating ET benefit; however, additional data are needed before incorporating PR status and ET duration into routine clinical practice.

In summary, adjuvant ET confers survival benefits in HER2-positive early breast cancer irrespective of ER expression level. The substantial proportion (41.9%) of ER-low patients in Taiwan not receiving ET represents a critical gap in care that should be addressed through clinician-led counseling to optimize treatment uptake. PR positivity may serve as a key biomarker to identify ER-low patients most likely to benefit from ET, supporting its integration into therapeutic decision-making. Subgroup analyses also showed improved OS, BCSS and RFS from receiving longer ET duration (> 60 months) or for non-pCR patients following neoadjuvant therapy.

While the survival benefit of ET seemed more evident among patients without pCR, the small number of pCR cases limited our ability to assess the effect in this subgroup. Therefore, ET should not be omitted in patients who achieve pCR, and treatment decisions should continue to follow current clinical guidelines.

Limitations of this study included the retrospective study design, which inherently introduced selection bias. The relatively smaller sample size of ER-low patients further compromised statistical power. Due to the limitation of the TCR, we only can traced drugs that were reimbursed by National Health Insurance. Thus, self-paid medications such as pertuzumab were not recorded.

In addition, a proportion of patients in our cohort received chemotherapy without HER2-targeted therapy, accounting for more than one-fourth (2147 patients, 28.1%). Consistent with prior real-world experience, the likelihood of receiving chemotherapy alone was higher in the earlier years of the study period, when trastuzumab was limitedly reimbursed. In contrast, after 2015 the proportion of patients treated without targeted therapy markedly decreased, reflecting broader clinical uptake and enhanced guideline concordance. These observations are in line with published real-world studies showing that, even after trastuzumab approval, 10–30% of patients with early-stage HER2-positive breast cancer received chemotherapy alone without targeted therapy, particularly in earlier years or among elderly/comorbid patients [41, 42]. A systematic review had similarly reported trastuzumab uptake rates of ~ 70%, indicating that nearly one-third of eligible patients did not receive targeted therapy in certain settings [42]. Other limitations including lack of certain treatment details such as drug doses, and absence of molecular subtyping data.

## Conclusions

This real-world data from Taiwan provide valuable insight for the use of adjuvant ET among patients with HER2-positive early breast cancer and ER-low status. Despite demonstrating survival benefits, approximately 41.9% of patients with ER expression of 1–9% did not receive adjuvant ET. These findings highlighted the need to actively consider and discuss ET for candidate patients to enhance treatment outcomes. Further subgroup analysis also showed improved OS, BCSS and RFS resulted from longer ET duration (> 60 months) or for non-pCR patients. This study demonstrates real-world evidence of survival benefit to support clinicians and patients in making more informed decisions regarding adjuvant ET for early-stage ER-positive, HER2-positive breast cancer through shared decision-making.

### Implications for practice

Adjuvant endocrine therapy (ET) improves survival in HER2-positive early breast cancer patients with low ER expression, especially when PR is positive. PR status could be considered a key biomarker to guide ET decisions in this subgroup. There were over 40% of eligible patients do not receive ET in Taiwan, indicating a treatment gap. Clinicians could incorporate PR assessment and consider ET in ER-low, HER2-positive patients to optimize individualized care and improve outcomes. Receiving adjuvant ET also showed survival benefits in non-pCR group. Longer ET duration (> 60 months) presented with betted survival outcomes. Adjuvant ET may be beneficial and warrant further confirmation in prospective studies.

## Supplementary Information

Supplementary file1.

## Data Availability

Data for this study were obtained from the Health and Welfare Data Science Center, Ministry of Health and Welfare, Taiwan ([URL:// https://dep.mohw.gov.tw/DOS/cp-5119–59201-113.html]). All data generated or analyzed during this study are included in this published article and its supplementary information files.

## Abbreviations

ER: Estrogen receptor
PR: Progesterone-receptor
HER2: Human epidermal growth factor receptor 2
FISH: Fluorescence in situ hybridization
ET: Endocrine therapy
OS: Overall survival
BCSS: Breast cancer-specific survival
RFS: Recurrence-free survival
