# Supplementary material for: Survival analysis of adjuvant endocrine therapy in HER2 positive early breast cancer patients with low ER positivity
Source: Breast Cancer Res. 2025 Dec 29;27:222. doi: 10.1186/s13058-025-02157-9 (PMC12751189; doi:10.1186/s13058-025-02157-9)
Supplement: Supplementary file 1 — Supplementary file1. [file 13058_2025_2157_MOESM1_ESM.docx]

## Supplemental Materials

## Table A1. Distribution of systemic treatments

| Treatment type | Percentage (%) |
| --- | --- |
| Neoadjuvant chemotherapy | 15.71 |
| Adjuvant chemotherapy | 84.29 |
| Neoadjuvant endocrine therapy | 11.44 |
| Adjuvant endocrine therapy | 88.56 |
| Neoadjuvant targeted therapy | 18.98 |
| Adjuvant targeted therapy | 81.02 |

*Neoadjuvant treatment:

- Neoadjuvant chemotherapy: 12.44%
- Neoadjuvant chemotherapy + target therapy: 85.87%
- Neoadjuvant target therapy: 1.69%

*Adjuvant treatment:

- Adjuvant chemotherapy: 30.96%
- Adjuvant chemotherapy + target therapy: 66.50%
- Adjuvant target therapy: 2.54%


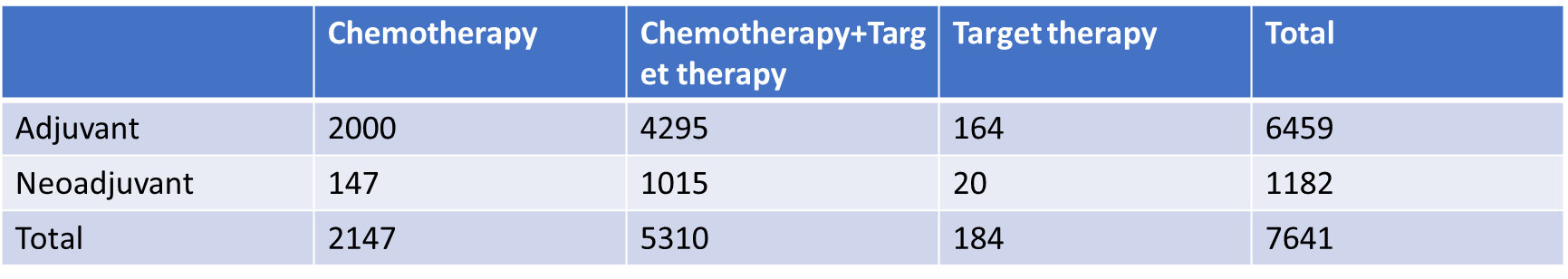


## Table A1a. Neoadjuvant treatment and response


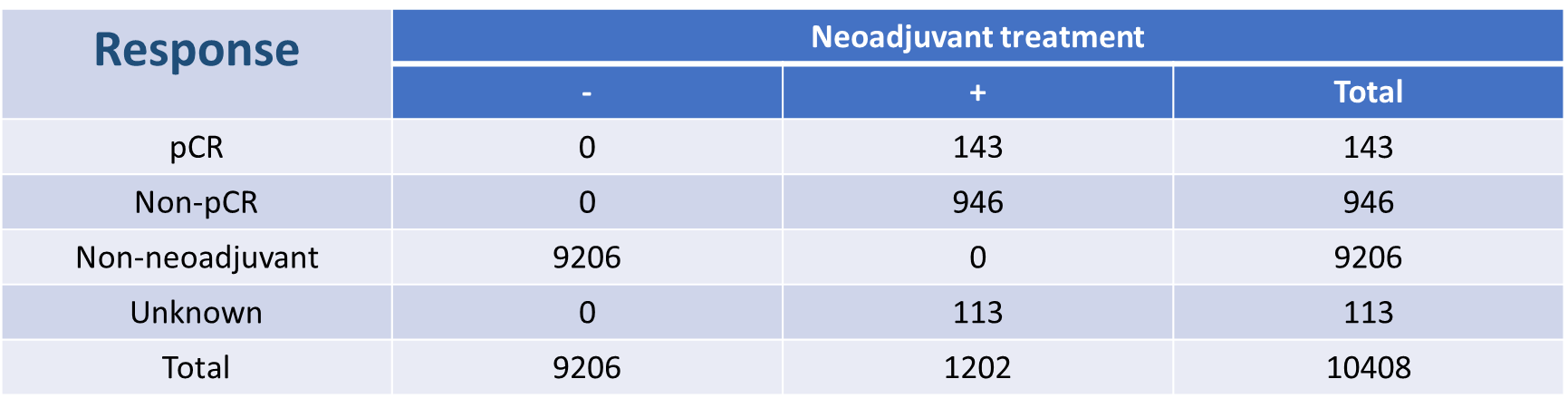


## Table A1b. Neoadjuvant treatment and ER low


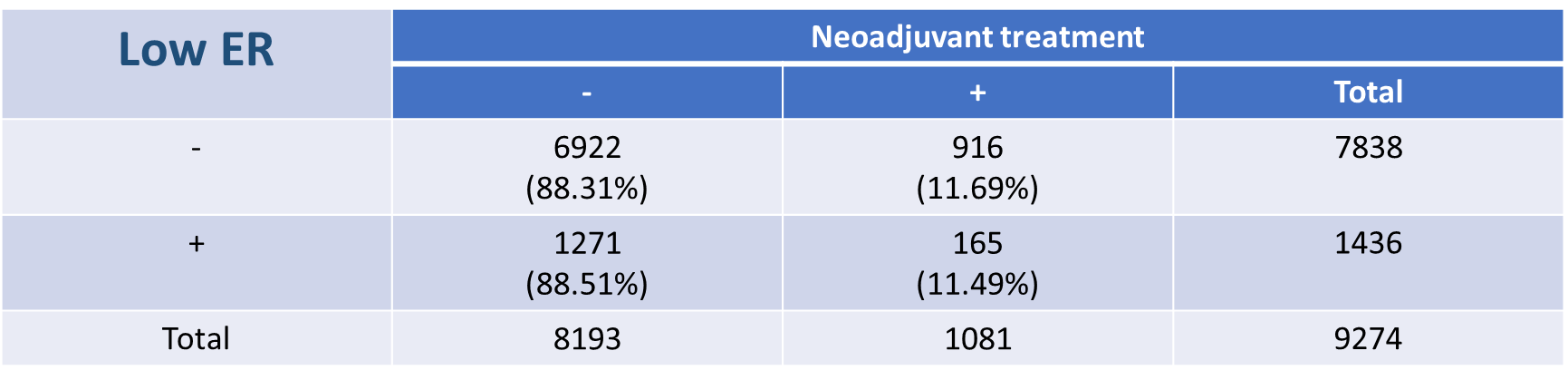


## Table A2. Pathological complete response (pCR) rates and treatment associations

| Comparison | Result / Rate | P-value |
| --- | --- | --- |
| pCR and ER-low status (+)  pCR and ER-low status (-) | 29 cases/19.59%  95 cases/11.42% | 0.0058 |
| pCR rate with neoadjuvant chemotherapy | 12.85% |  |
| pCR rate with neoadjuvant target therapy  pCR rate without neoadjuvant target therapy | 14.04%  2.54% | 0.0004 |
| pCR rate with neoadjuvant endocrine therapy  pCR rate without neoadjuvant endocrine therapy | 12.41%  14.00% | 0.5898 |

## Table A2a. pCR rate and low ER


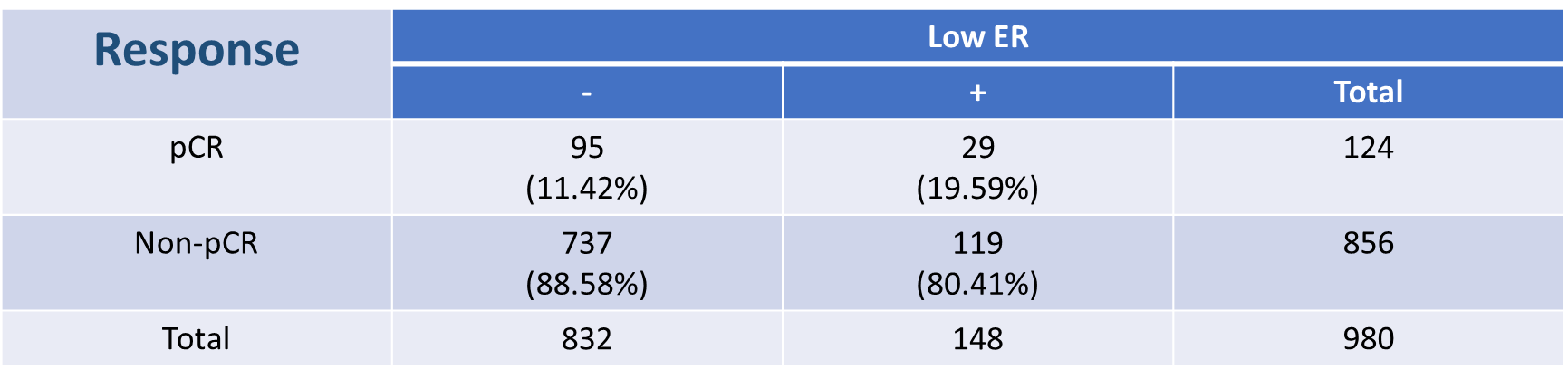


## Table A2b. pCR rate and neoadjuvant target therapy


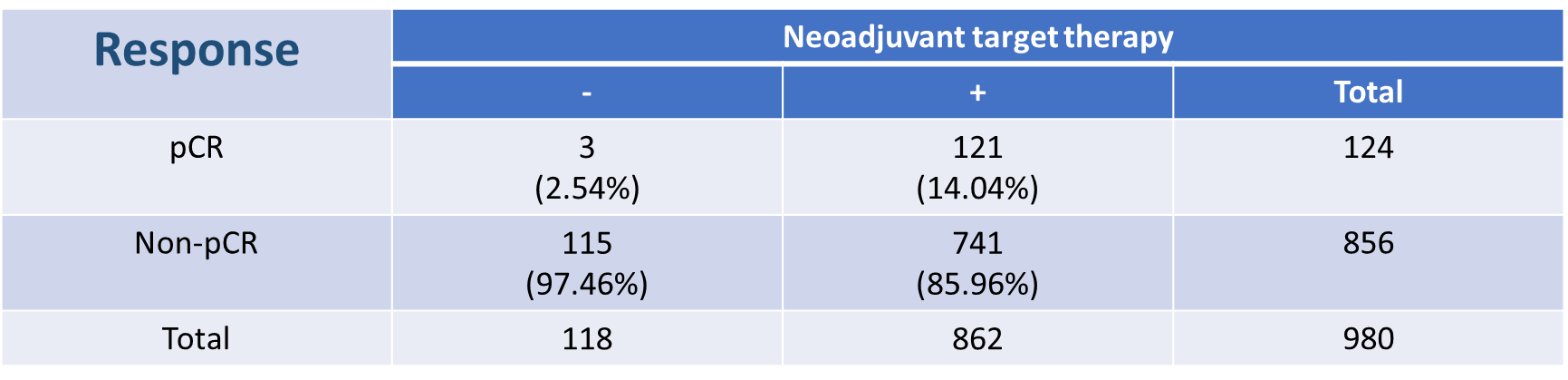


## Table A2c. pCR rate and neoadjuvant endocrine therapy


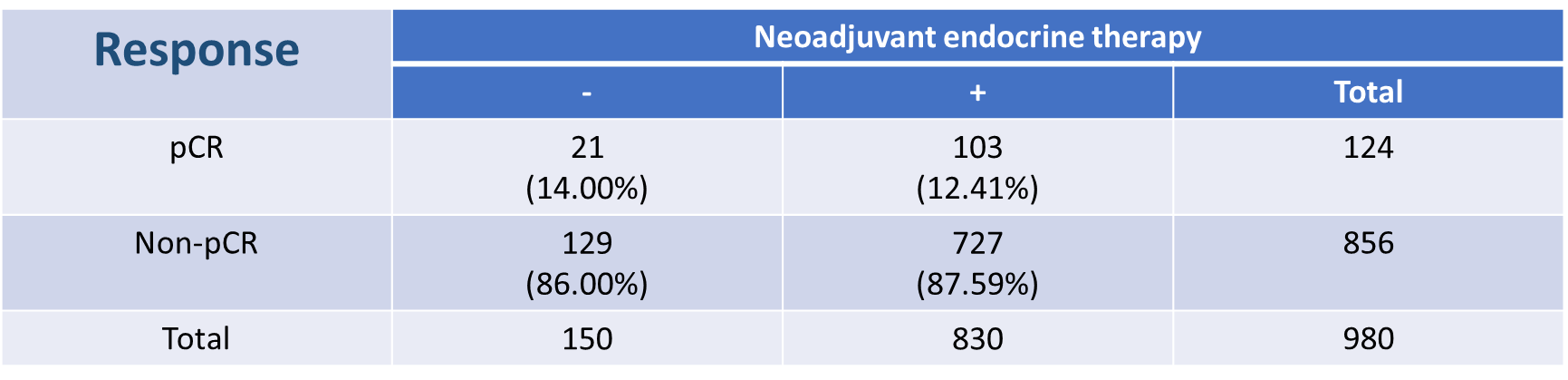


## Table A2d. pCR rate and neoadjuvant treatment


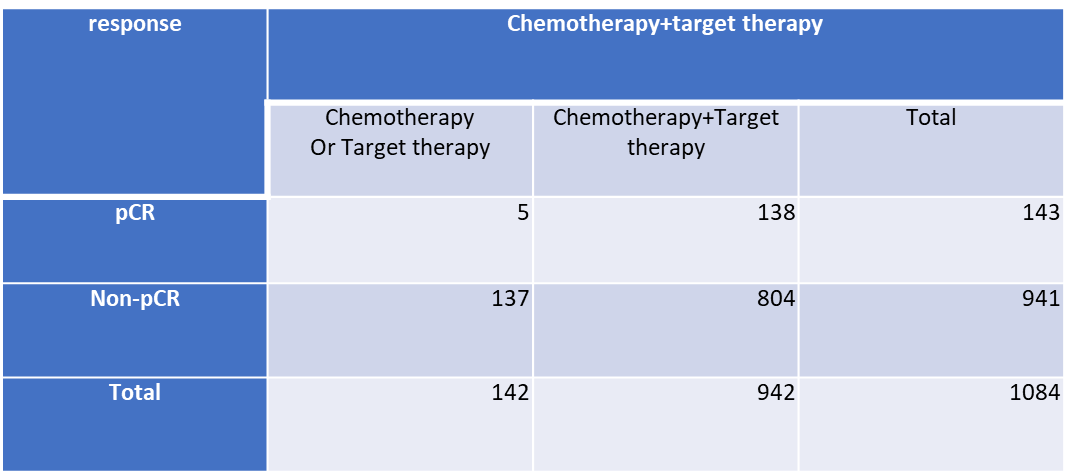


## Table A2e. The overall survival with pCR(+) and endocrine treatment and cox-regression model analysis


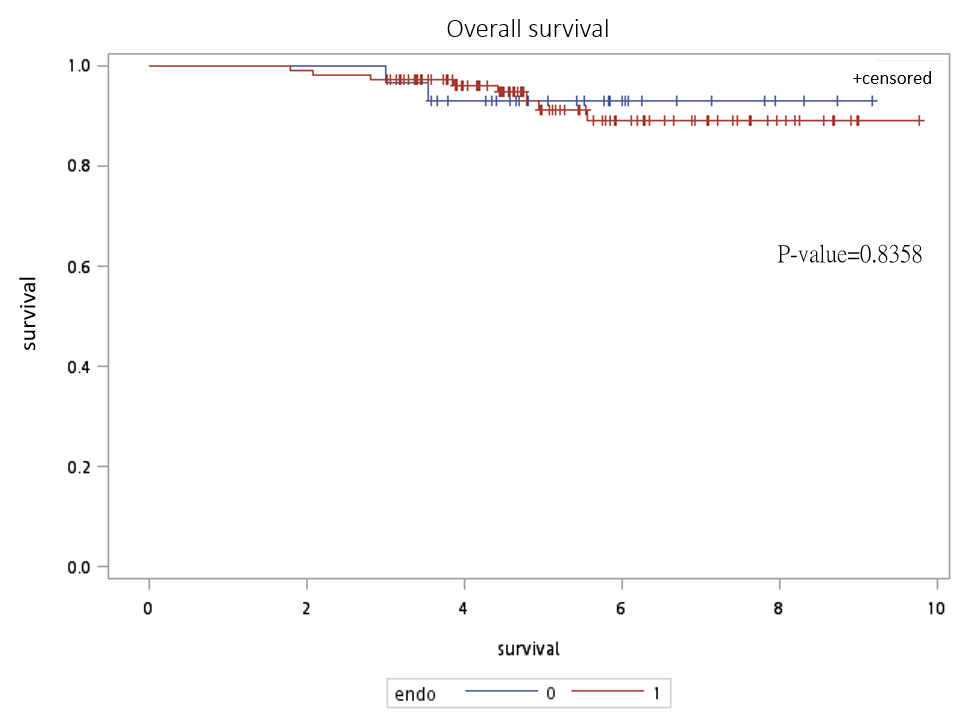

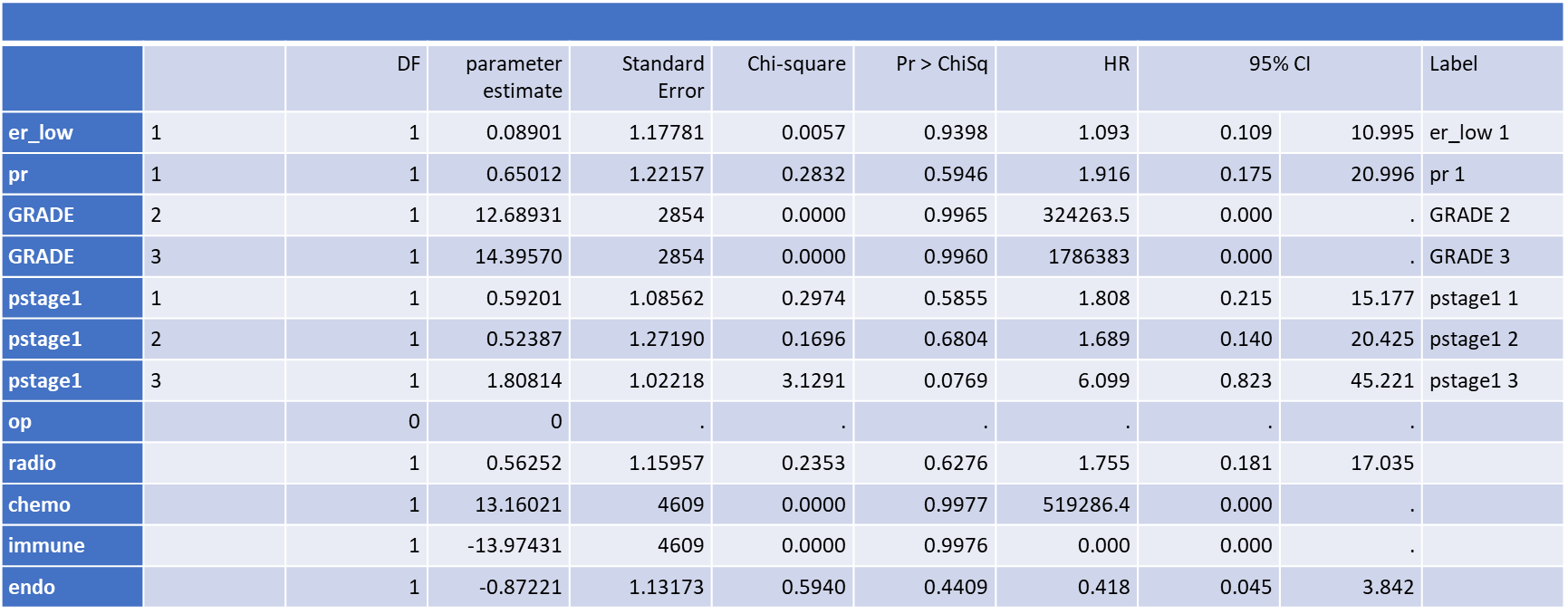


*DF= Degrees of Freedom, Pr>ChiSq=p-value, HR=Hazard ratio, CI=Confidence interval

## Table A2f. The breast cancer specific survival with pCR(+) and endocrine treatment and cox-regression model analysis


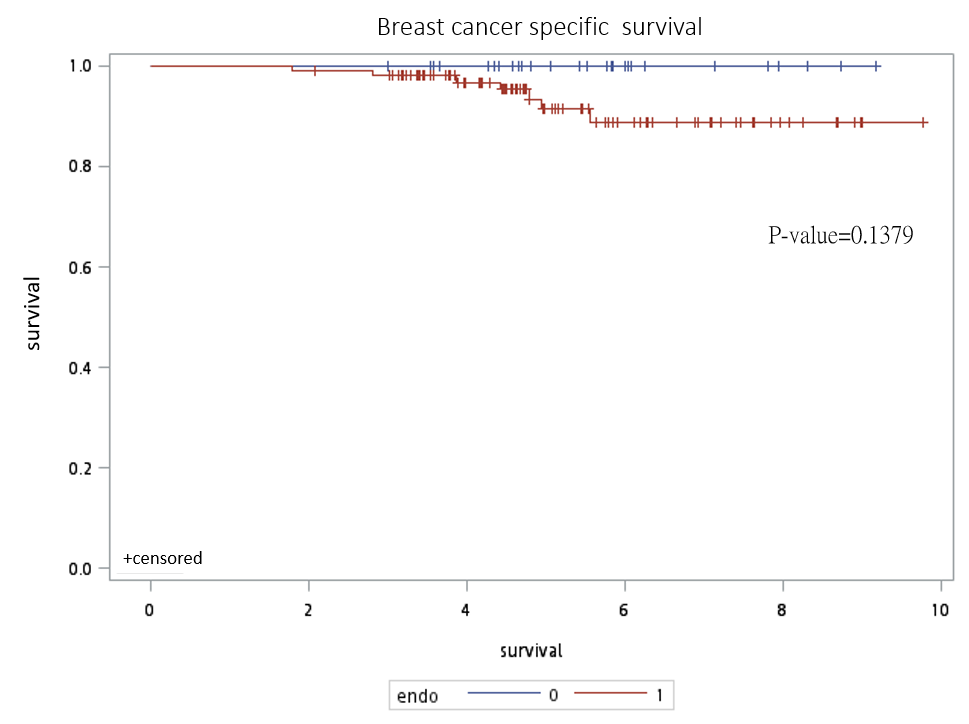


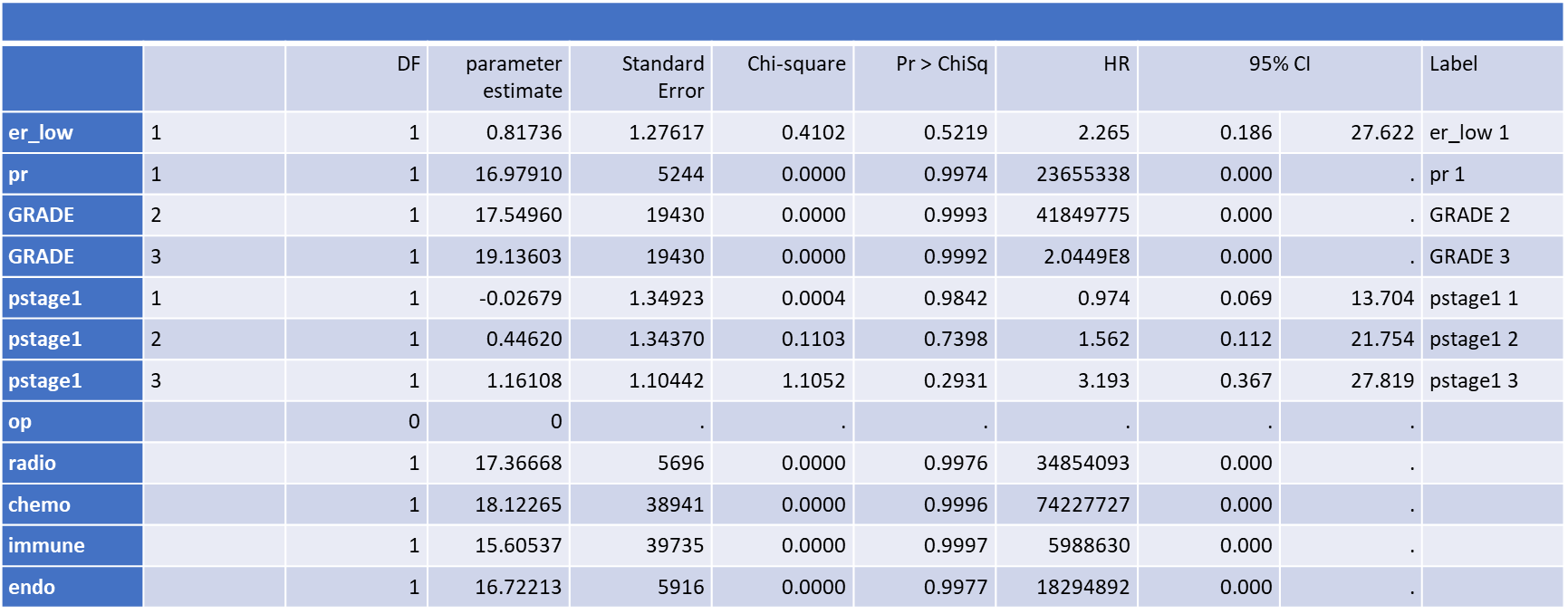


*DF= Degrees of Freedom, Pr>ChiSq=p-value, HR=Hazard ratio, CI=Confidence interval

## Table A2g. The recurrence survival with pCR(+) and endocrine treatment and cox-regression model analysis


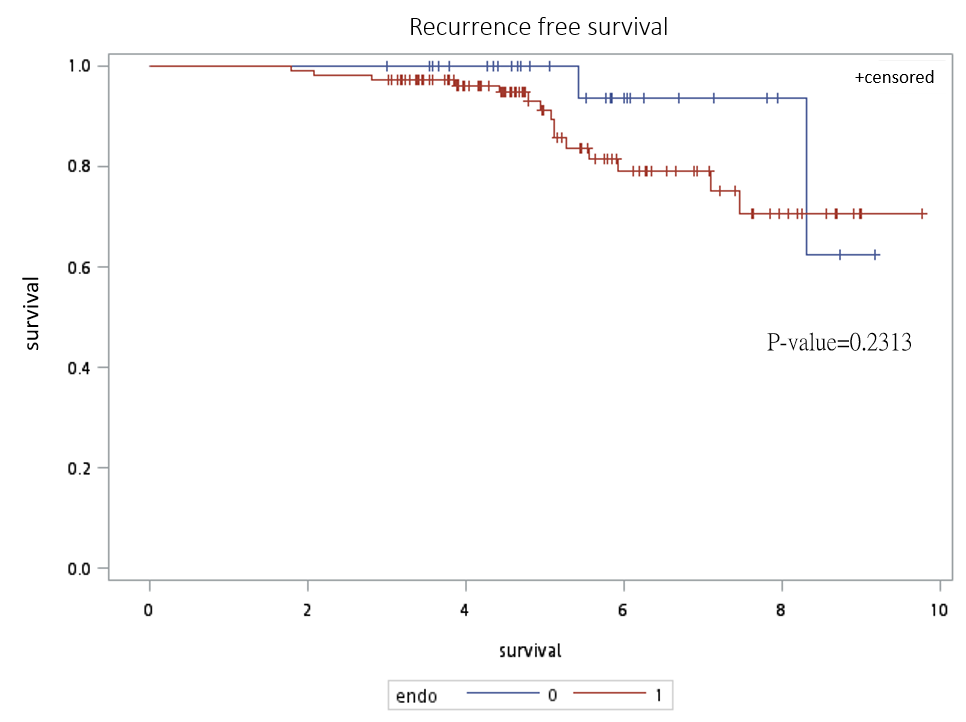


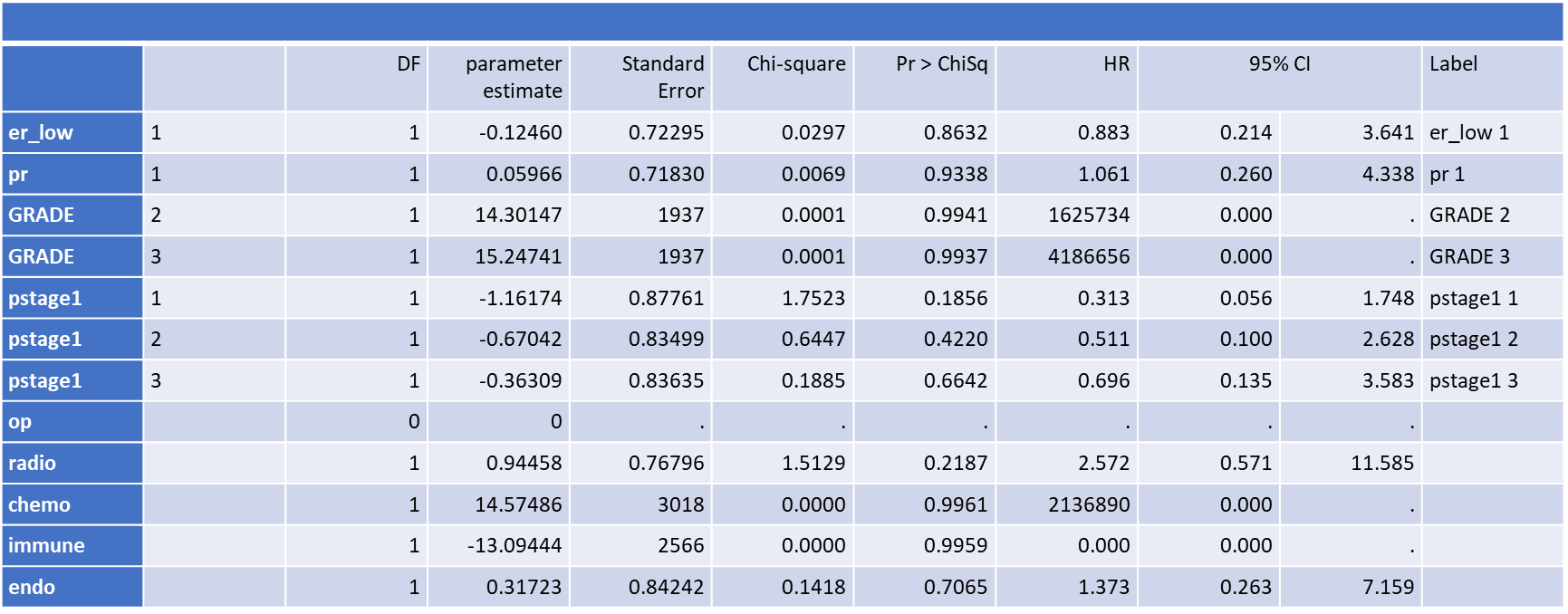


*DF= Degrees of Freedom, Pr>ChiSq=p-value, HR=Hazard ratio, CI=Confidence interval

## Table A2h. The overall survival with non-pCR and endocrine treatment and cox-regression model analysis


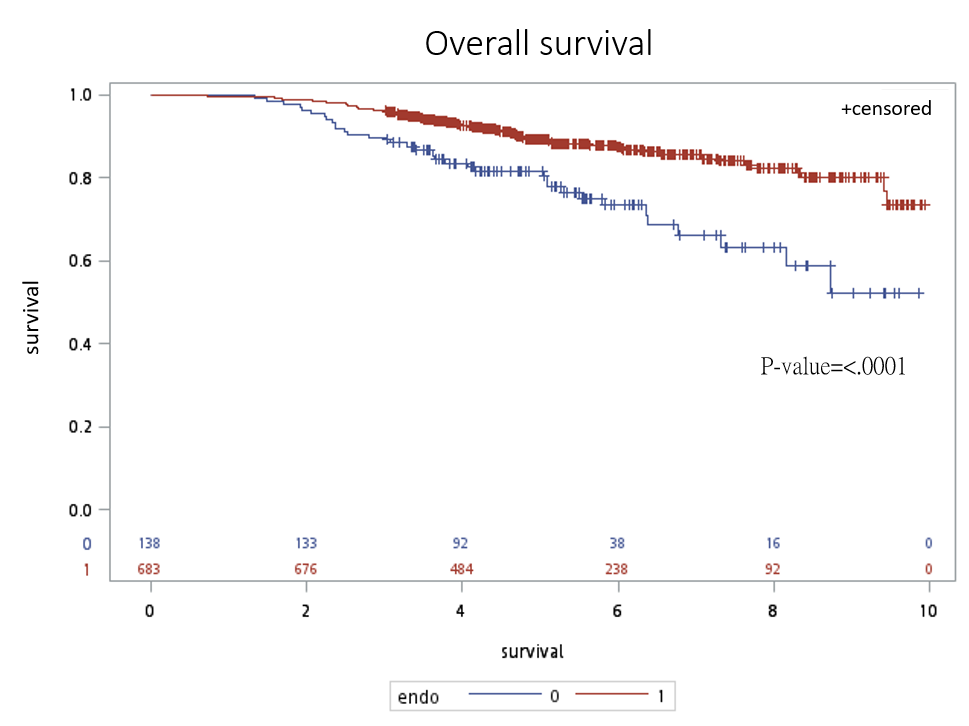


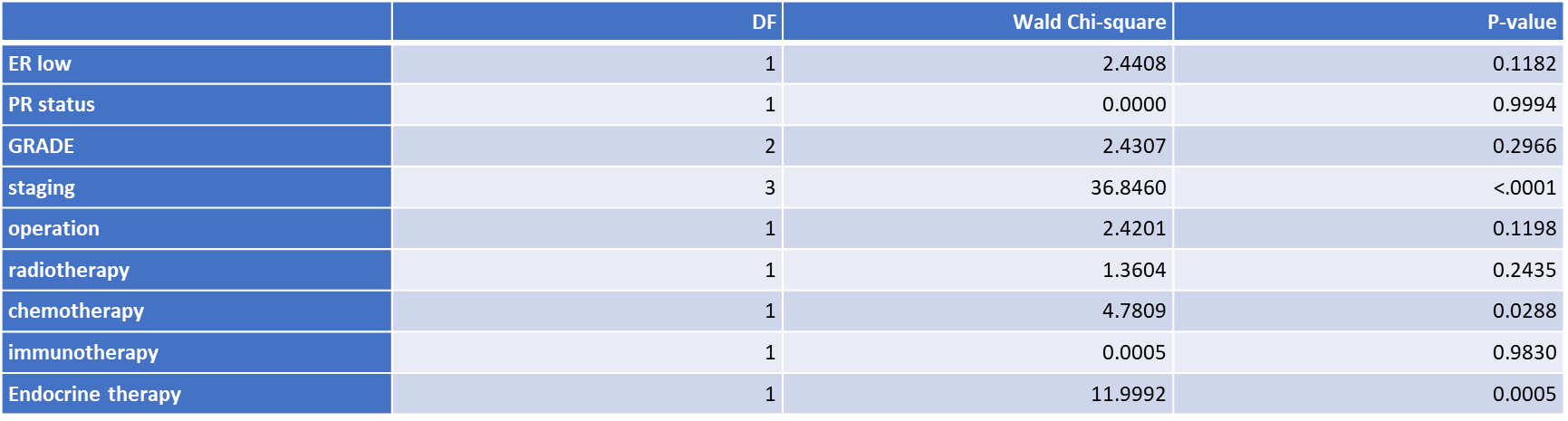


*DF= Degrees of Freedom


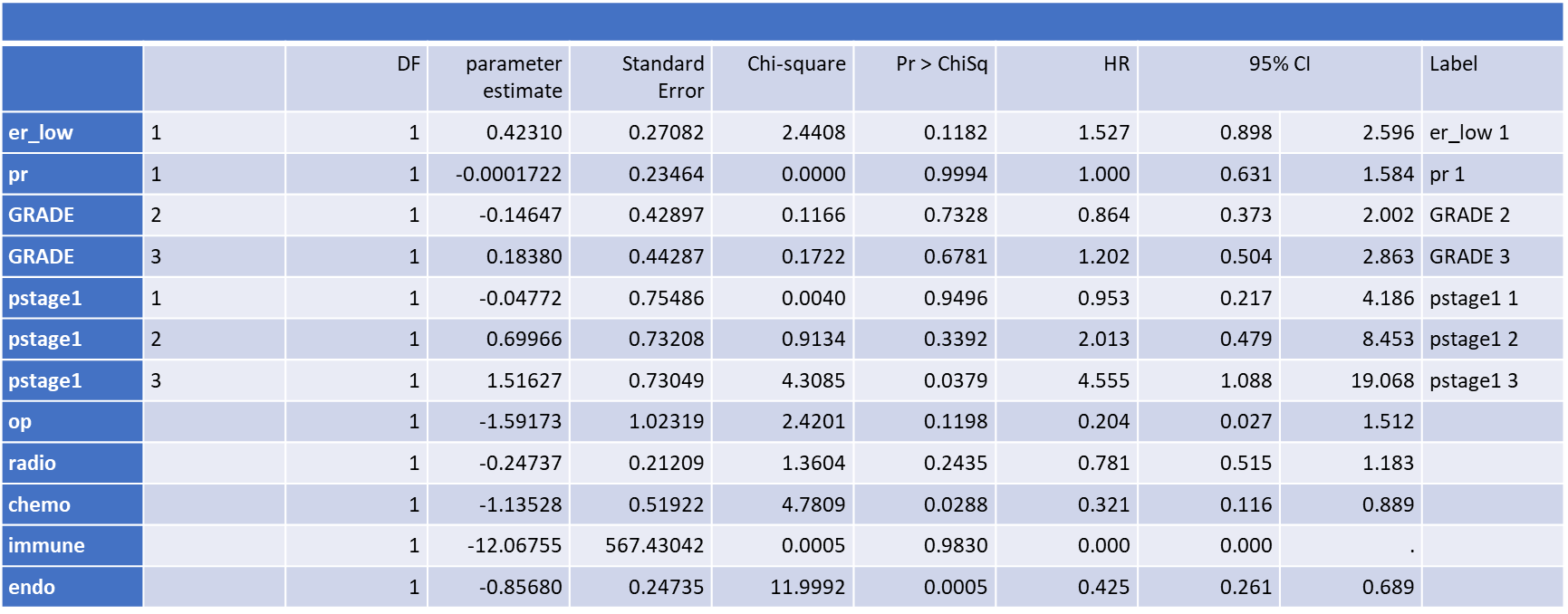


*DF= Degrees of Freedom, Pr>ChiSq=p-value, HR=Hazard ratio, CI=Confidence interval

## Table A2i. The breast cancer survival with non-pCR and endocrine treatment and cox-regression model analysis


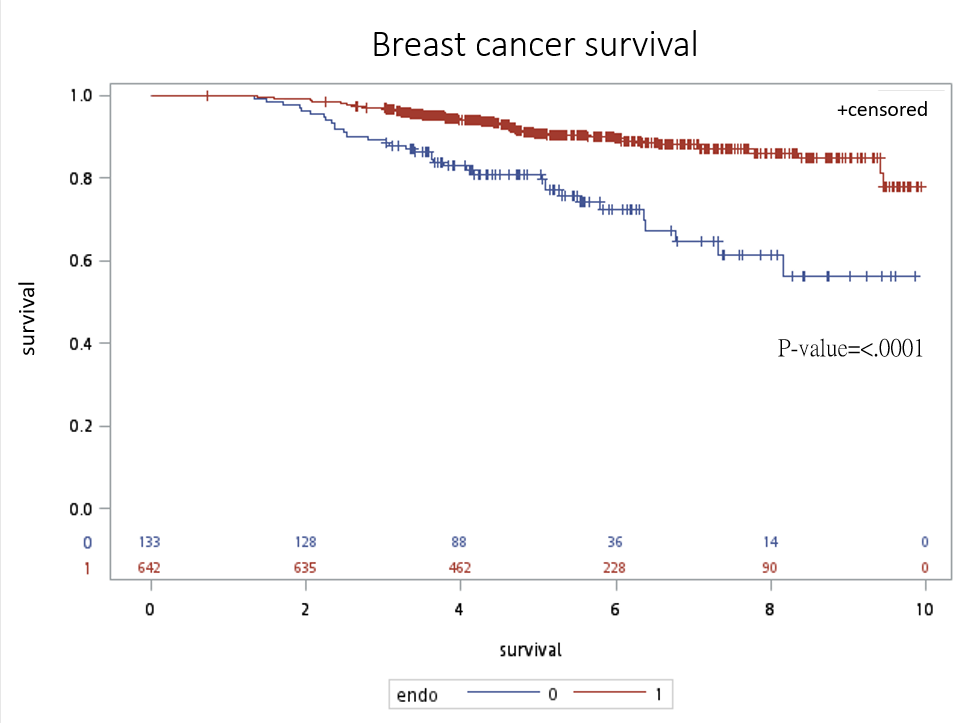

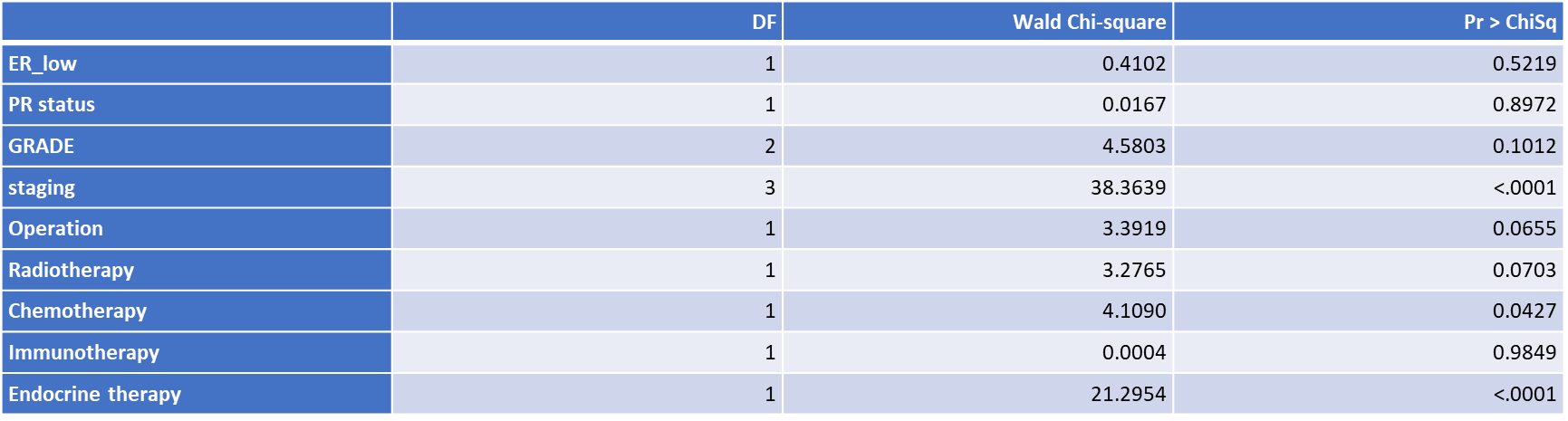


*DF= Degrees of Freedom, Pr>ChiSq=p-value


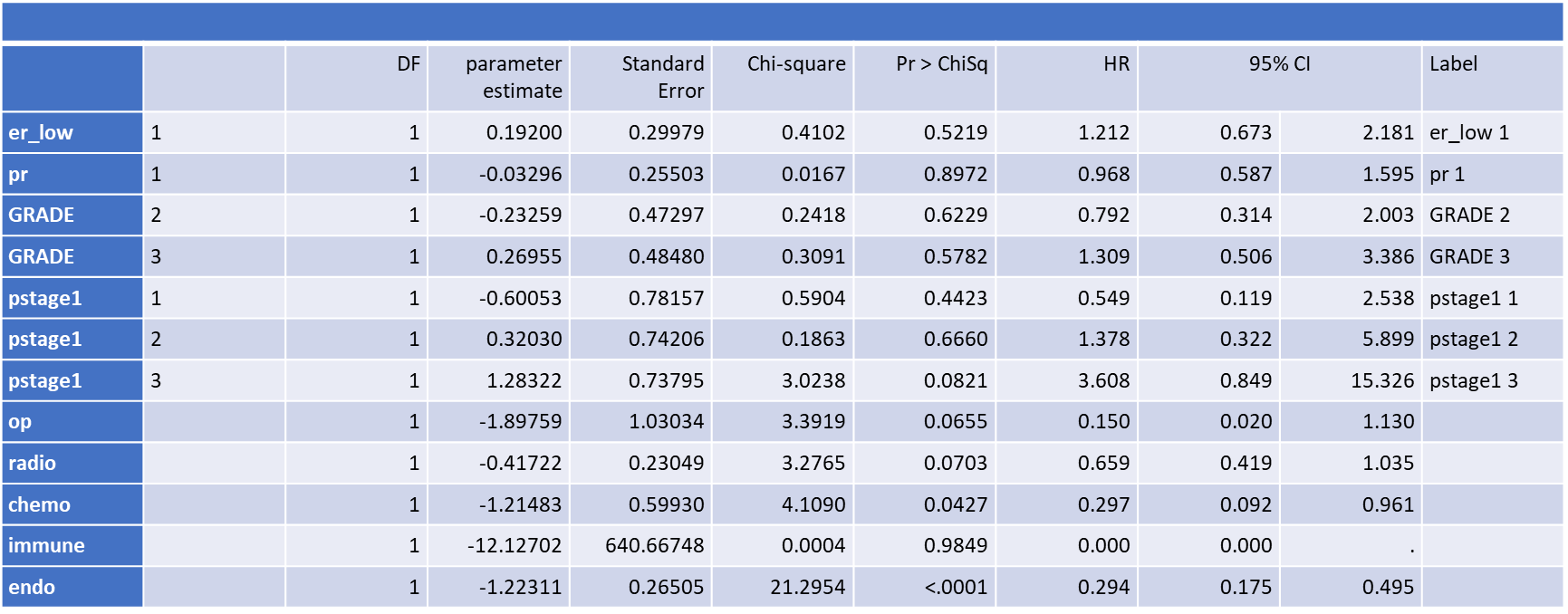


*DF= Degrees of Freedom, Pr>ChiSq=p-value, HR=Hazard ratio, CI=Confidence interval

## Table A2j. The recurrence free survival with non-pCR and endocrine treatment and cox-regression model analysis


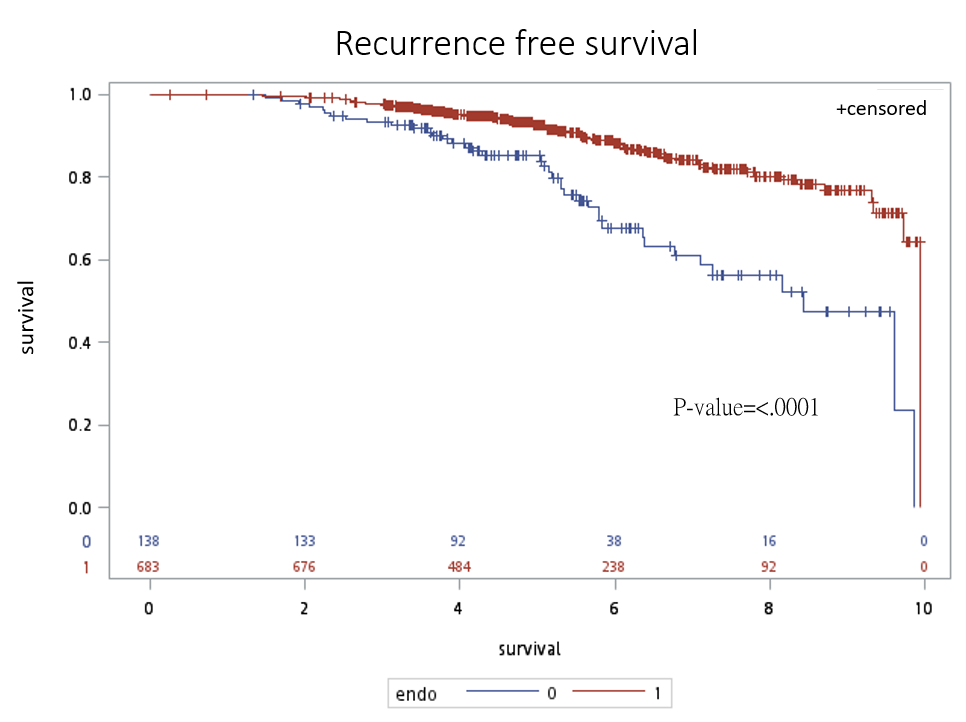

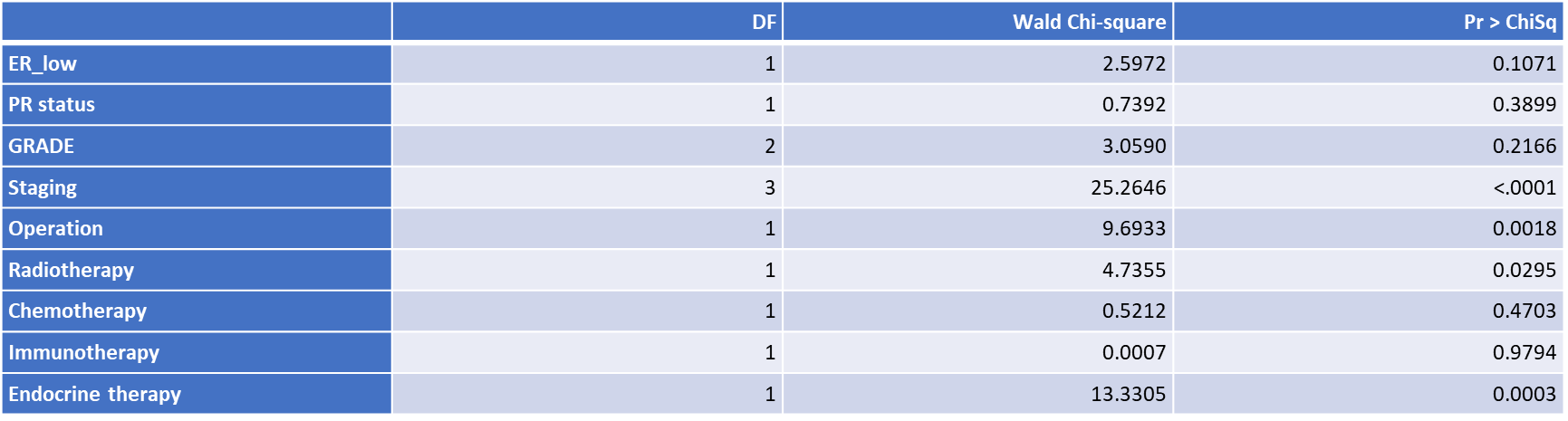


*DF= Degrees of Freedom, Pr>ChiSq=p-value


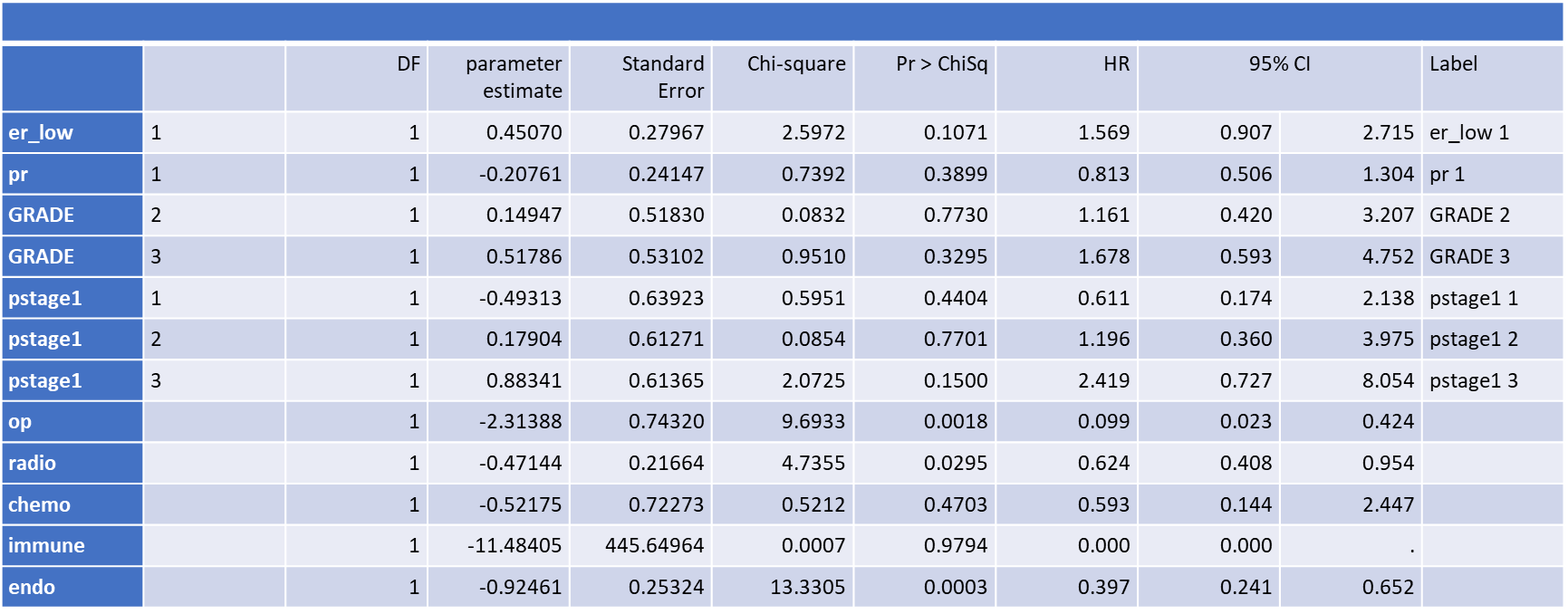


*DF= Degrees of Freedom, Pr>ChiSq=p-value, HR=Hazard ratio, CI=Confidence interval

## Table A3. Survival analysis by ER status

| Subgroup | OS (P-value) | BCSS (P-value) | RFS (P-value) |
| --- | --- | --- | --- |
| ER-low vs. ER-high | 0.8358 | 0.1379 | 0.2313 |
| Non-pCR patients | <0.0001 | <0.0001 | <0.0001 |

Follow-up cut-off date: December 31, 2022; maximum survival time: 10 years.

* Survival time (years) of follow-up to 10 years


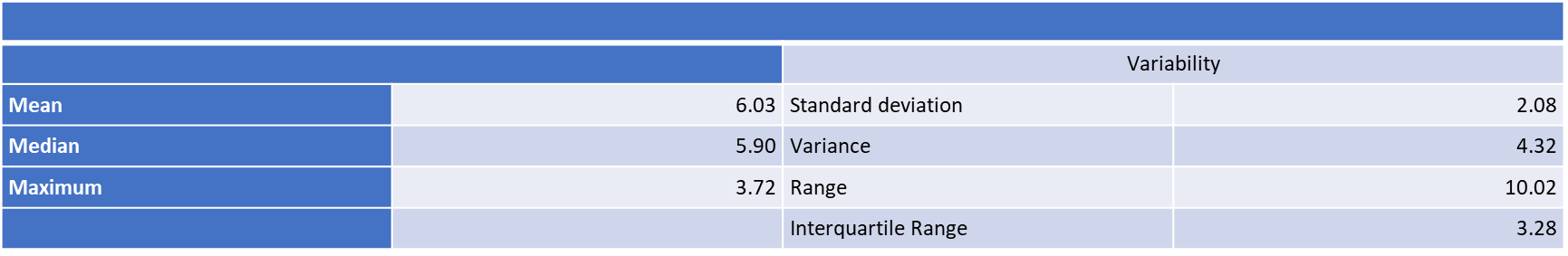


* Survival time (years) of follow-up to 10 years in subgroup of patients without endocrine therapy


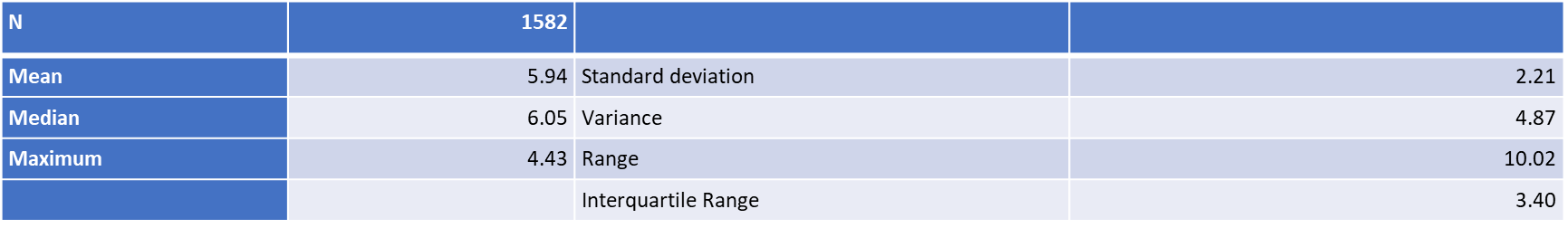


* Survival time (years) of follow-up to 10 years in subgroup of patients with endocrine therapy


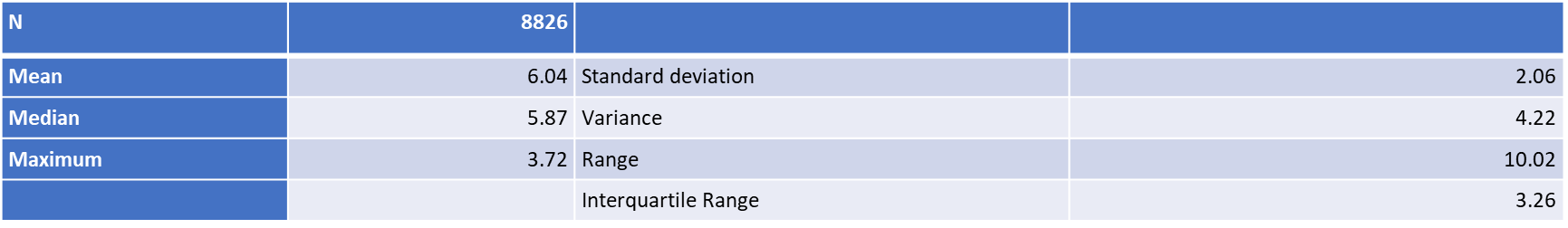


* Mean overall survival time (years) in subgroup of patients with or without endocrine therapy

| ER 1-9% | Mean | Standard deviation |
| --- | --- | --- |
| Without endocrine therapy | 7.92 | 0.09 |
| With endocrine therapy | 8.17 | 0.06 |
| ER >=10% | Mean | Standard deviation |
| Without endocrine therapy | 8.10 | 0.09 |
| With endocrine therapy | 8.98 | 0.02 |

* Mean breast cancer specific survival time (years) in subgroup of patients with or without endocrine therapy

| ER 1-9% | Mean | Standard deviation |
| --- | --- | --- |
| Without endocrine therapy | 8.15 | 0.08 |
| With endocrine therapy | 8.39 | 0.05 |
| ER >=10% | Mean | Standard deviation |
| Without endocrine therapy | 8.32 | 0.09 |
| With endocrine therapy | 9.18 | 0.02 |

* Mean recurrence free survival time (years) in subgroup of patients with low ER positivity with or without endocrine therapy

| ER 1-9% | Mean | Standard deviation |
| --- | --- | --- |
| Without endocrine therapy | 8.75 | 0.09 |
| With endocrine therapy | 8.80 | 0.06 |
| ER >=10% | Mean | Standard deviation |
| Without endocrine therapy | 8.93 | 0.10 |
| With endocrine therapy | 9.50 | 0.02 |

## Table A3a. The overall survival analysis by cox regression model with neoadjuvant treatment response


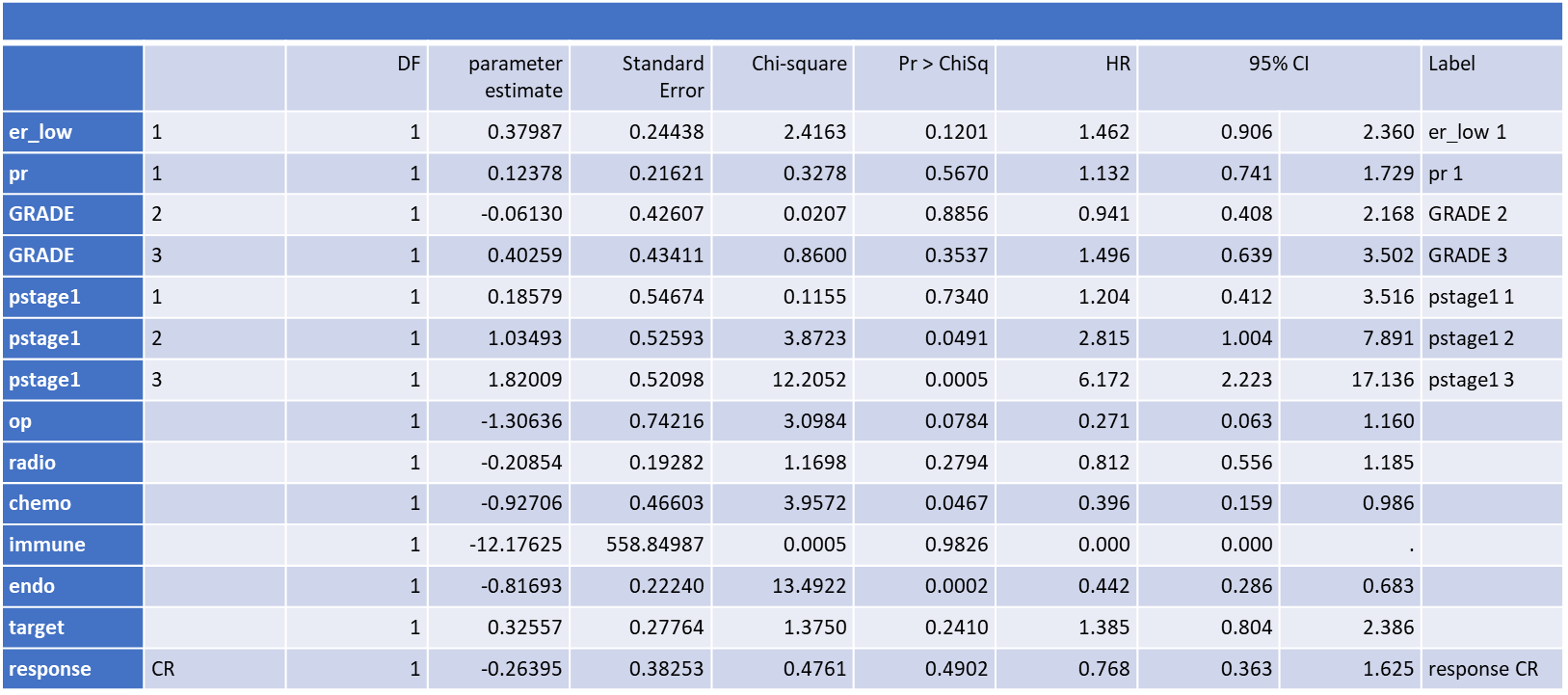


*DF= Degrees of Freedom, Pr>ChiSq=p-value, HR=Hazard ratio, CI=Confidence interval

## Table A3b. The breast cancer specific survival analysis by cox regression model with neoadjuvant treatment response


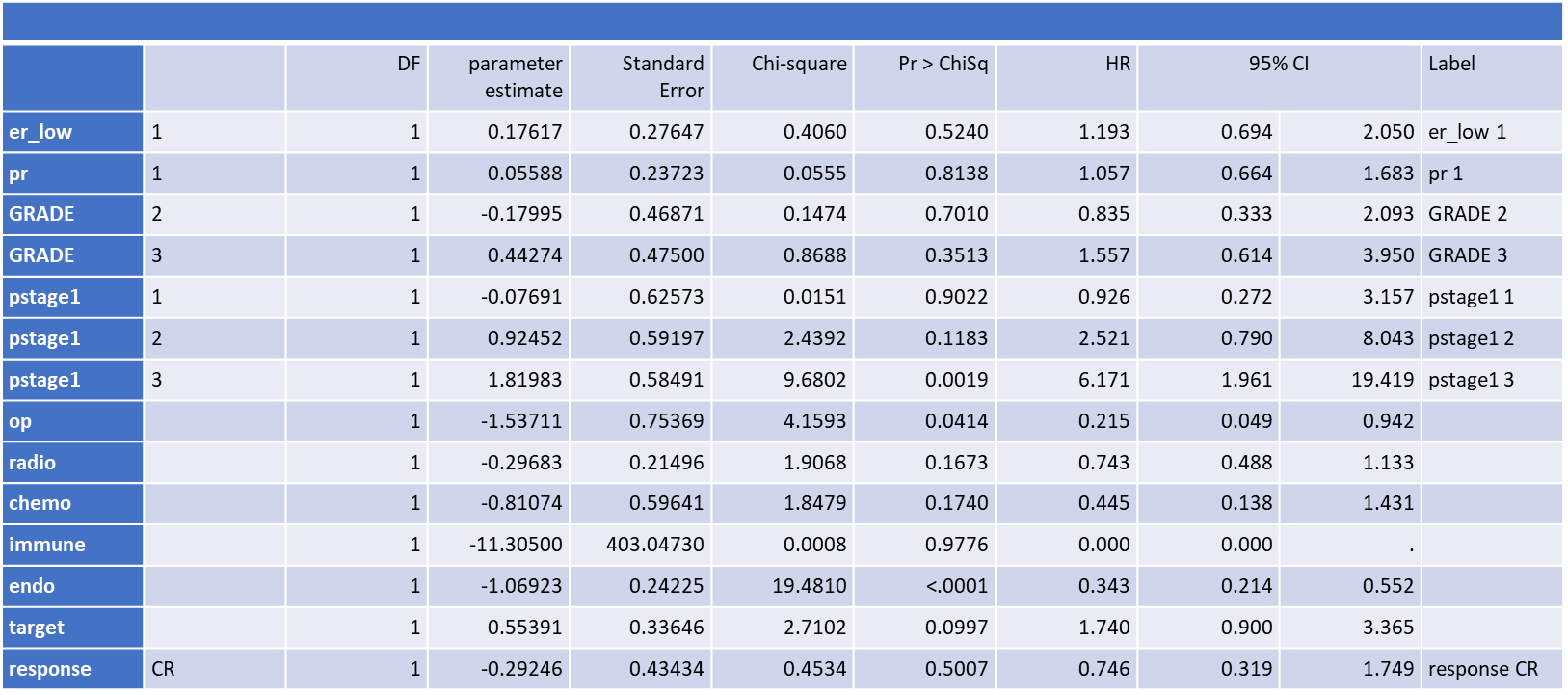


*DF= Degrees of Freedom, Pr>ChiSq=p-value, HR=Hazard ratio, CI=Confidence interval

## Table A3c. The recurrence free survival analysis by cox regression model with neoadjuvant treatment response


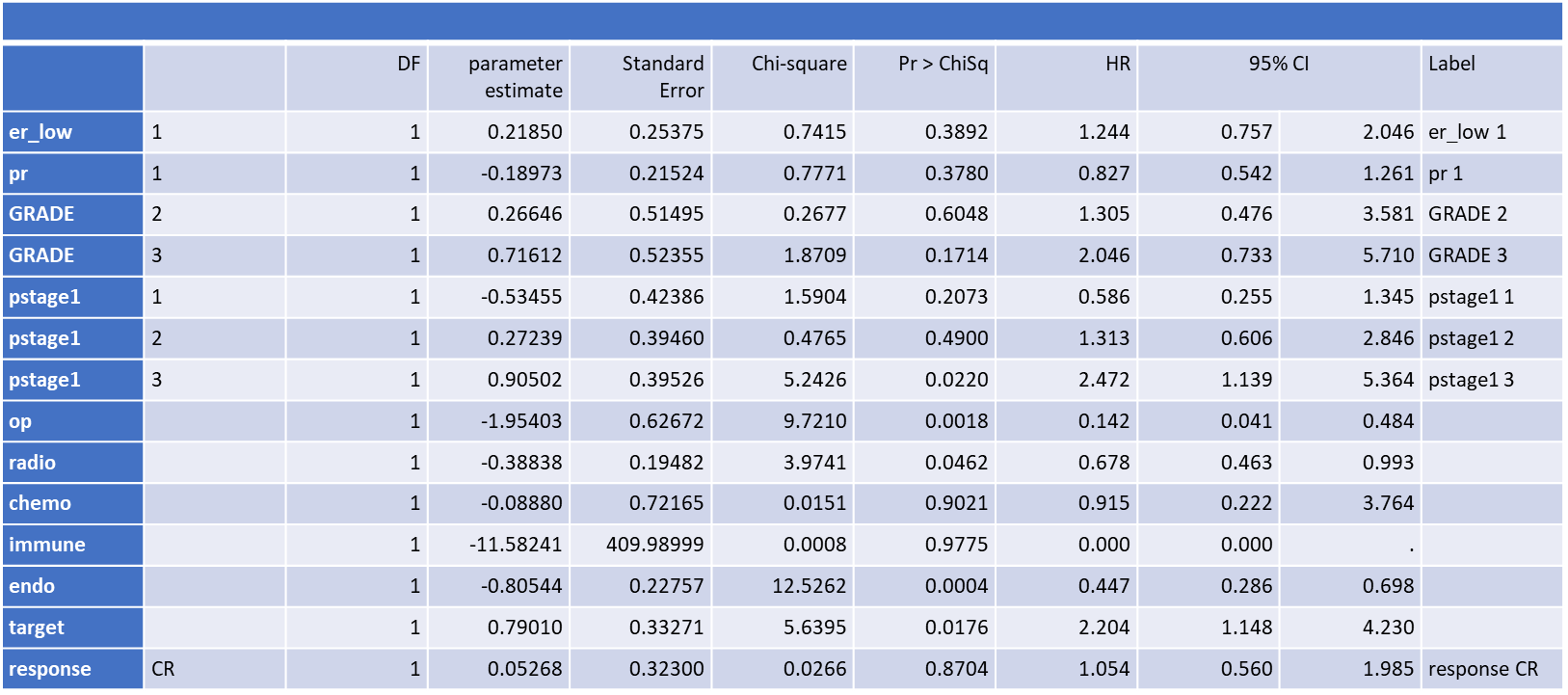


*DF= Degrees of Freedom, Pr>ChiSq=p-value, HR=Hazard ratio, CI=Confidence interval

## Table A3d. Chronological adoption of neoadjuvant or adjuvant therapy


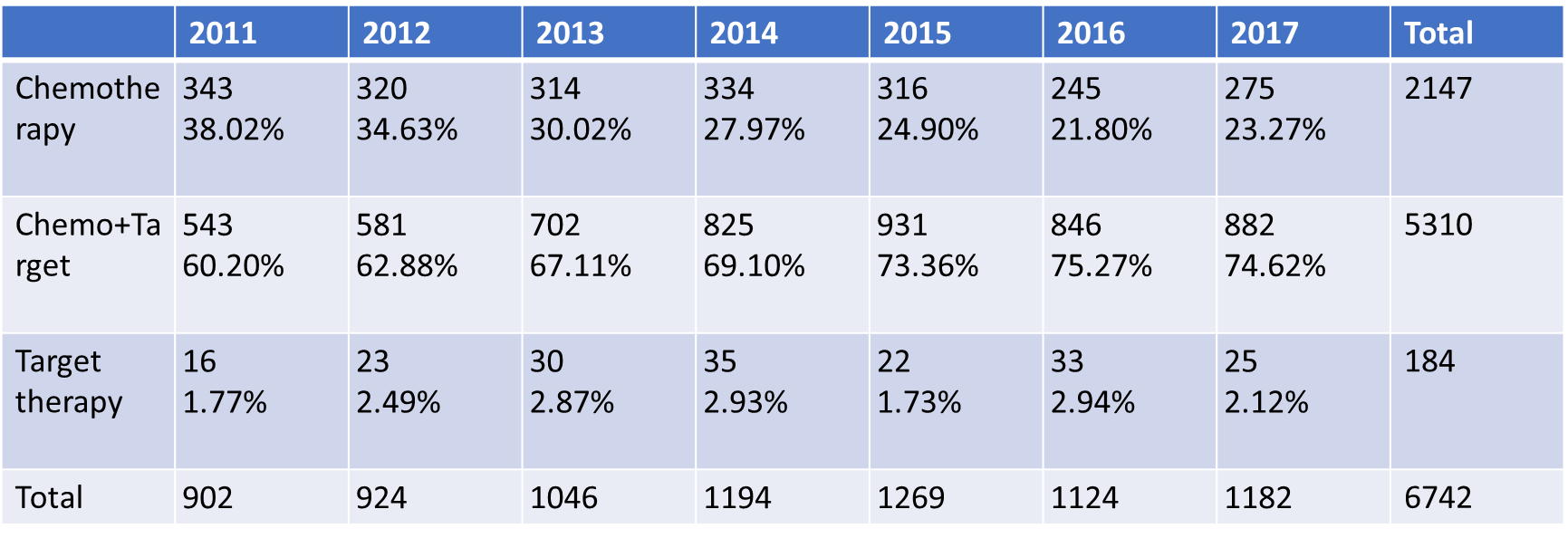


## Table A3e. Chronological adoption of adjuvant therapy


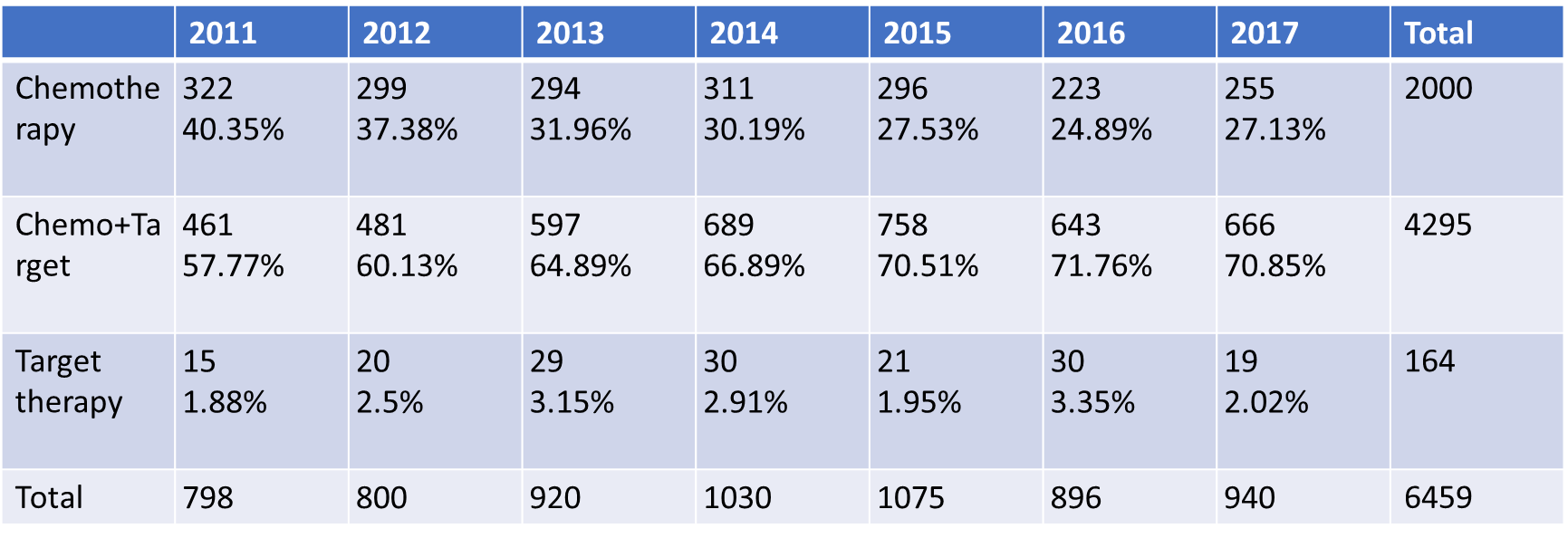


## Table A3e. Chronological adoption of neoadjuvant therapy


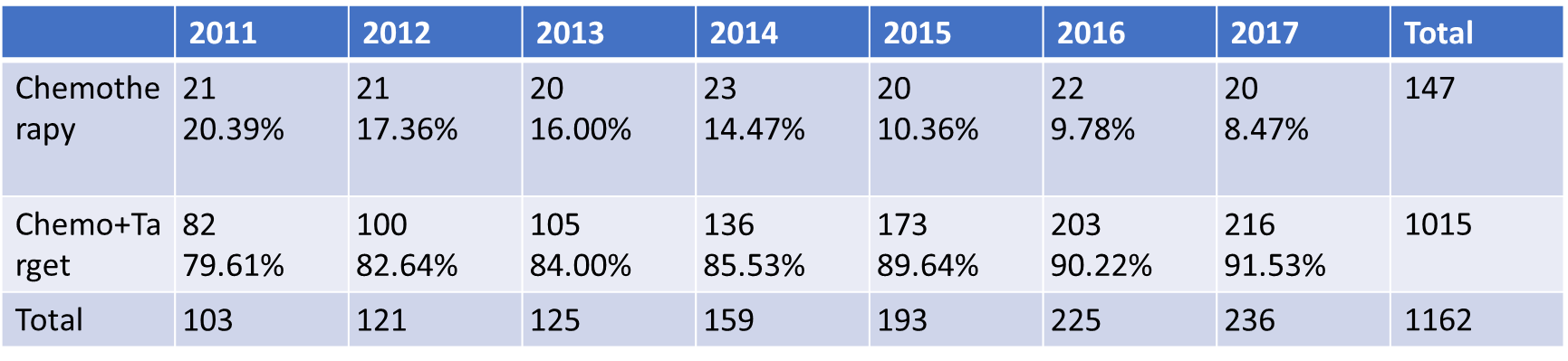


## Table A4. Endocrine therapy distribution

| Regimen | | | Case number | | |  |
| --- | --- | --- | --- | --- | --- | --- |
| Tamoxifen only | | | 3,483 (41.09%) | | |  |
| Aromatase inhibitors only | | | 2,453 (28.94%) | | |  |
| Sequential tamoxifen + AI | | | 2,541 (29.98%) | | |  |
| Total | | | 8,477 (100%) | | |  |
| **Sequence** | **Frequency** | **Percentage** | | **Cumulative frequency** | **Cumulative percentage** | |
| **A** | 70 | 0.83 | | 70 | 0.83 | |
| **A E** | 5 | 0.06 | | 75 | 0.88 | |
| **A E L** | 14 | 0.17 | | 89 | 1.05 | |
| **A E LT** | 48 | 0.57 | | 137 | 1.62 | |
| **A E T** | 14 | 0.17 | | 151 | 1.78 | |
| **A L** | 85 | 1.00 | | 236 | 2.78 | |
| **A LT** | 155 | 1.83 | | 391 | 4.61 | |
| **A T** | 181 | 2.14 | | 572 | 6.75 | |
| **E** | 18 | 0.21 | | 590 | 6.96 | |
| **E L** | 104 | 1.23 | | 694 | 8.19 | |
| **E LT** | 185 | 2.18 | | 879 | 10.37 | |
| **E T** | 82 | 0.97 | | 961 | 11.34 | |
| **L** | 2157 | 25.45 | | 3118 | 36.78 | |
| **LT** | 1876 | 22.13 | | 4994 | 58.91 | |
| **T** | 3483 | 41.09 | | 8477 | 100.00 | |

*A=Aromasin, E=Exemestane, L=Letrozole, T=Tamoxifen

## Table A4a. Endocrine therapy duration


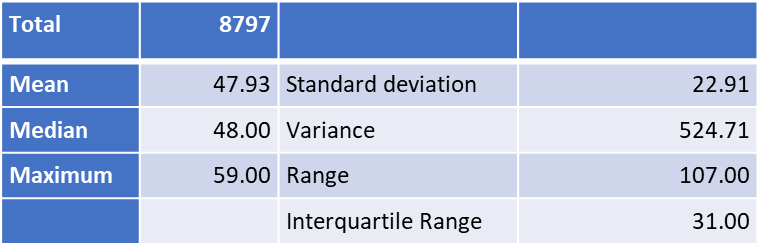


## Table A4b. The overall survival outcomes and endocrine therapy duration in subgroup of ER 1-9% patients


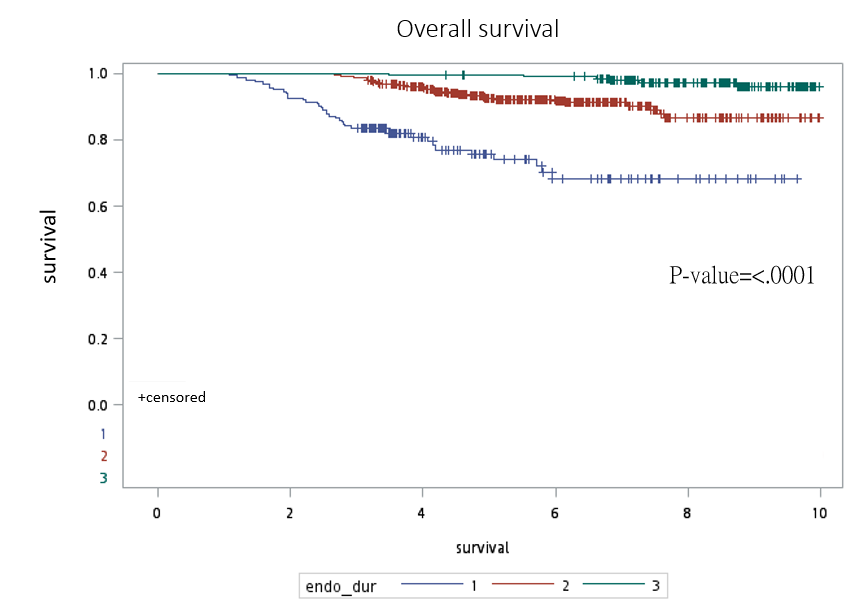


*endo_dur =1 means the endocrine therapy duration <=24 months

*endo_dur =2 means the endocrine therapy duration 25-60 months

*endo_dur =3 means the endocrine therapy duration >60 months

## Table A4c. The breast cancer specific survival outcomes and endocrine therapy duration in subgroup of ER 1-9% patients


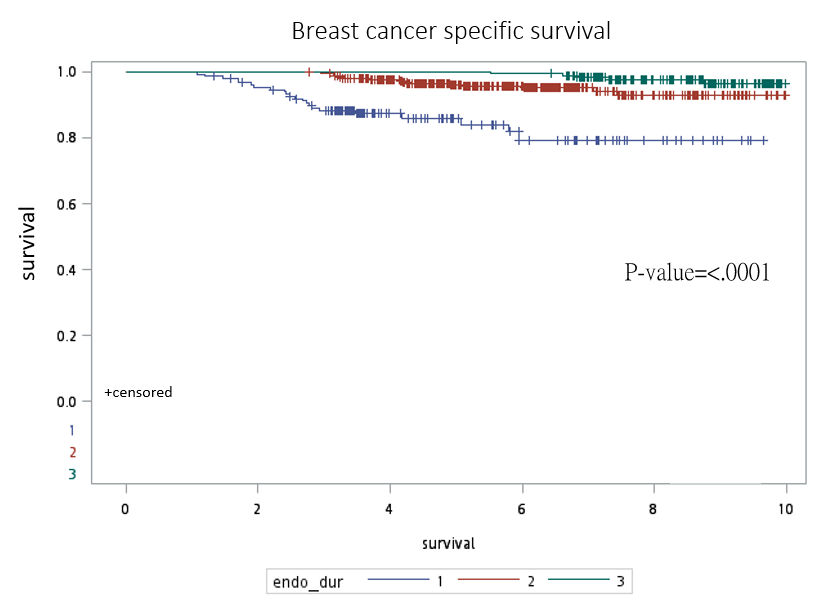


*endo_dur =1 means the endocrine therapy duration <=24 months

*endo_dur =2 means the endocrine therapy duration 25-60 months

*endo_dur =3 means the endocrine therapy duration >60 months

## Table A4d. The recurrence free survival outcomes and endocrine therapy duration in subgroup of ER 1-9% patients


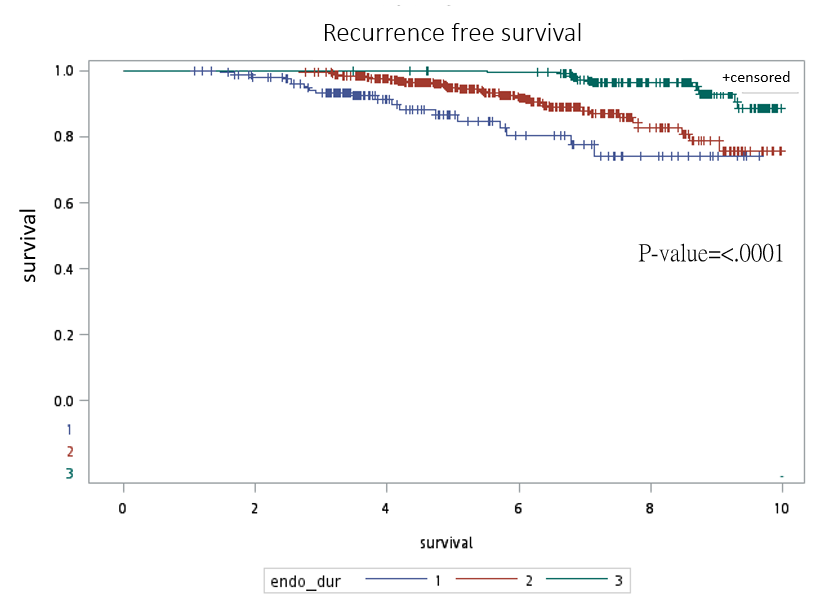


*endo_dur =1 means the endocrine therapy duration <=24 months

*endo_dur =2 means the endocrine therapy duration 25-60 months

*endo_dur =3 means the endocrine therapy duration >60 months

## Table A4e. The overall survival outcomes and endocrine therapy duration in subgroup of ER >=10% patients


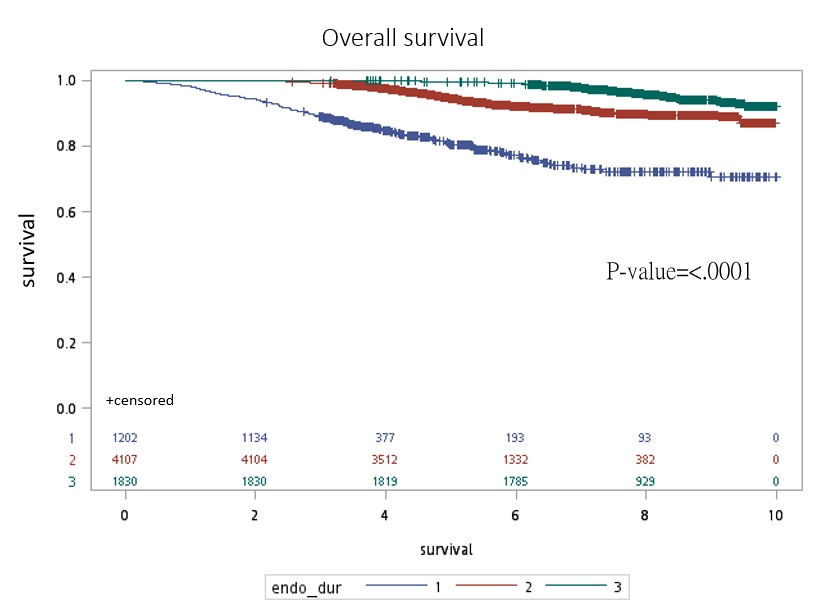


*endo_dur =1 means the endocrine therapy duration <=24 months

*endo_dur =2 means the endocrine therapy duration 25-60 months

*endo_dur =3 means the endocrine therapy duration >60 months

## Table A4f. The breast cancer specific survival outcomes and endocrine therapy duration in subgroup of ER >=10% patients


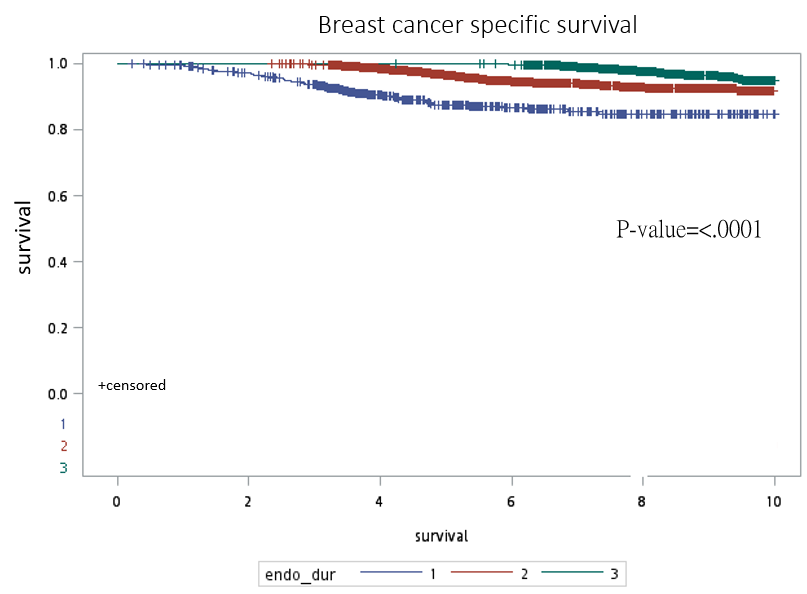


*endo_dur =1 means the endocrine therapy duration <=24 months

*endo_dur =2 means the endocrine therapy duration 25-60 months

*endo_dur =3 means the endocrine therapy duration >60 months

## Table A4g. The recurrence free survival outcomes and endocrine therapy duration in subgroup of ER >=10% patients


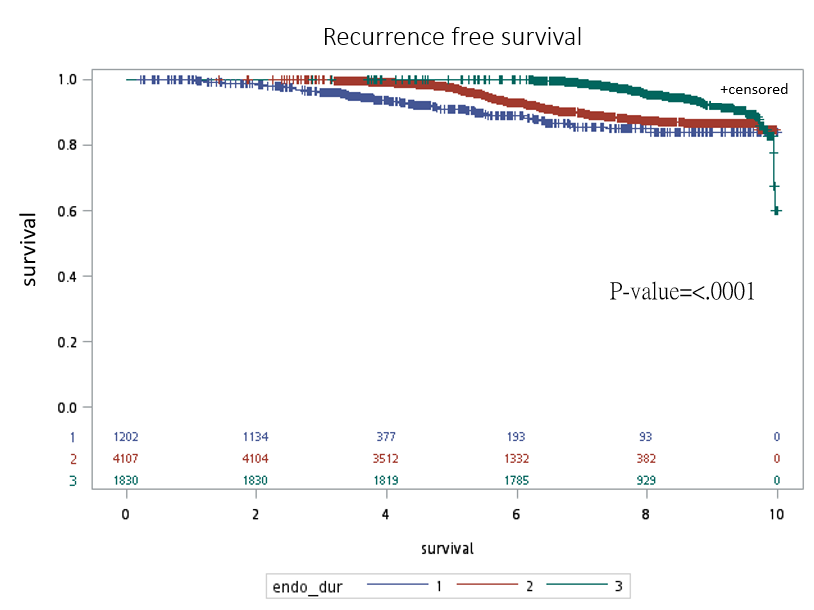


*endo_dur =1 means the endocrine therapy duration <=24 months

*endo_dur =2 means the endocrine therapy duration 25-60 months

*endo_dur =3 means the endocrine therapy duration >60 months

## Table A4h. The overall survival analysis by cox regression model with endocrine therapy duration


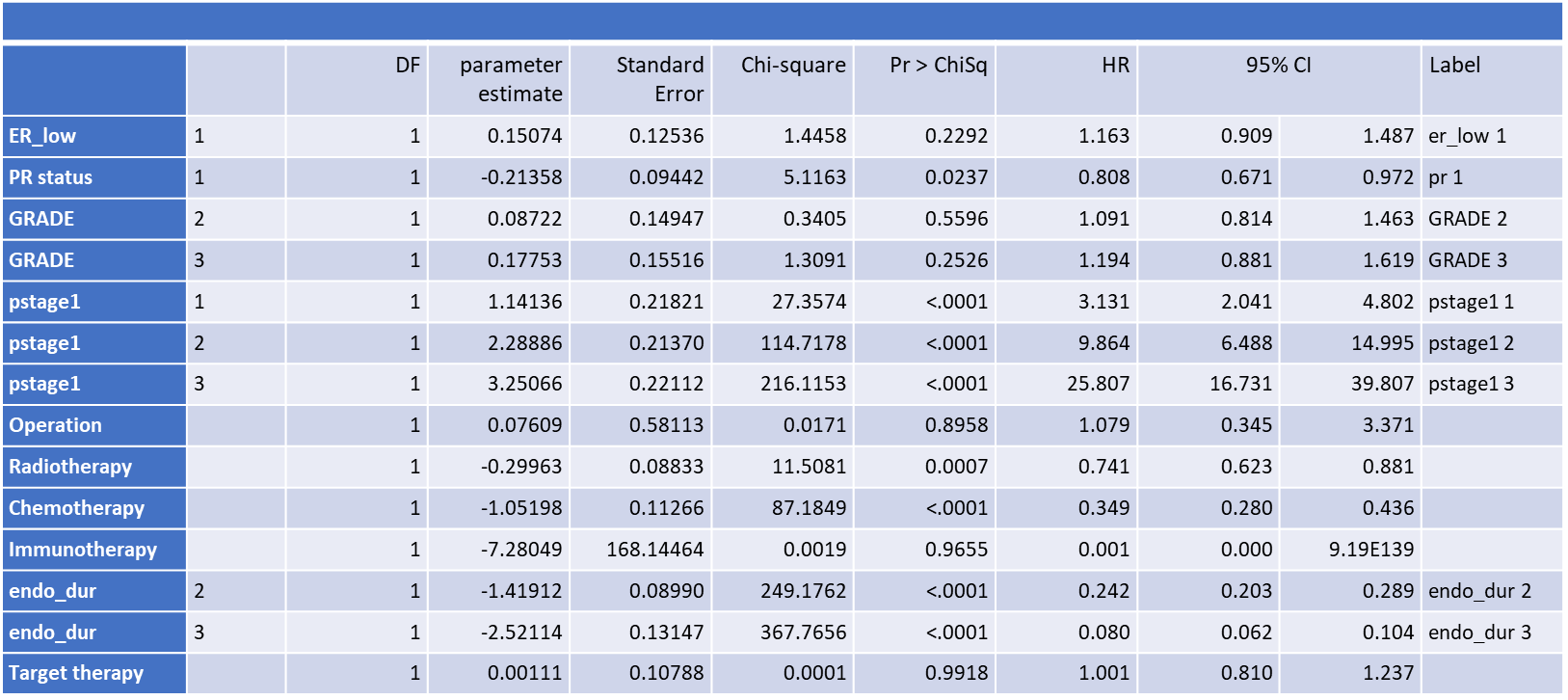


## Table A4i. The breast cancer specific survival analysis by cox regression model with endocrine therapy duration


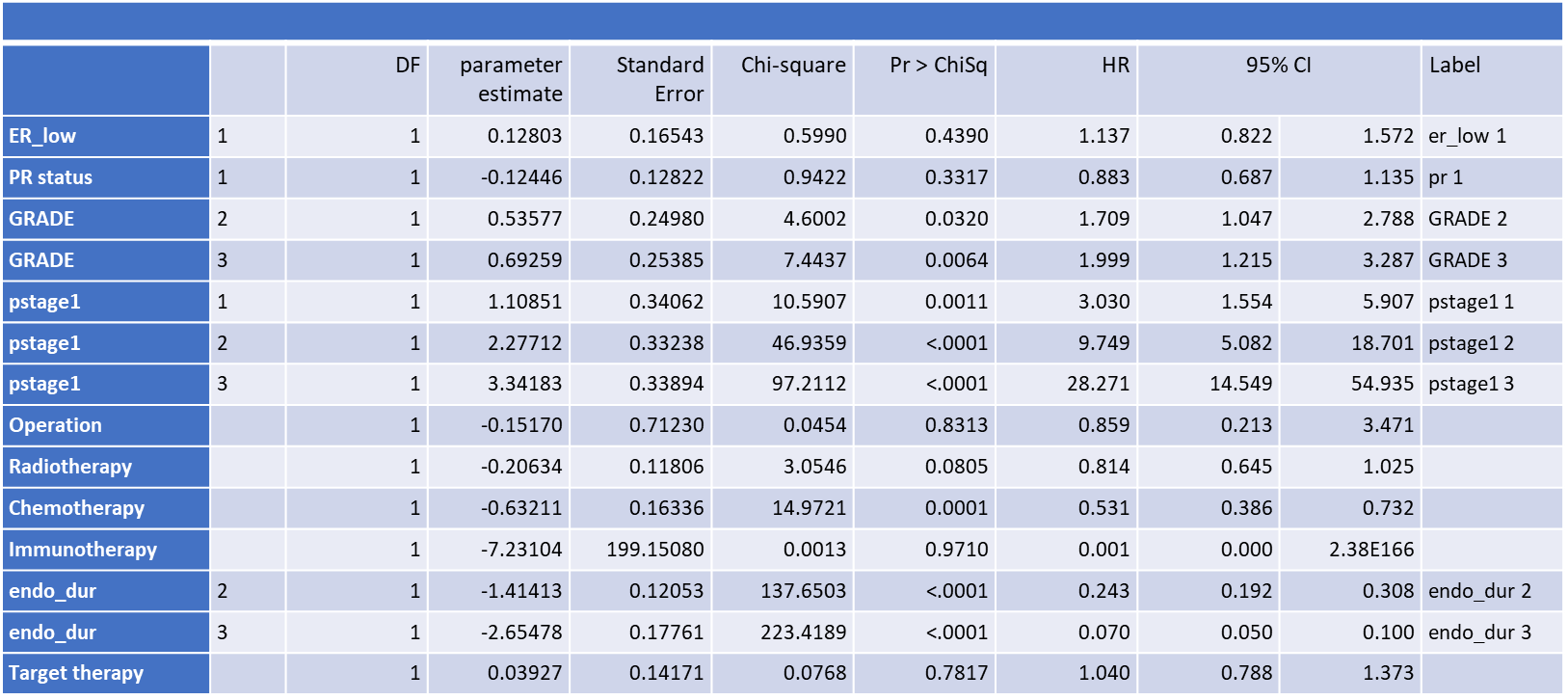


## Table A4j. The recurrence free survival analysis by cox regression model with endocrine therapy duration


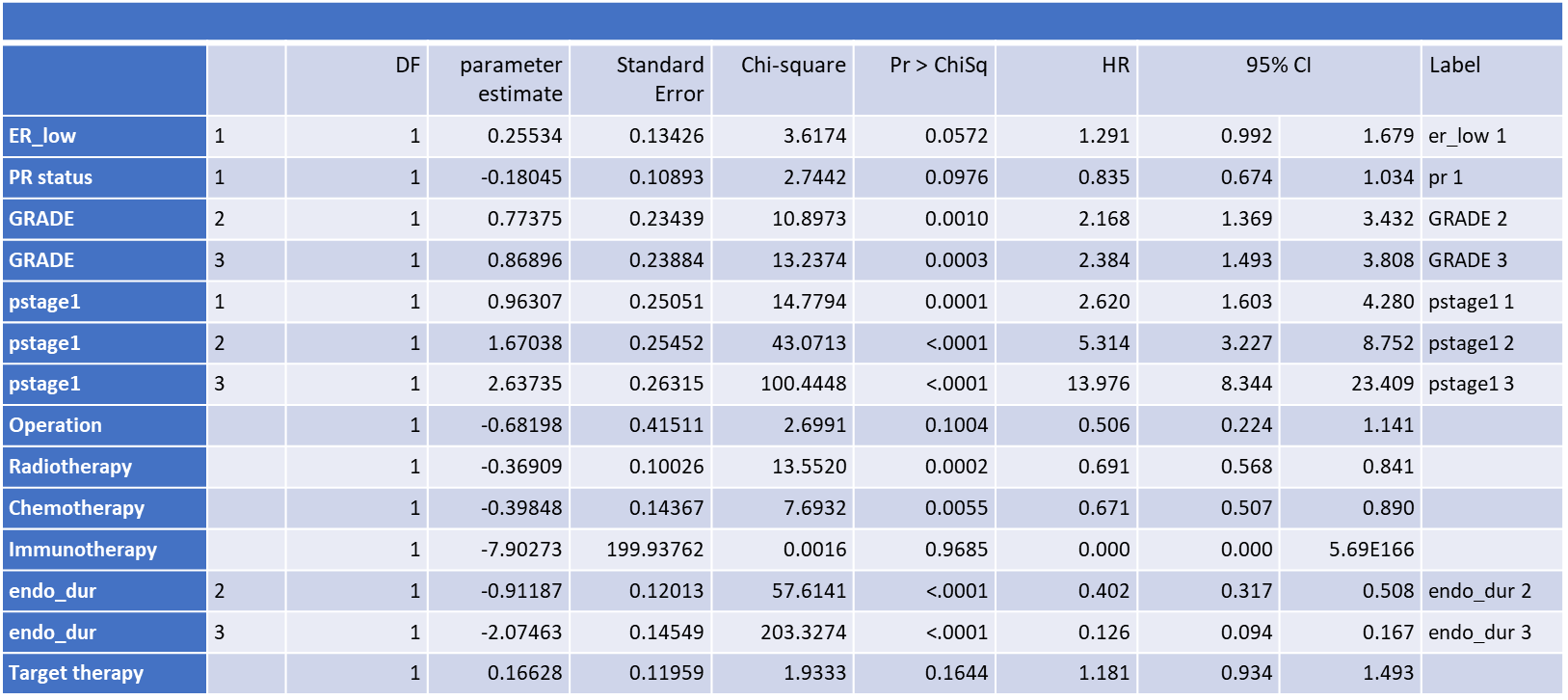


## Table A5. Endocrine therapy duration and survival outcomes

| Subgroup | OS (P-value) | BCSS (P-value) | RFS (P-value) |
| --- | --- | --- | --- |
| ER 1-9% | <0.0001 | <0.0001 | <0.0001 |
| ER >=10% | <0.0001 | <0.0001 | <0.0001 |

## Table A5a. The overall survival outcomes analysis by cox regression model in subgroup of low ER presentation

##
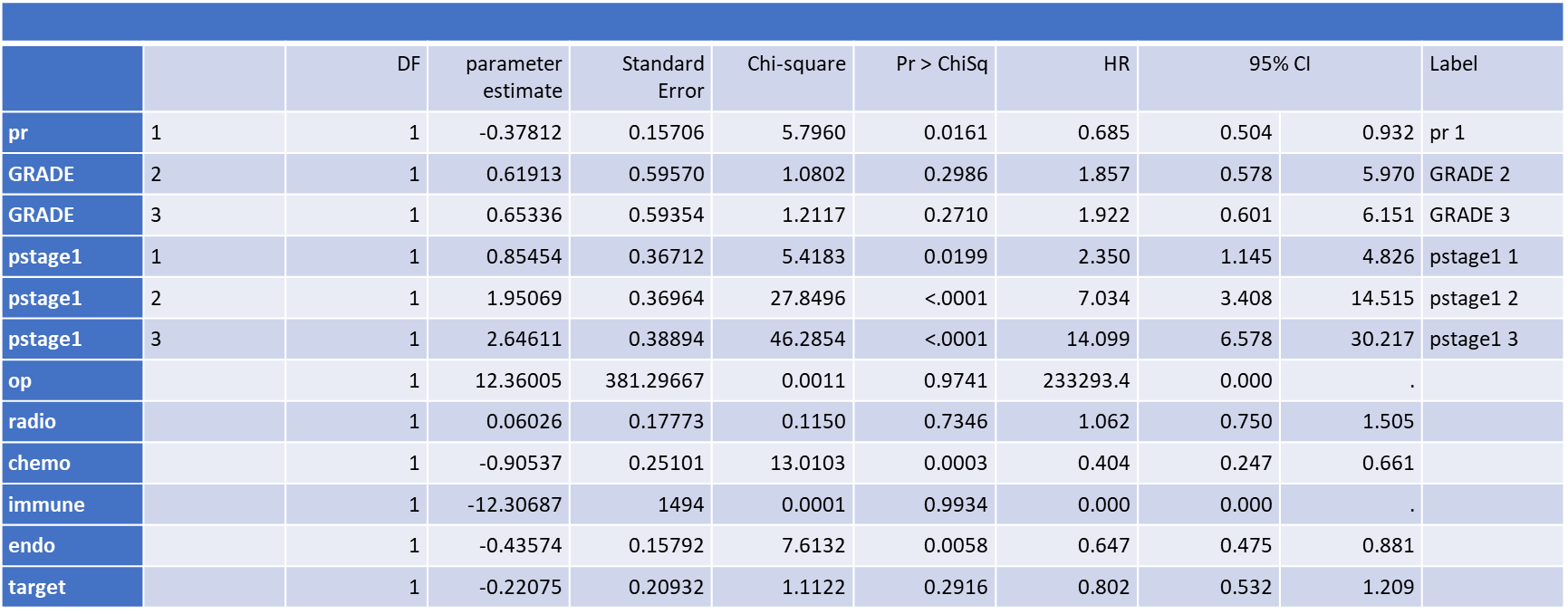


## Table A5b. The breast cancer survival outcomes analysis by cox regression model in subgroup of low ER presentation


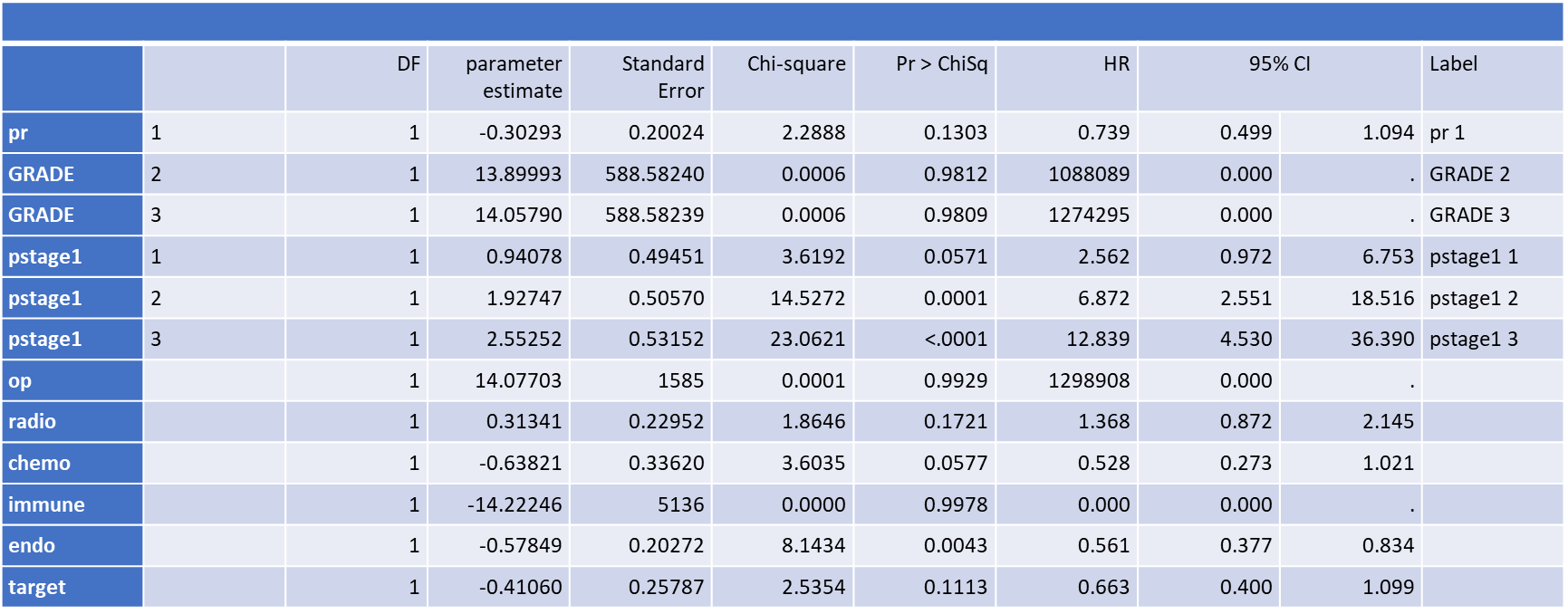


## Table A5c. The recurrence free survival outcomes analysis by cox regression model in subgroup of low ER presentation


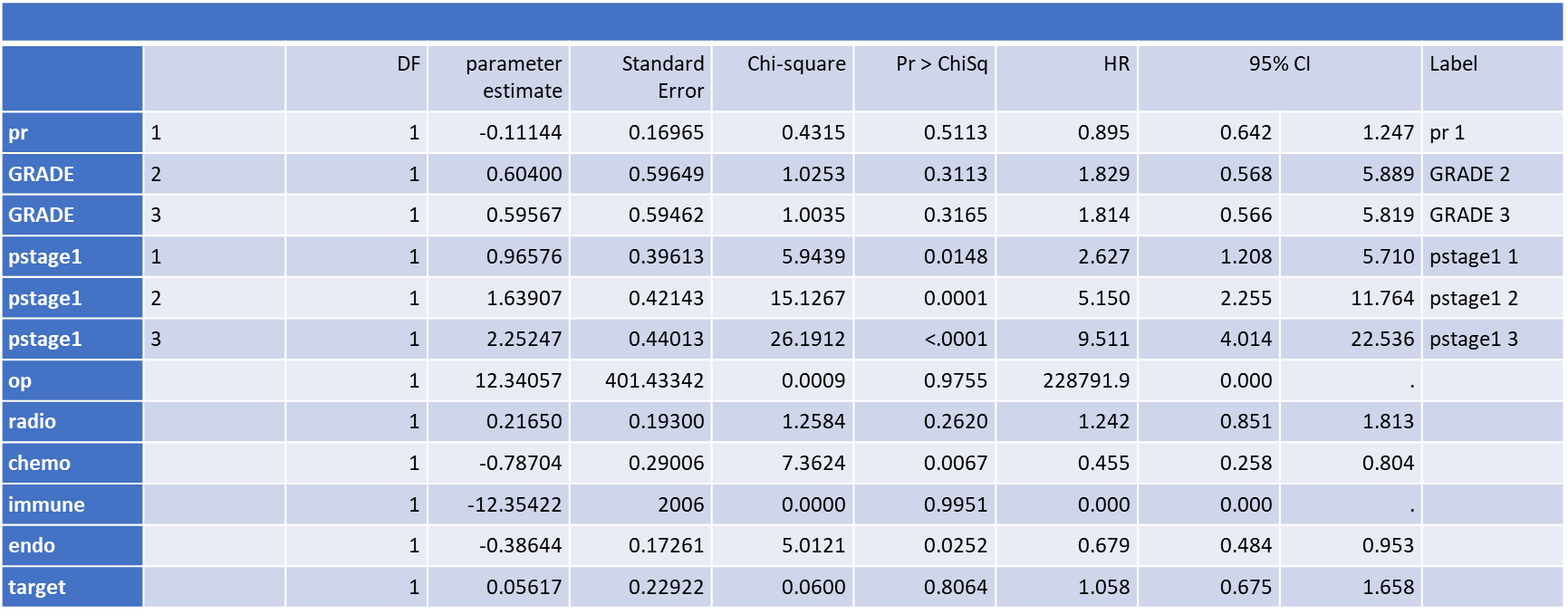


## Table A5d. The overall survival outcomes analysis by cox regression model in subgroup of ER>=10% presentation


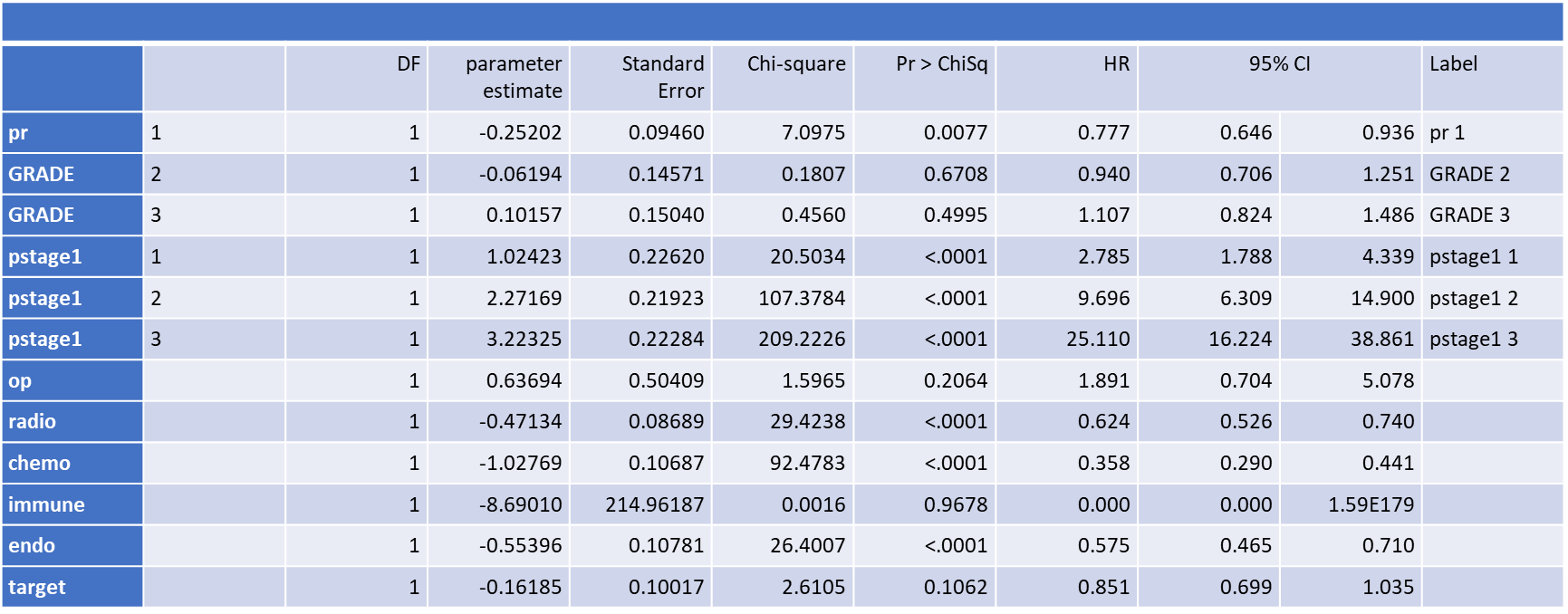


## Table A5e. The breast cancer specific survival outcomes analysis by cox regression model in subgroup of ER>=10% presentation


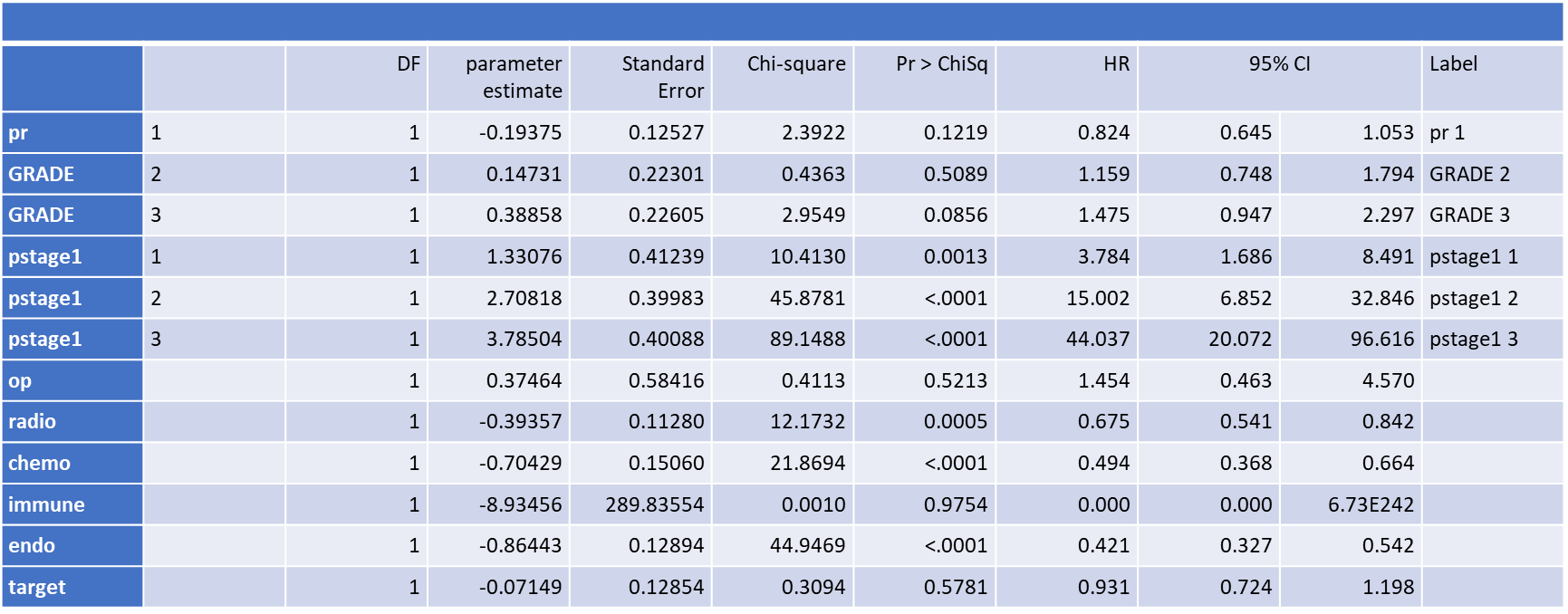


## Table A5f. The recurrence free survival outcomes analysis by cox regression model in subgroup of ER>=10% presentation


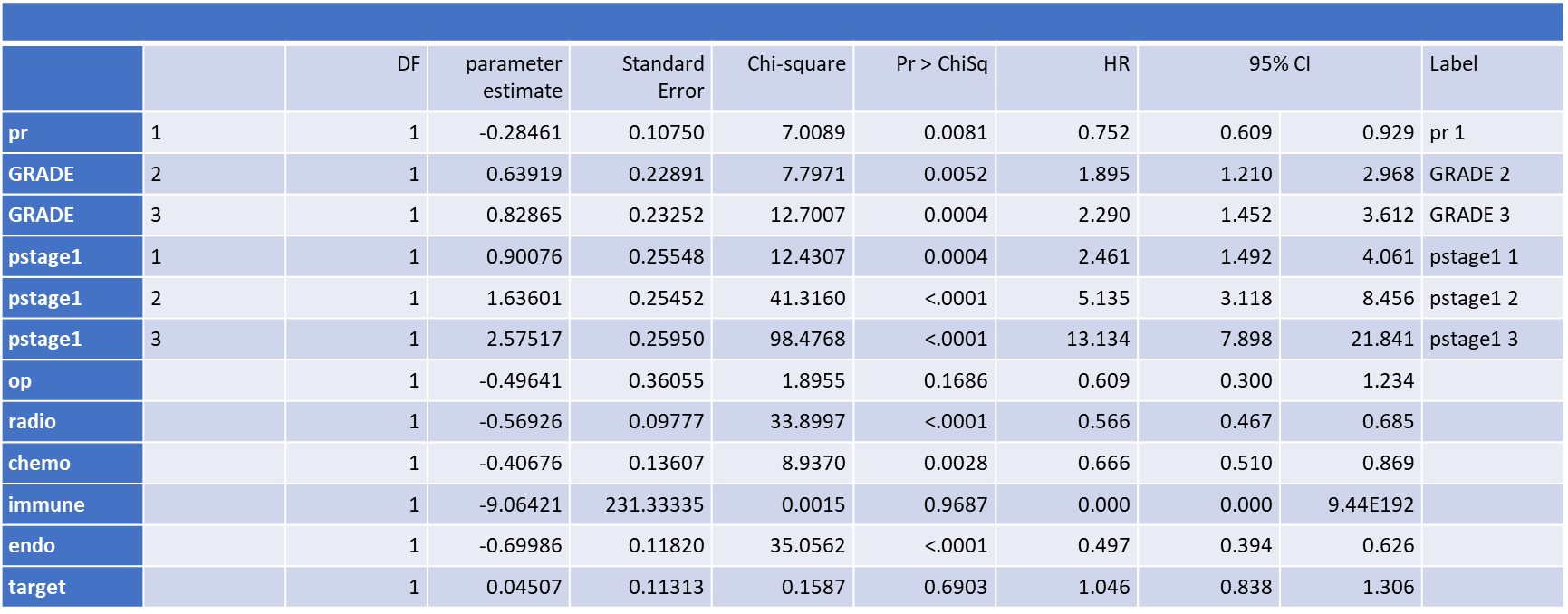


## Table A6. Chemotherapy regimens

| Regimen type | Number of patients |
| --- | --- |
| Taxane only | 523 (8.27%) |
| Anthracycline only | 2,305 (36.46%) |
| Alkylating agents only | 17 (0.27%) |
| Taxane + Anthracycline | 3,166 (50.08%) |
| Taxane + Alkylating agents | 70 (1.11%) |
| Anthracycline + Alkylating agents | 15 (0.24%) |
| Triple combination | 226 (3.57%) |
| Total | 6,322 (100%) |
| Missing data | 385 |

|  | **sequence** | Number of patients |
| --- | --- | --- |
| **1** | eip_doc_ | 1952 |
| **2** | eip_ | 1776 |
| **3** | dox_doc_ | 684 |
| **4** | dox_ | 510 |
| **5** | doc_ | 407 |
| **6** |  | 385 |
| **7** | eip_pac_ | 179 |
| **8** | dox_pac_ | 119 |
| **9** | eip_doc_cis | 104 |
| **10** | eip_doc_pac_ | 103 |
| **11** | pac_ | 91 |
| **12** | dox_doc_cis | 40 |
| **13** | doc_cis | 39 |
| **14** | dox_doc_pac_ | 30 |
| **15** | eip_doc_pac_cis | 29 |
| **16** | doc_pac_ | 25 |
| **17** | eip_lip_doc_ | 23 |
| **18** | dox_eip_doc_ | 19 |
| **19** | doc_car_ | 18 |
| **20** | dox_doc_pac_cis | 18 |
| **21** | eip_lip_doc_pac_ | 17 |
| **22** | cis | 13 |
| **23** | dox_eip_ | 12 |
| **24** | lip_doc_ | 11 |
| **25** | lip_pac_ | 11 |
| **26** | dox_lip_doc_pac_ | 10 |
| **27** | eip_cis | 10 |
| **28** | eip_lip_doc_pac_cis | 9 |
| **29** | eip_doc_car_ | 7 |
| **30** | eip_doc_pac_car_ | 7 |
| **31** | lip_ | 7 |
| **32** | eip_lip_doc_cis | 6 |
| **33** | eip_pac_cis | 6 |
| **34** | doc_pac_cis | 5 |
| **35** | dox_cis | 5 |
| **36** | car_ | 4 |
| **37** | doc_pac_car_cis | 4 |
| **38** | dox_eip_doc_pac_ | 4 |
| **39** | lip_doc_pac_ | 4 |
| **40** | pac_cis | 4 |

*PAC = Paclitaxel; DOC= Docetaxel; DOX= Doxorubicin; EPI = Epirubicin; LIP = Lipo-dox (liposomal doxorubicin); CAR= Carboplatin; CIS= Cisplatin; CYC= Cyclophosphamide

(The medications mentioned above indicate the types of drugs previously administered to the patients, rather than the specific treatment regimens. In our database, the majority of patients received a Taxane plus Anthracycline regimen.)

## Table A6a. Duration of chemotherapy

| Total | 7037 |  |  |
| --- | --- | --- | --- |
| Mean | 7.84 | Standard deviation | 13.27 |
| Median | 5.00 | Variance | 176.03 |
| Maximum | 5.00 | Range | 107.00 |
|  |  | Interquartile Range | 2.00 |

## Table A7. Systemic therapy and survival outcomes

| Subgroup | OS (*P*-value) | BCSS (*P*-value) | RFS (*P*-value) |
| --- | --- | --- | --- |
| ER 1-9% | 0.3279 | 0.1447 | 0.0530 |
| ER >=10% | 0.0360 | 0.0121 | 0.0003 |

* ER_low=1 overall survival

| **Stratified** | **Systemic treatment** | **total** | **failed** | **censored** | **censored percentage** |
| --- | --- | --- | --- | --- | --- |
| 1 | 0 | 376 | 39 | 337 | 89.63 |
| 2 | 1 | 1059 | 130 | 929 | 87.72 |
| total |  | 1435 | 169 | 1266 | 88.22 |

* ER_low=1 breast cancer specific survival

| **Stratified** | **Systemic treatment** | **total** | **failed** | **censored** | **censored percentage** |
| --- | --- | --- | --- | --- | --- |
| 1 | 0 | 336 | 20 | 316 | 94.05 |
| 2 | 1 | 980 | 83 | 897 | 91.53 |
| total |  | 1316 | 103 | 1213 | 92.17 |

* ER_low=1 recurrence free survival

| **Stratified** | **Systemic treatment** | **total** | **failed** | **censored** | **censored percentage** |
| --- | --- | --- | --- | --- | --- |
| 1 | 0 | 376 | 28 | 348 | 92.55 |
| 2 | 1 | 1059 | 116 | 943 | 89.05 |
| total |  | 1435 | 144 | 1291 | 89.97 |

* ER_low=0 overall survival

| **Stratified** | **Systemic treatment** | **total** | **failed** | **censored** | **censored percentage** |
| --- | --- | --- | --- | --- | --- |
| 1 | 0 | 2090 | 200 | 1890 | 90.43 |
| 2 | 1 | 5742 | 459 | 5283 | 92.01 |
| total |  | 7832 | 659 | 7173 | 91.59 |

* ER_low=0 breast cancer specific survival

| **Stratified** | **Systemic treatment** | **total** | **failed** | **censored** | **censored percentage** |
| --- | --- | --- | --- | --- | --- |
| 1 | 0 | 1836 | 79 | 1757 | 95.70 |
| 2 | 1 | 5375 | 314 | 5061 | 94.16 |
| total |  | 7211 | 393 | 6818 | 94.55 |

* ER_low=0 recurrence free survival

| **Stratified** | **Systemic treatment** | **total** | **failed** | **censored** | **censored percentage** |
| --- | --- | --- | --- | --- | --- |
| 1 | 0 | 2090 | 101 | 1989 | 95.17 |
| 2 | 1 | 5742 | 404 | 5338 | 92.96 |
| total |  | 7832 | 505 | 7327 | 93.55 |

## Table A7a. Chronological adoption of systemic treatment


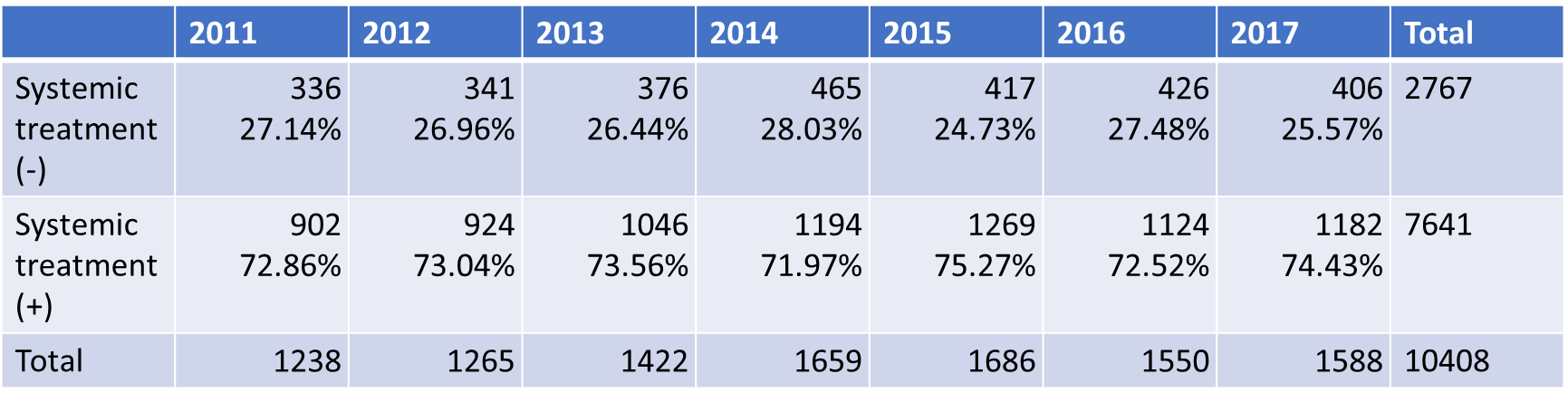


## Table A7b. The overall survival of subgroup with ER-low status (1-9%)


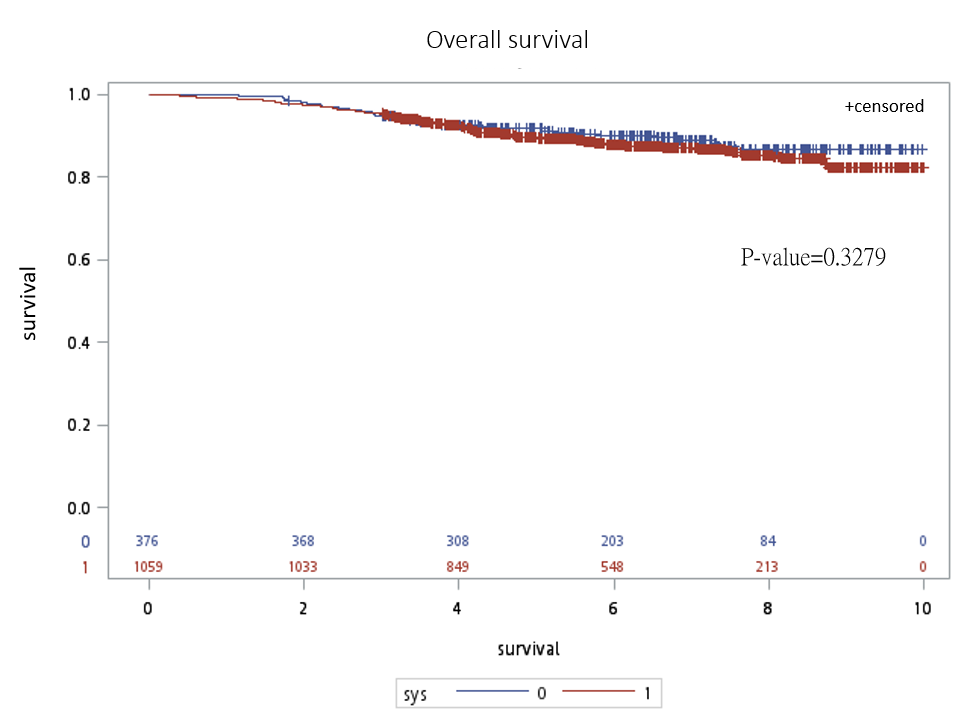


*sys=0 means without systemic treatment

*sys=1 means with systemic treatment

## Table A7c. The breast cancer specific survival of subgroup of with ER-low status (1-9%)


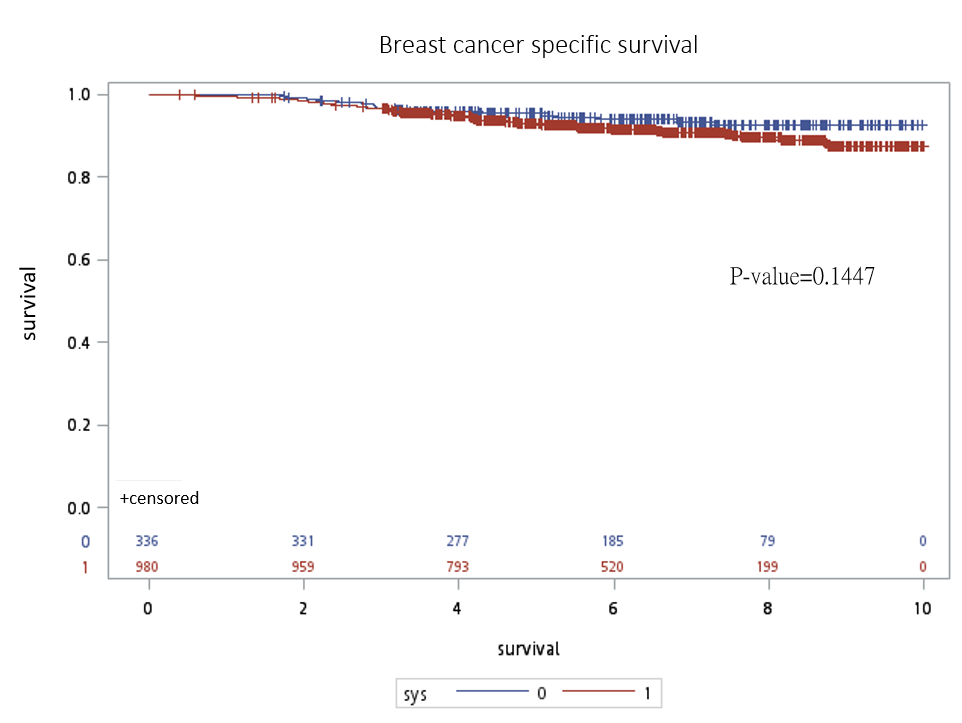


*sys=0 means without systemic treatment

*sys=1 means with systemic treatment

## Table A7d. The recurrence free survival of subgroup with ER-low status (1-9%)


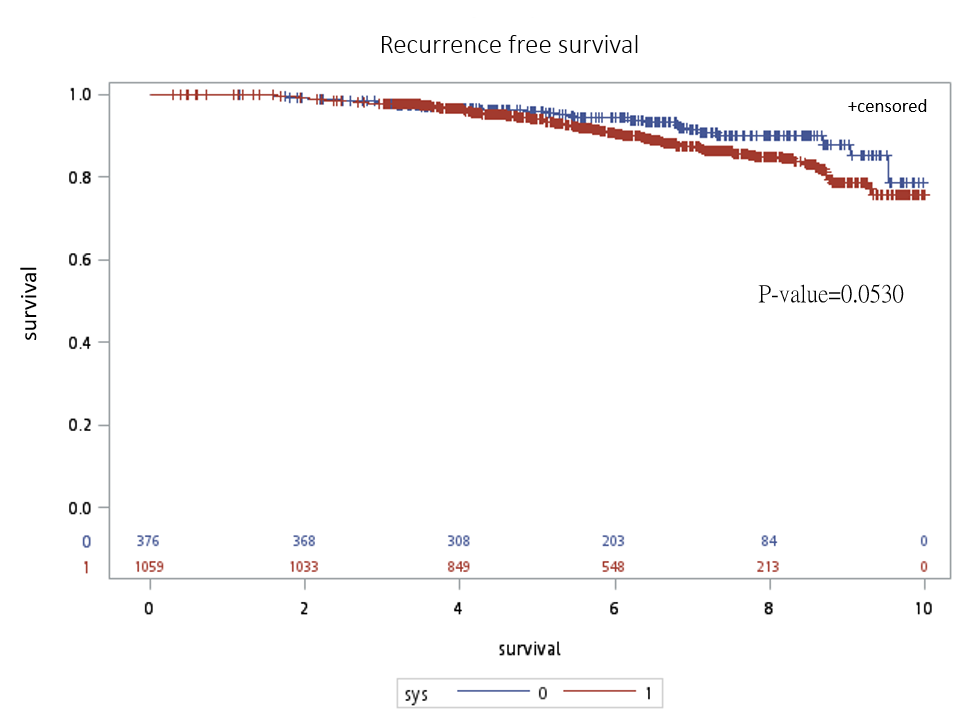


*sys=0 means without systemic treatment

*sys=1 means with systemic treatment

## Table A7e. The overall survival of subgroup with positive ER status (>=10%)


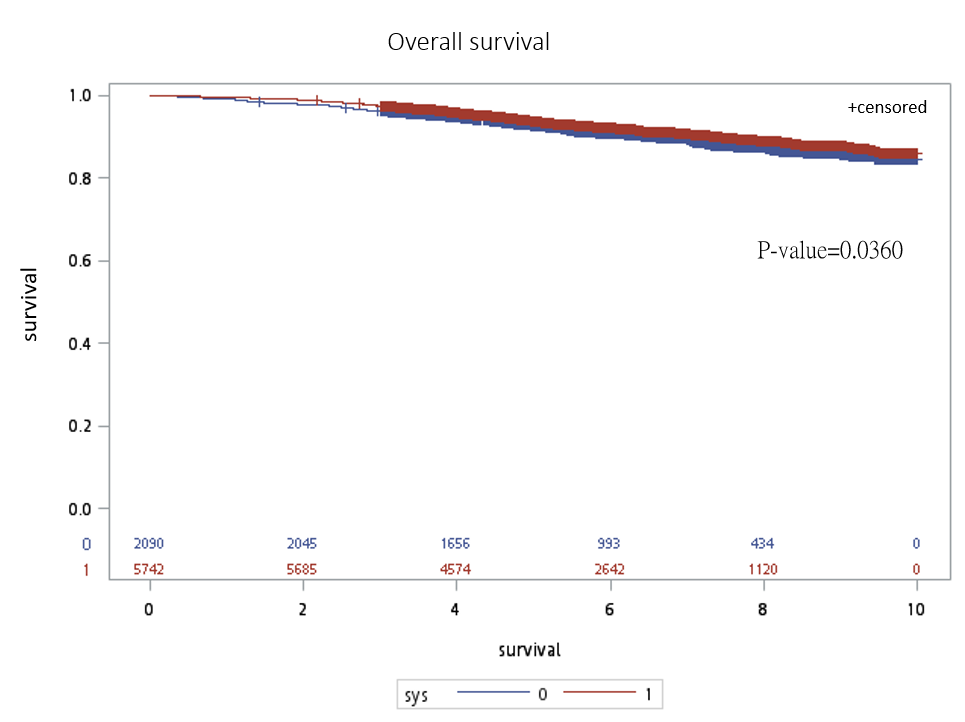


*sys=0 means without systemic treatment

*sys=1 means with systemic treatment

## Table A7f. The breast cancer specific survival of subgroup with positive ER status (>=10%)


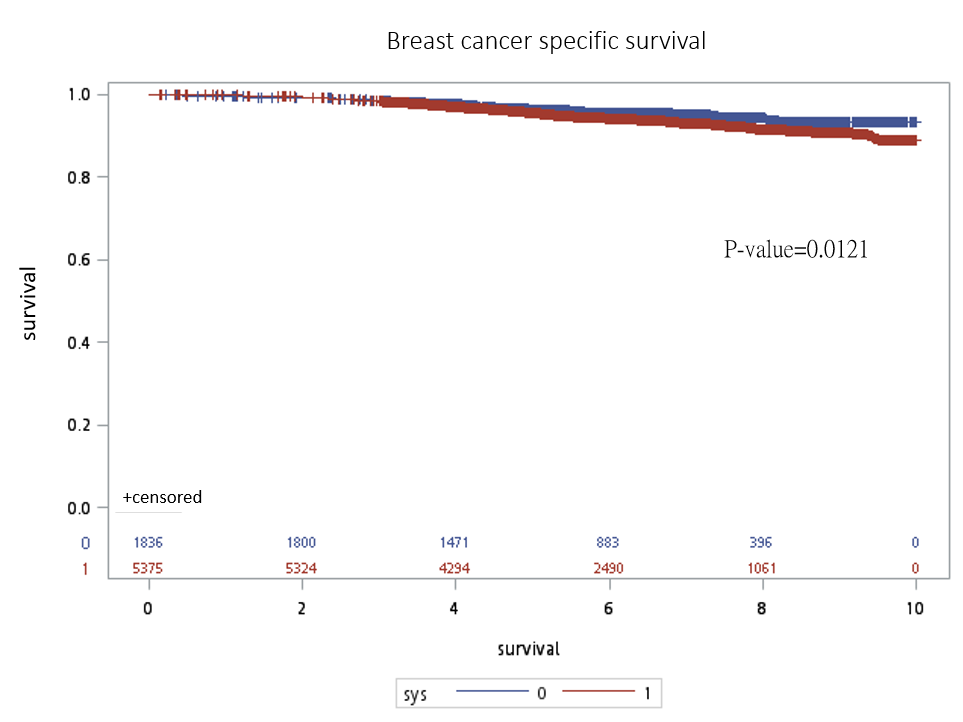


*sys=0 means without systemic treatment

*sys=1 means with systemic treatment

## Table A7g. The recurrence free survival of subgroup with positive ER status (>=10%)


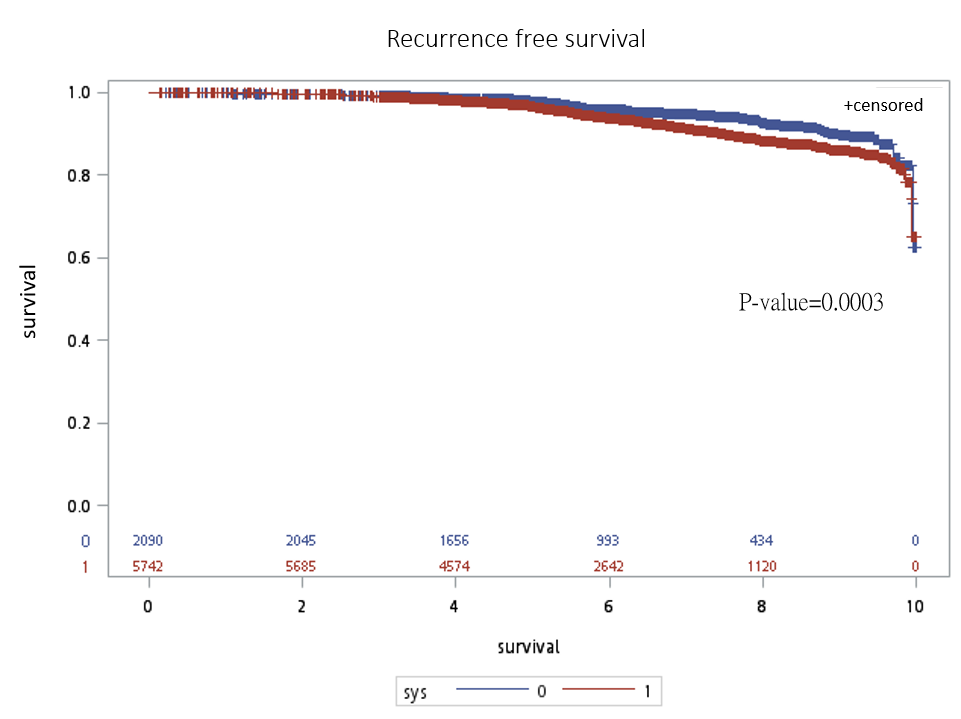


*sys=0 means without systemic treatment

*sys=1 means with systemic treatment

## Table A8. The PR status and endocrine therapy


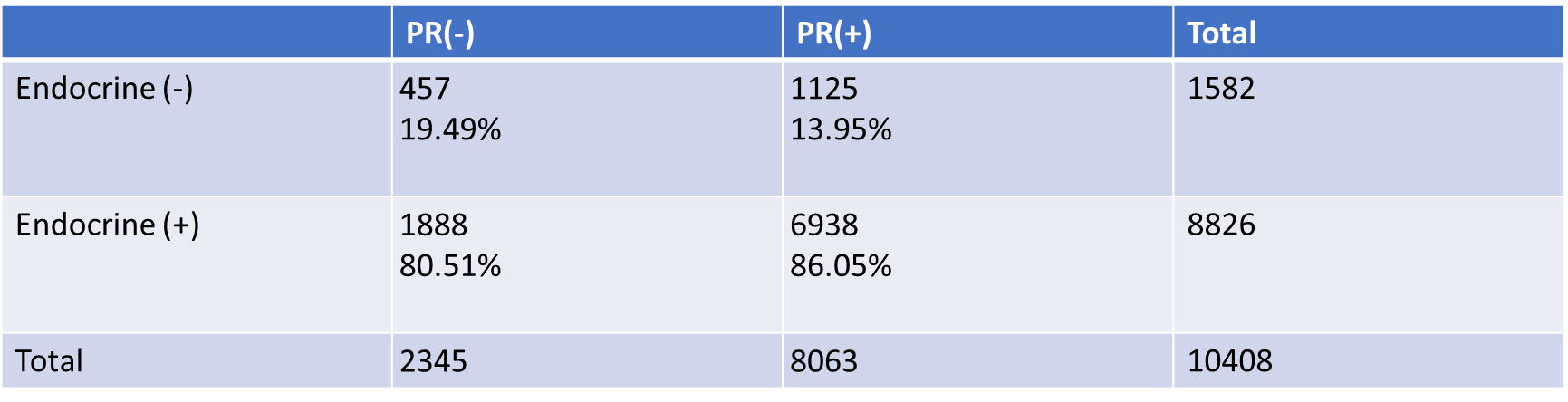


* *P*-value=<.0001

## Table A8a. The PR status and endocrine therapy in the subgroup of ER >=10%


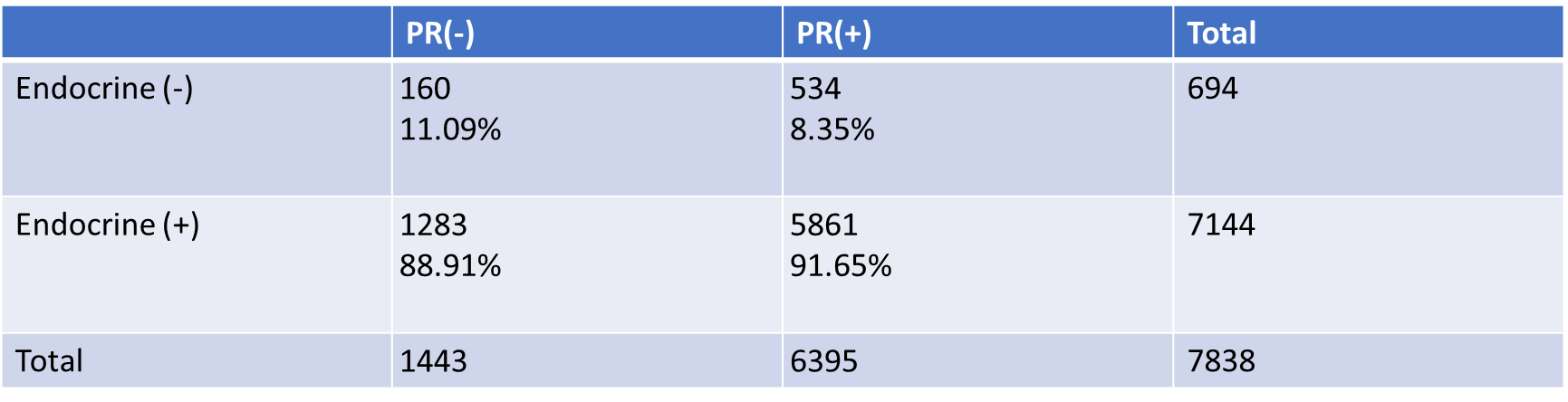


**P*-value=0.0009

## Table A8b. The PR status and endocrine therapy in the subgroup of ER 1-9%


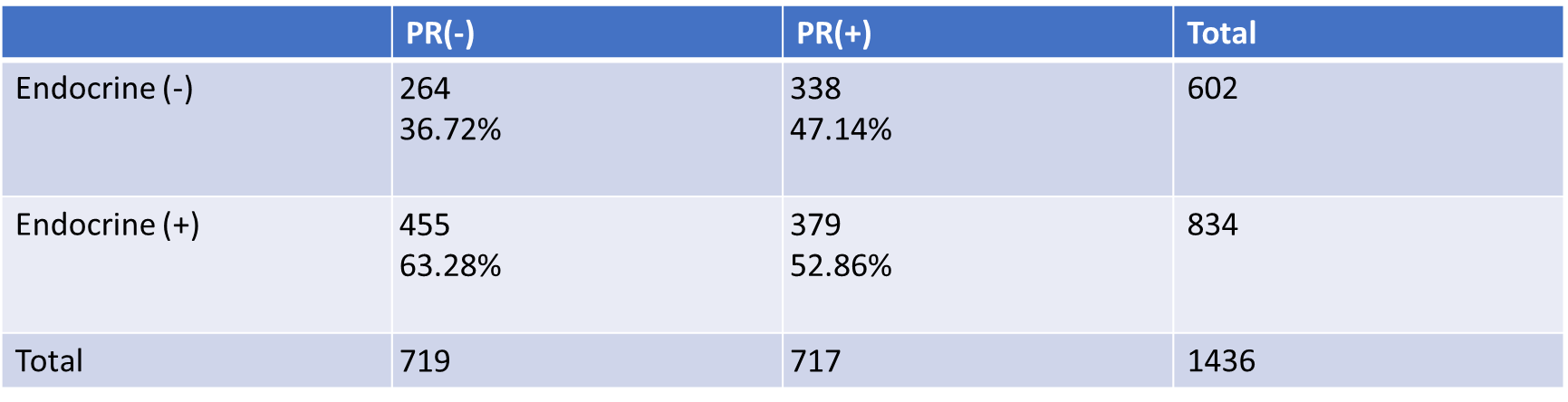


* *P*-value=<.0001

## Table A8c. The survival time (years) of follow-up to 10 years with PR status


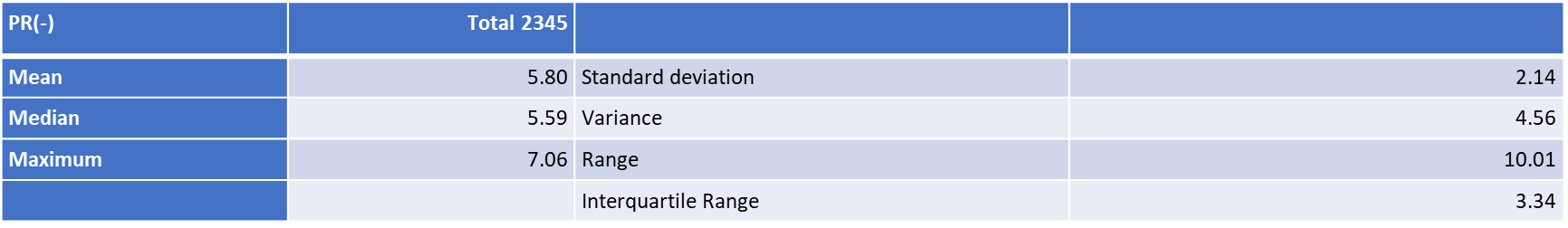


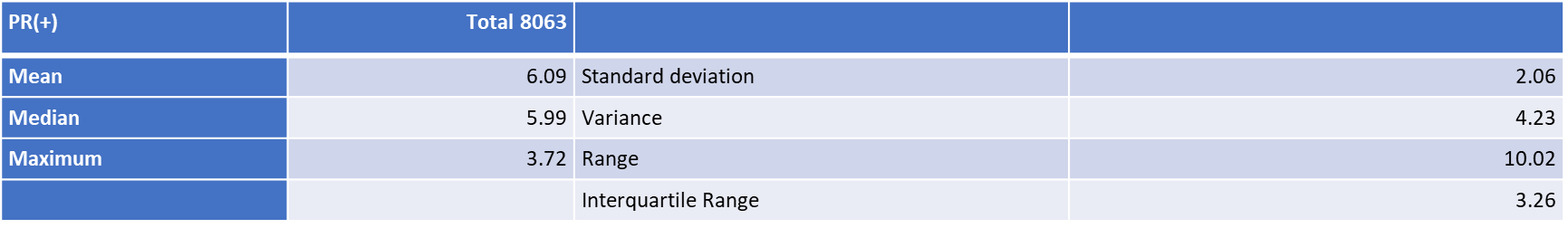


## Table A8d. The overall survival outcomes analysis by cox regression model in subgroup of ER 1-9% and PR (+)


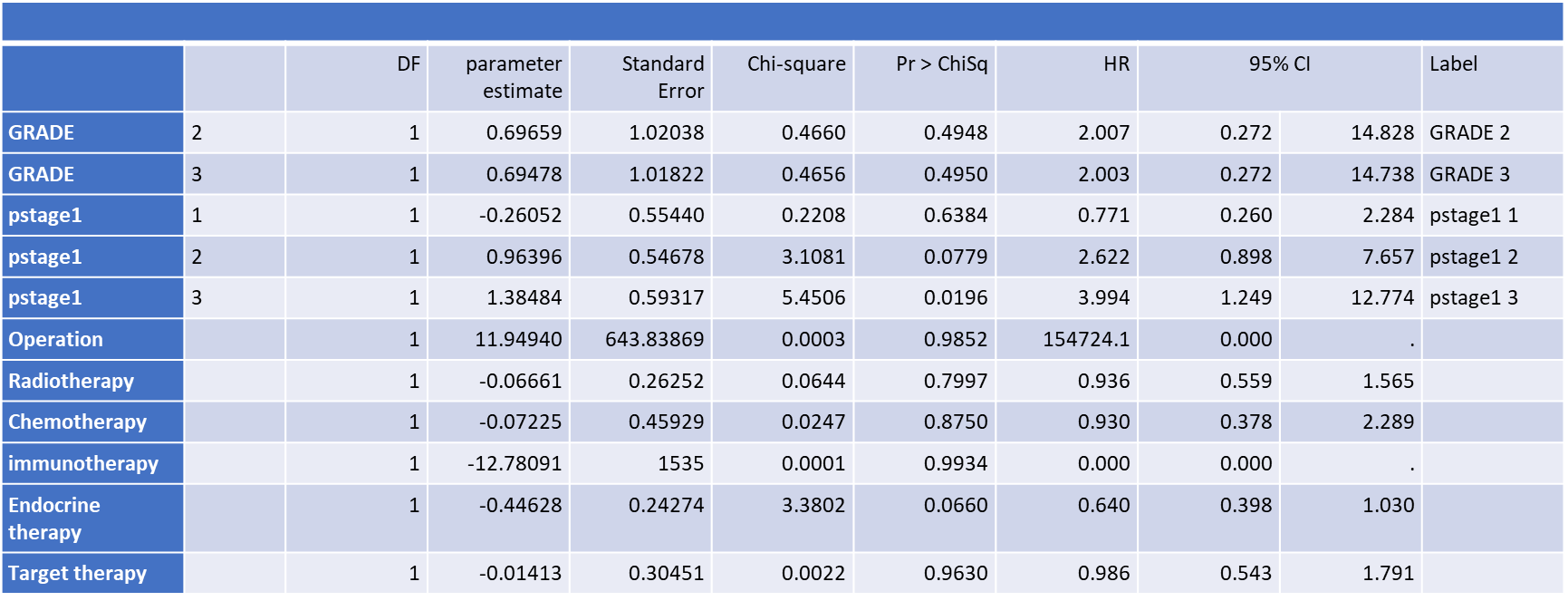


## Table A8e. The breast cancer specific survival outcomes analysis by cox regression model in subgroup of ER 1-9% and PR (+)


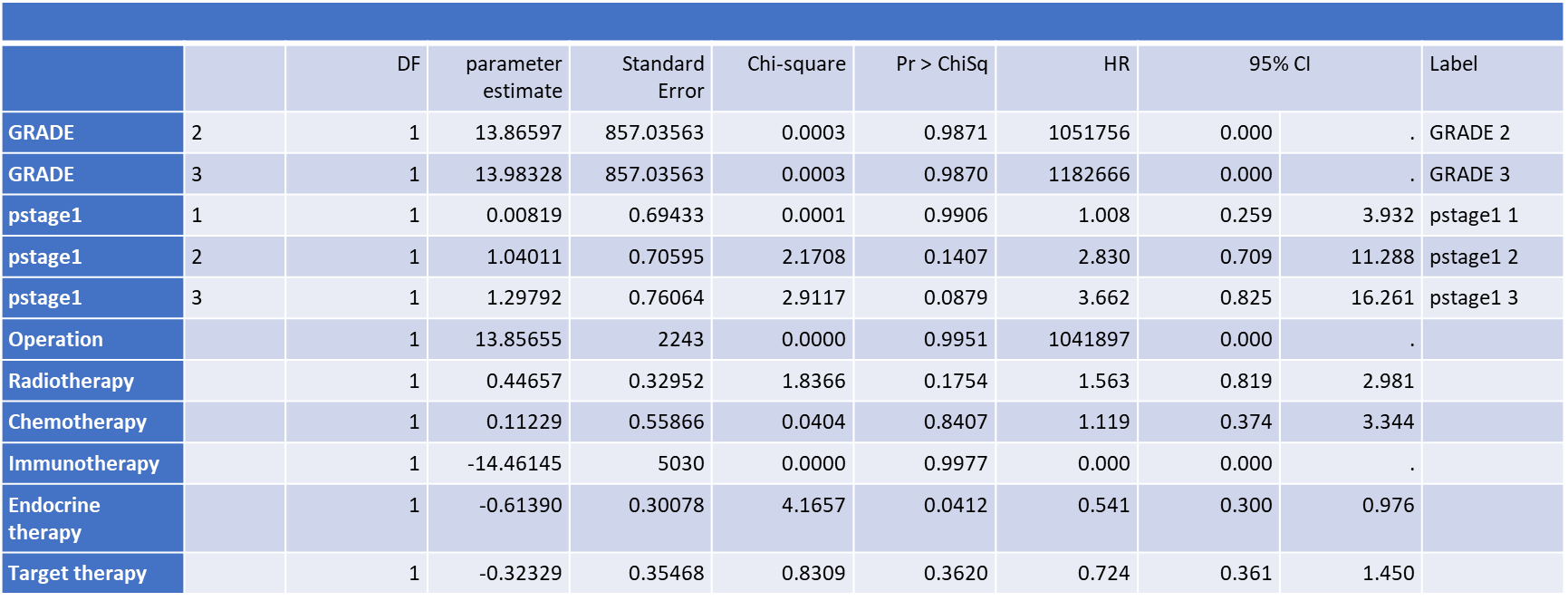


## Table A8f. The recurrence free survival outcomes analysis by cox regression model in subgroup of ER 1-9% and PR (+)


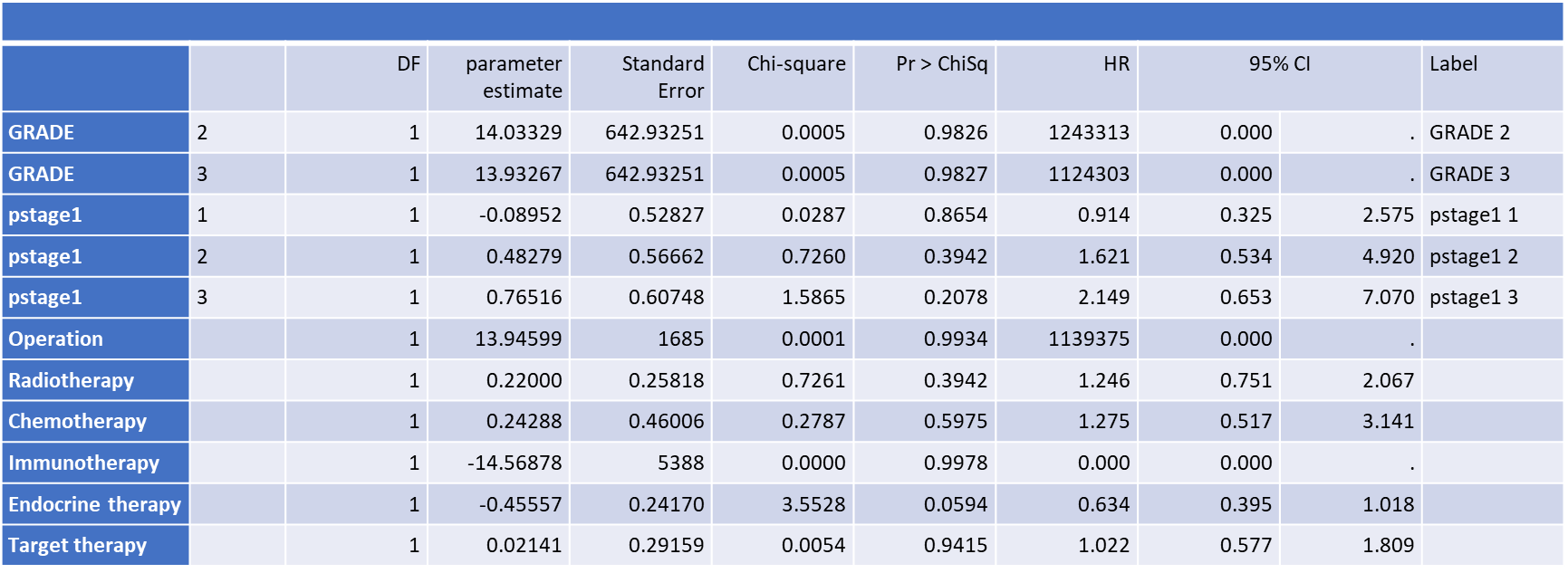


## Table A8g. The overall survival outcomes analysis by cox regression model in subgroup of ER 1-9% and PR (-)


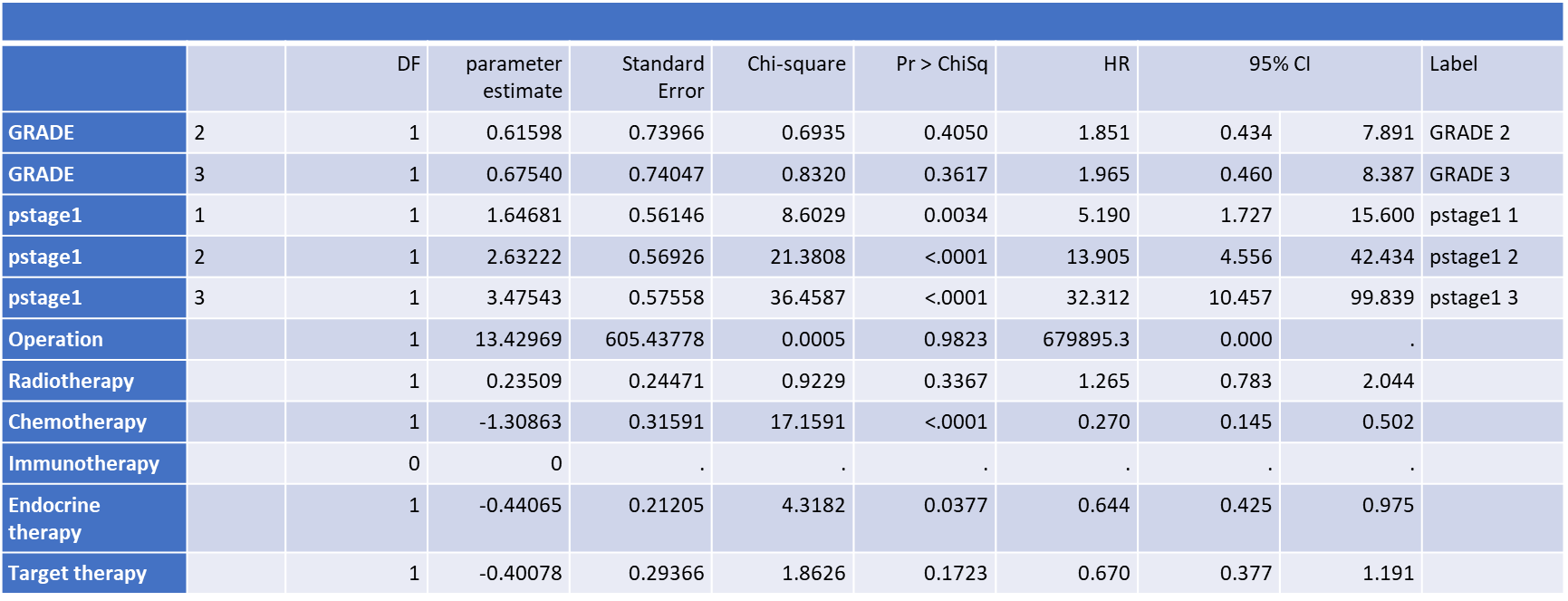


## Table A8h. The breast cancer specific survival outcomes analysis by cox regression model in subgroup of ER 1-9% and PR (-)


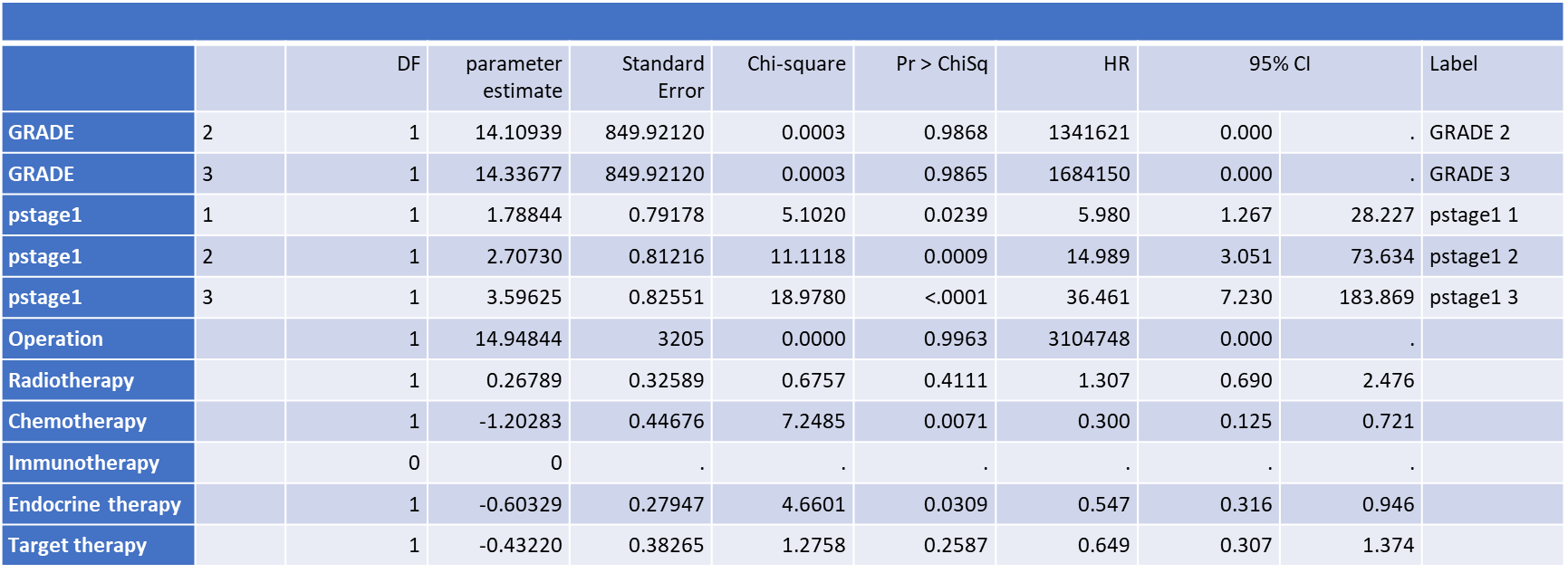


## Table A8i. The recurrence free survival outcomes analysis by cox regression model in subgroup of ER 1-9% and PR (-)


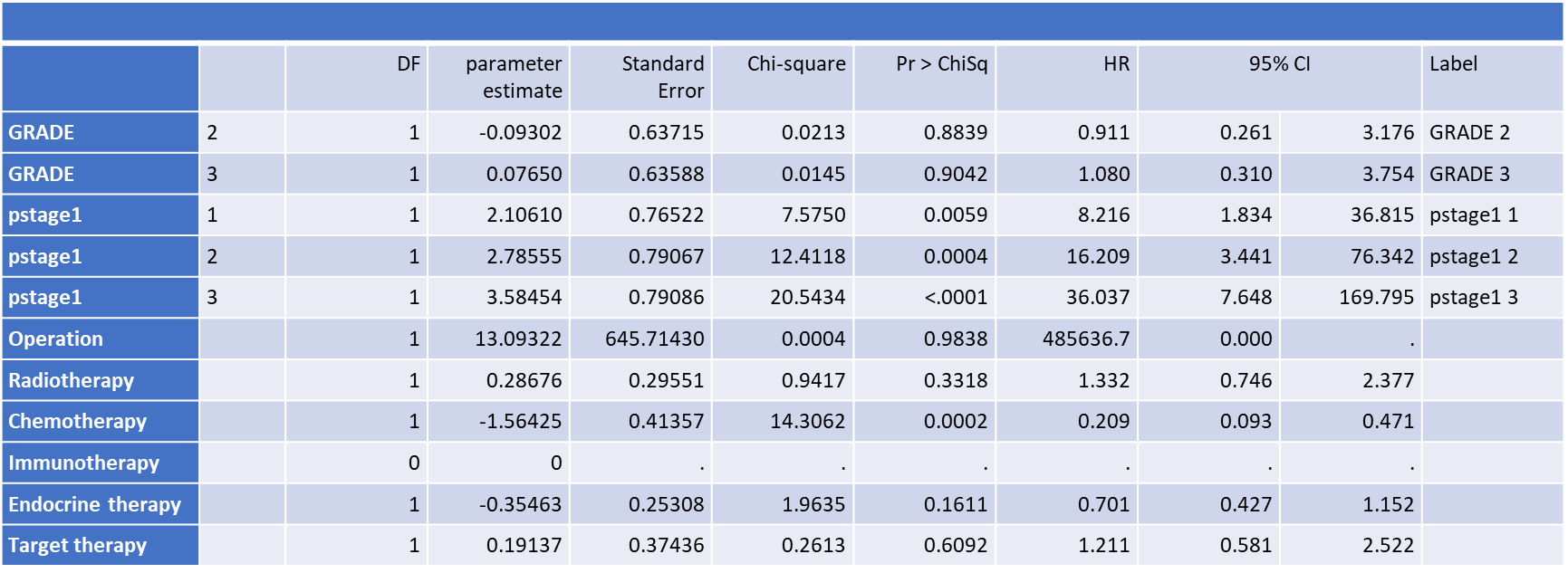


## Table A8j. The overall survival outcomes analysis by cox regression model in subgroup of ER >=10% and PR (+)


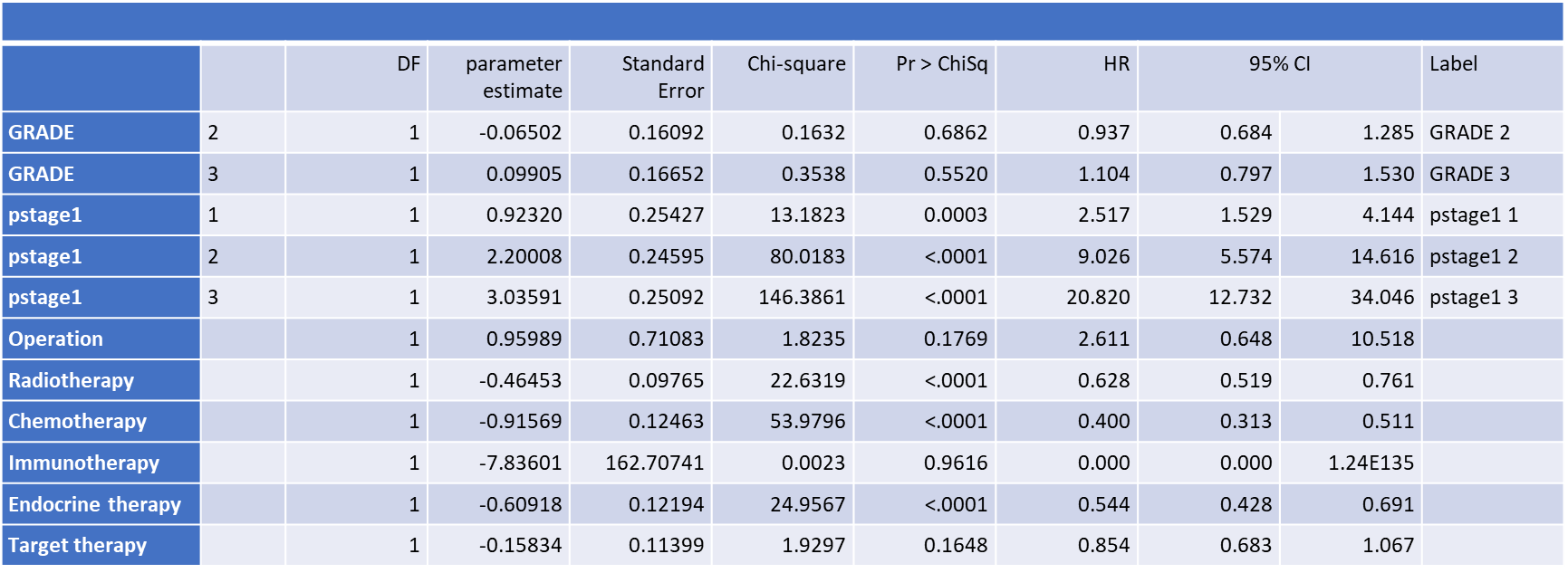


## Table A8k. The breast cancer specific survival outcomes analysis by cox regression model in subgroup of ER >=10% and PR (+)


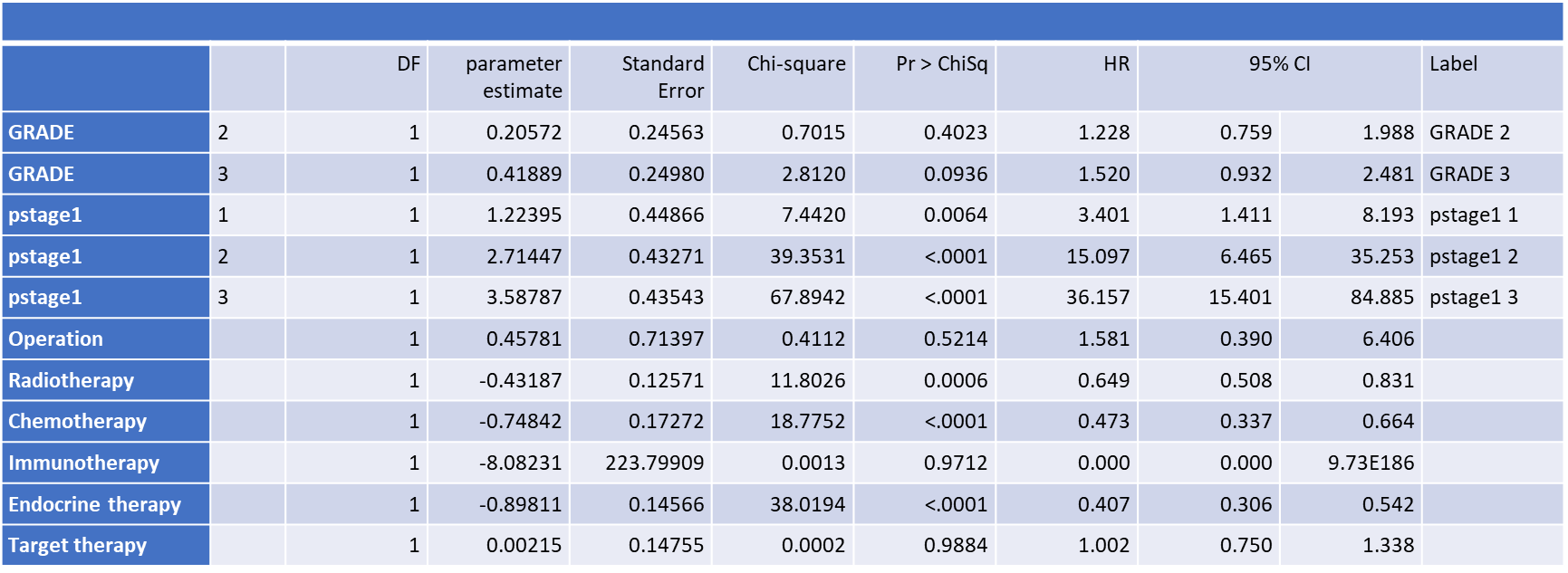


## Table A8l. The recurrence free survival outcomes analysis by cox regression model in subgroup of ER >=10% and PR (+)


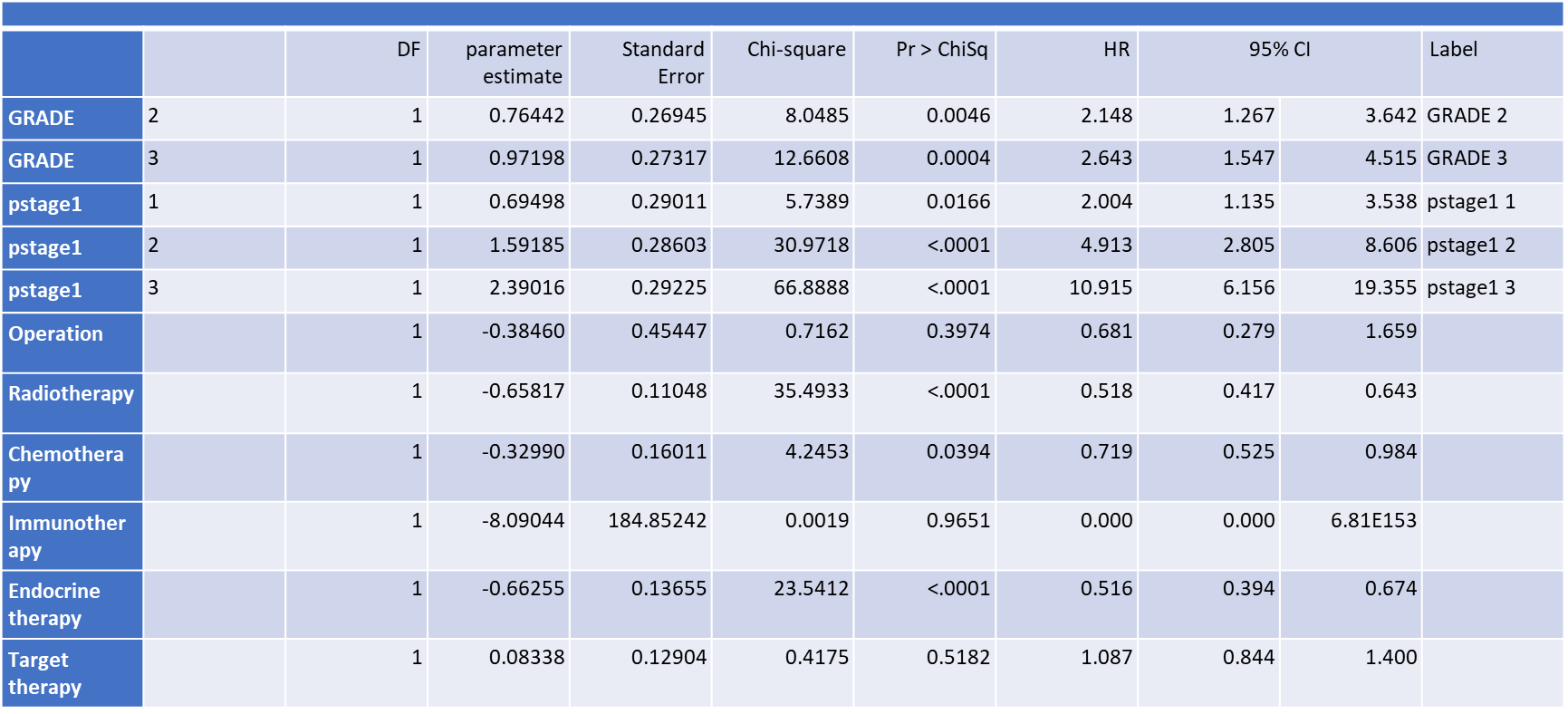


## Table A8m. The overall survival outcomes analysis by cox regression model in subgroup of ER >=10% and PR (-)


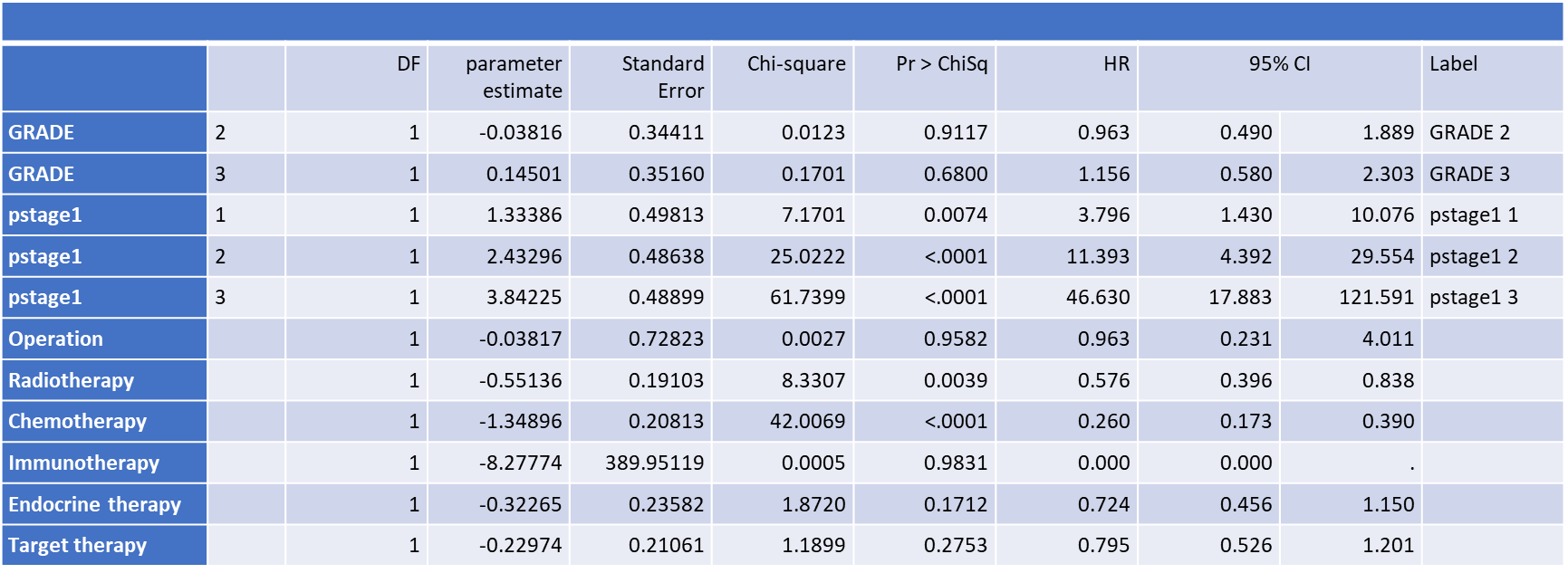


## Table A8n. The breast cancer specific survival outcomes analysis by cox regression model in subgroup of ER >=10% and PR (-)


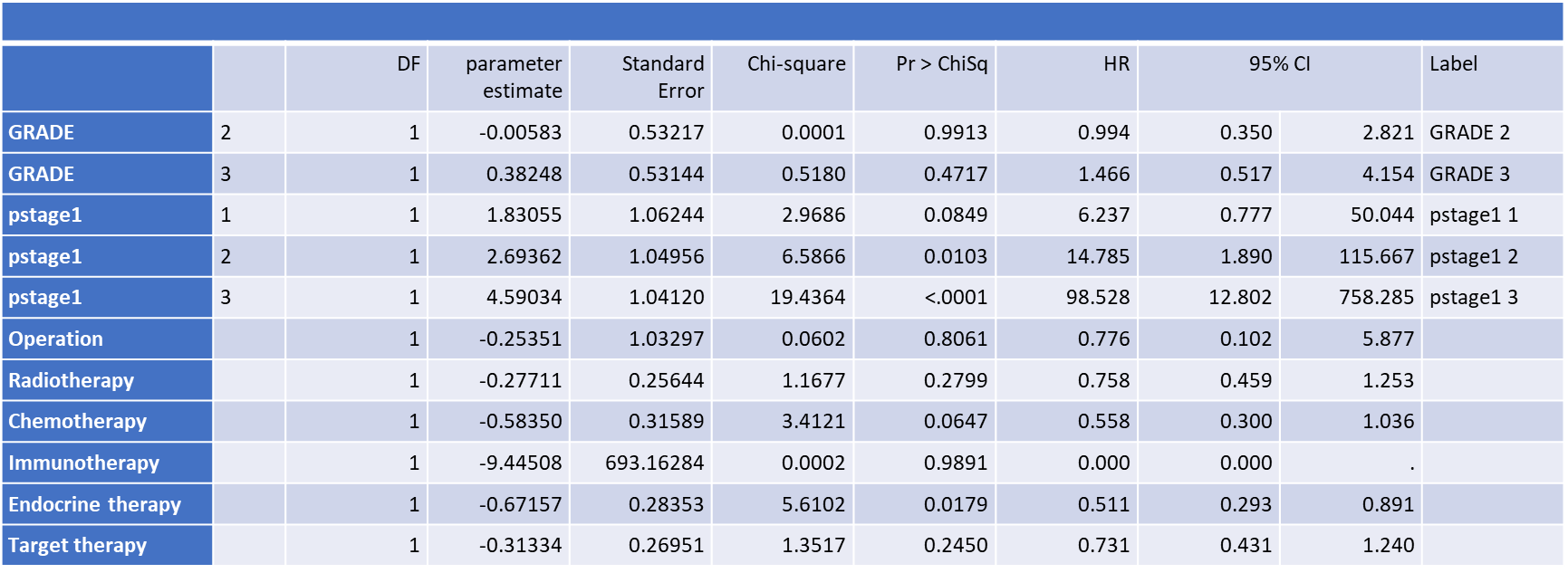


## Table A8o. The recurrence free survival outcomes analysis by cox regression model in subgroup of ER >=10% and PR (-)


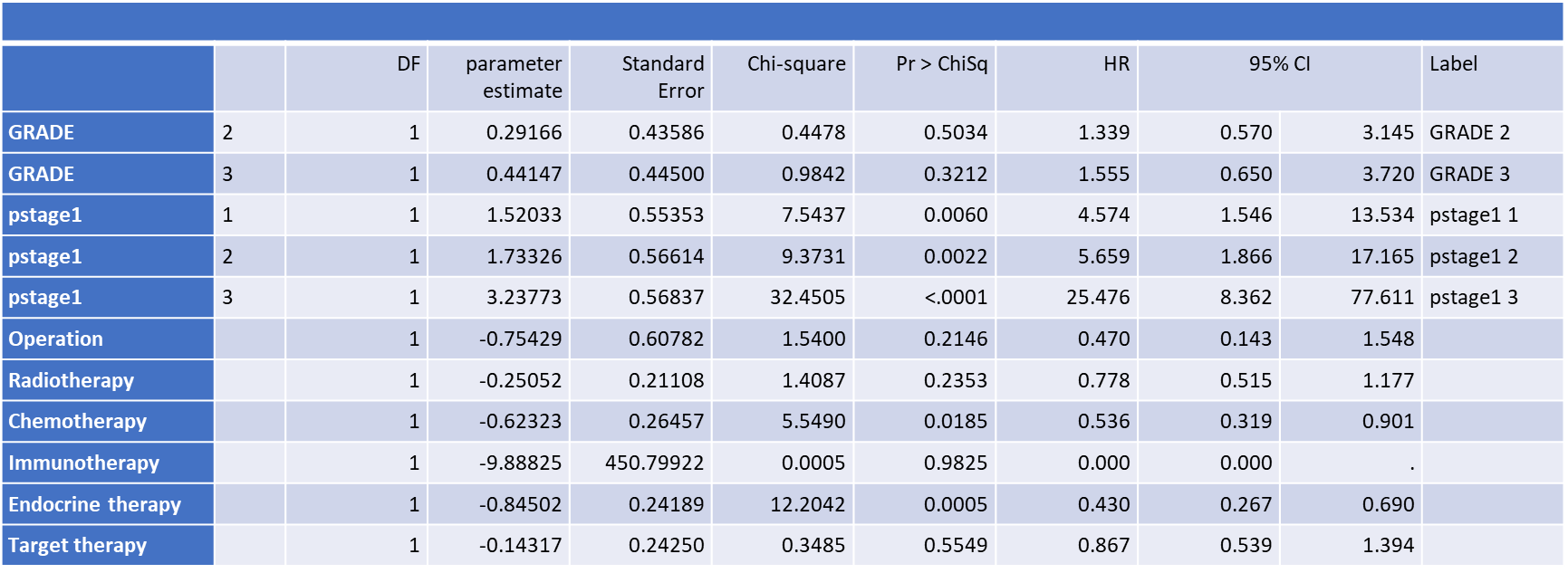


## Table A9. The overall survival outcomes in comparing ultra-low cases with ER 1-5% and ER 6-10%


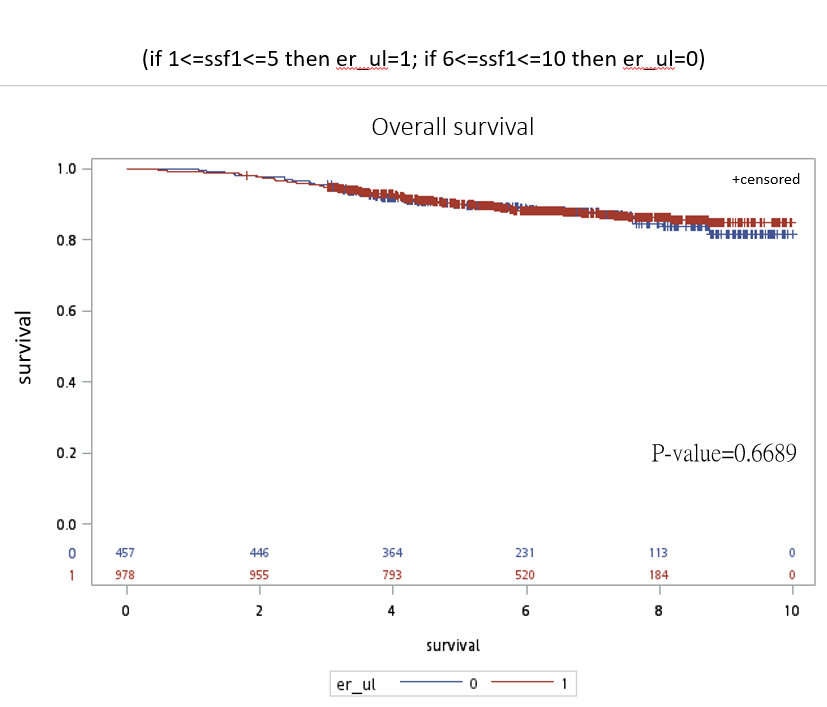


*er_ul =0 means ER 6-10%

*er_ul =1 means ER 1-5%

## Table A9a. The breast cancer specific survival outcomes in comparing ultra-low cases with ER 1-5% and ER 6-10%


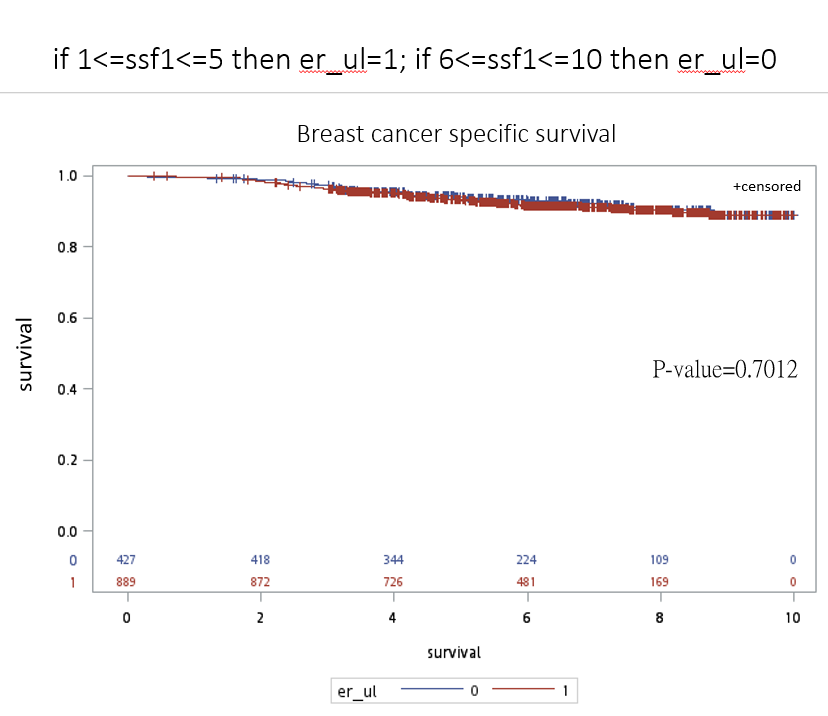


*er_ul =0 means ER 6-10%

*er_ul =1 means ER 1-5%

## Table A9b. The recurrence free survival outcomes in comparing ultra-low cases with ER 1-5% and ER 6-10%


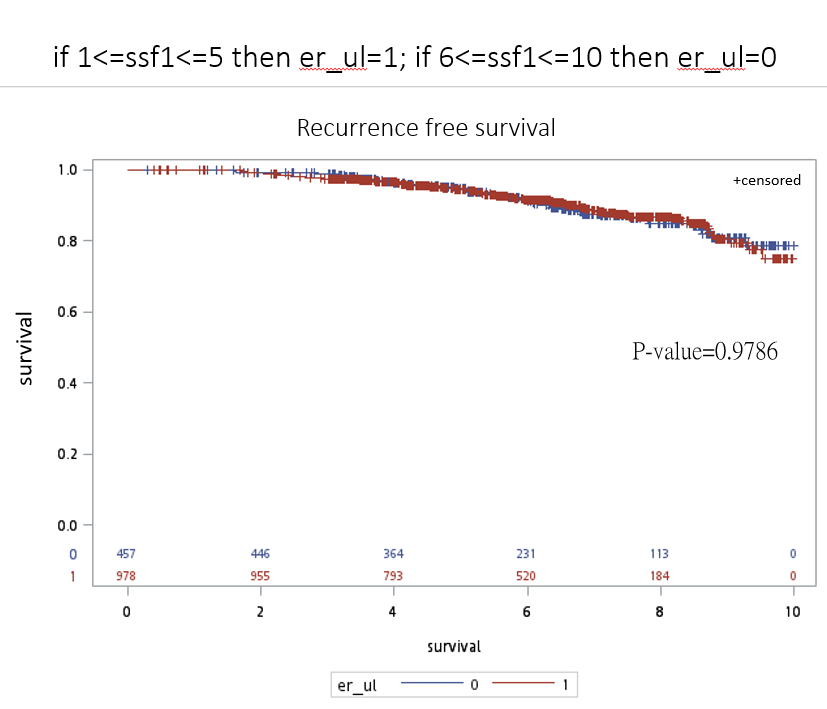


*er_ul =0 means ER 6-10%

*er_ul =1 means ER 1-5%
